# Openly available illustrations as tools to describe eukaryotic microbial diversity

**DOI:** 10.1371/journal.pbio.3002395

**Published:** 2023-11-21

**Authors:** Patrick J. Keeling, Yana Eglit

**Affiliations:** 1 Botany Department, University of British Columbia, Vancouver, Canada; 2 Department of Biology and Institute for Comparative Genomics, Dalhousie University, Halifax, Canada

## Abstract

Microbial life maintains nearly all the support systems that keep the Earth habitable, yet the diversity of this vast microbial world is greatly understudied, misrepresented, and misunderstood. Even what we do know is difficult to communicate broadly because an intuitive grasp of what these tiny organisms are like is abstract, and we lack tools that would help to describe them. In this Essay, we present a series of openly available technical diagrams that illustrate the diverse range of complex body plans of microbial eukaryotes (or “protists”), as well as an illustrated tree to show the vast diversity they encompass and how they are related to the more familiar macroscopic animals, fungi, and plants. These sorts of tools are desperately needed for teaching and communication about the microbial world, which is a pressingly important problem where much improvement is needed.

## Abstract 2

The microbial world is diverse and critically important to understanding evolution and global ecology, but we lack teaching and communications resources. This Essay provides a series of technical diagrams to aid with teaching and communication on the complexity and diversity of microbial eukaryotes.

## Introduction—Describing microbial diversity is challenging but important

Humans have been interested in the idea of “alien worlds” and the strange forms of life that might inhabit them for as long as we have speculated on the nature of the cosmos [1]. Perhaps the most striking feature of the alien life we have conjured up over the centuries is our lack of imagination: extraterrestrials are most often pictured as lightly edited derivatives or fusions of common terrestrial plants and animals, much like the “monsters” of antiquity. It is ironic, then, that we have committed so much of our imagination to envisioning these distant worlds that we seldom appreciate the almost equally alien world that exists right under our noses: the microbial world.

Microbial life shares a common ancestry with all other life on Earth, and therefore, shares a great deal of its fundamental biological machinery with the more familiar macroscopic plants and animals. But microbial life is nevertheless an abstract concept to imagine. This is because, although microbes share the planet Earth with us, they nevertheless live in a different world, and it is a world we have a difficult time comprehending at a basic factual level, much less at an intuitive level. Understanding this microbial world means coming to grips with numbers so vast or so minuscule that they are well beyond our everyday grasp. Microbes are very small of course, but at a scale well beyond our imagination [2]. A single microbial species can reach population sizes that number in the trillions (and collectively there are more microbes on Earth than stars in the known universe), and the number of microbial species is unknown and perhaps unknowable (we do not even really know if most can be said to have “species” in the way we generally define them), but at the very least they outnumber the already large number of macroscopic species by orders of magnitude (this number has been occasionally been estimated to be very small compared to that of animals [3], but this is objectively impossible when we already know the number of parasites alone probably outnumbers the animals they parasitize, and this does not even begin to count free-living microbes).

The scale of these differences is indeed so great that they affect the way microbial life interacts with the physical world, the way they behave, and the way they evolve. Characteristics we alternatively take for granted or completely ignore, like viscosity, hydrophobicity, chemical gradients, electrical charge, or gravity, can have completely different effects on microbial life [4]. They “see,” “hear,” “smell,” and “feel” their environment, but how they process such sensory information is seldom known, although one can readily imagine they sense a world very unlike ours.

As challenging as it may be to imagine such a world, it is important, even urgent that we attempt to do so because microbial communities are increasingly recognized to form the foundations of every major ecosystem on Earth and, by extension, to have critical roles in maintaining the connected networks and systems that make all other life possible [5,6]. It is an unfortunate result of history that microbes are primarily associated with disease, and even today the majority of our research focus is on pathogenic microbes. The growing interest in “microbiomes” has led to a greater appreciation for the positive effects of microbial life, but once again with a focus on direct benefits to humans [5]. These effects, both positive and negative, are very important, but arguably the greatest impact that microbial life has on human wellbeing is though the much larger but more diffuse effects of microbial ecology on the environment as a whole. This is because the vast numbers of diverse microbial species make up a giant, global machine that is constantly recycling nutrients, converting energy, and moving both to every corner of the complex food webs on which all life depends. Elementary nutrient cycles (e.g., nitrogen, sulfur, phosphorous, and carbon) that are the backbone of living systems are primarily, or even entirely driven by microbes [6–8]. In short, if all macroscopic animals and plants were to suddenly vanish, most microbial ecosystems would have to change somewhat in order to adapt, but if the converse happened, and all microscopic life died, everything else would quickly follow.

## Protists—the microbes that harnessed morphological and behavioral complexity

**Fig 1:**
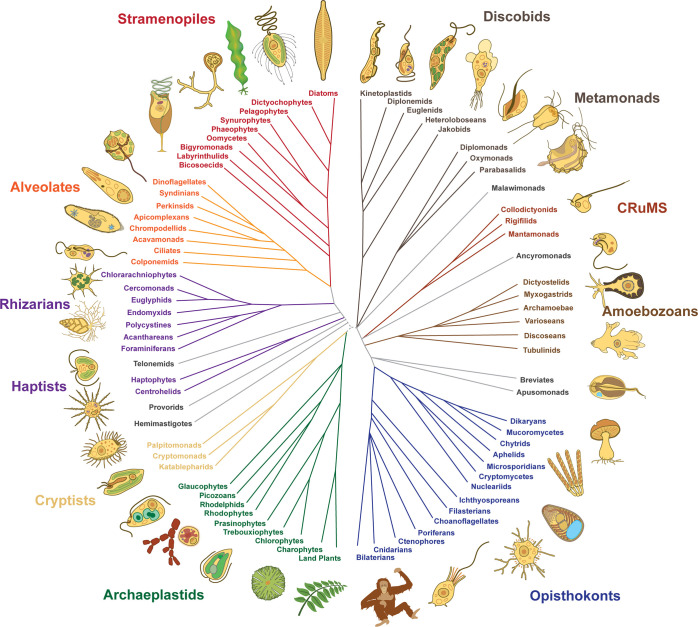
Tree of eukaryotes showing major subgroups. Tree of eukaryotes showing major subgroups and thumbnail diagrams of representative members of each group. The tree is an updated synthesis based on recent phylogenomic reconstructions (e.g., reviewed in [11]). Thumbnails are also provided separately in S1 File, where they are laid out in isolation from one another for ease of use individually.

Although we have stressed the practical importance of the microbial world, it is worth emphasizing that it is also wondrously diverse esthetically, with all the complexity, beauty, and drama we are accustomed to finding in nature documentaries. This diversity is most obvious in the protists, a generic term for eukaryotes (cells with a nucleus) that are not plant, animal, or fungus. The vast majority of protists are microbial (exceptions being kelps and other seaweeds, for instance), and collectively, they make up most of the diversity of the eukaryotic tree of life (Fig 1). Protists buck the misperception that microbes are structurally simple: They are masters of structural and molecular complexity, with even single cells ranging in size over orders of magnitude and employing a wide variety of body plan principles, from asymmetry to symmetry, along multiple axes (for examples from a single lineage, see the parabasalian protists [9,10]).

Protists span an equally wide range of life strategies readily comparable to multicellular analogues, from plant-like photosynthetic autotrophs to predatory hunters with the ruthless efficiency of cats. But protists can also take all strategies to greater extremes (for example, engulfing food particles larger than their own body or latching onto prey and sucking their contents out through a straw), or even combine them: mixotrophs can switch between photosynthesis and hunting down and consuming prey, or do both at once [6,12]. Protists can also utilize multiple developmental switches to transition through complex life cycles, with life stages so different from one another that they were once mistaken for completely different lineages. They are behaviorally diverse, using subcellular structures to sense and respond to stimuli like gravity, light, or nearby predators or prey, and move using flagella, pseudopodia, gliding motility, or even walk on legs made from bundles of cilia. They can assemble gun-like structures to fire toxic projectiles for both offense and defense, construct mouths to eat other cells, build armor and spines to protect them from being eaten, and can develop entire digestive tracts [13–17]. And all of this is carried out without the benefit of tissue differentiation: it is all contained within a single cell.

Because they are also particularly understudied, protist diversity provides a rich vein of interesting new biology to explore, placing the field at an interesting intersection with a 19th century scope for exploration meeting 21st century tools. But, at the same time, this also means protists are especially lacking in both a widespread awareness of their fundamental properties (e.g., most biology undergraduates will not be aware of what they are) and tools to describe them (e.g., the most recent English-language protistology textbook was translated from German and published 20 years ago, when protists were barely entering the age of molecular biology and genomics [18]). Paradoxically, this makes the great diversity of protist form, structure, and behavior simultaneously one of their most interesting facets, but also their most daunting to communicate.

## A picture can say a 1,000 words or it can make things even more confusing

If you try to explain something about the biology of trees or squirrels, your audience can be expected to have an immediate and intuitive image in their minds of these organisms that sets the stage for any further information about them. For most microbes, however, this is not the case. If you start to explain something about the biology of euglenoids or radiolaria, you are more likely to get a blank look. If you say microbes, the image conjured up is most likely a blob, perhaps with flagella, because this is what textbook drawings of microbes tend to look like. In fact, very few eukaryotic microbes look like blobs; most are rather complex cells that result from what must be sophisticated (but mostly unexamined) developmental pathways leading to species every bit as structurally distinct as animal or plant species.

These complex forms can be observed by microscopes and captured by photography, and while the resulting images can be as beautiful as those of birds, fish, or flowers [19], they often fail to convey what distinguishes one kind of protist from others in the way they can for animals or plants. That is because our concepts of what defines one group of protists from others nearly always crosses many spatial scales, from molecules to gross morphology, and the data on which this is based are abstract, not readily intuitive, and require years of training to interpret. For example, a simple photograph summarizing the shape, size, and color of a protist taken using even advanced light microscopy will yield a relatively straightforward picture of these basic features, but for many organisms even an expert might remain unsure about what kingdom it belongs to without more data. This can be ultrastructural data based on transmission electron microscopy (TEM), which is the level of resolution at which many of the defining features of protist lineages are distinguishable. But generating TEM data and especially interpreting it are highly specialized skills requiring years of training. The most common means to identify protists now is molecular sequence data, but these are also abstract and complex data to interpret, and also tell us nothing directly about the physical organization of the cell.

The problem is therefore one of synthesizing a mix of relatively intuitive data with data that require extensive training to interpret, which is most easily accomplished with technical diagrams. A diagram can average over or synthesize the variation from one specimen to the next and emphasize the features that really distinguish a species or group in a simple way that raw data can seldom match. Even for organisms where these problems are relatively minor (e.g., birds, mammals, or fish), the best field guides generally rely on drawings and paintings rather than photographs for exactly this reason. Imagine, then, if your favorite field guide to birds had to include detailed anatomy of internal organs, in addition to size, shape, and color, all in a single photograph.

The field of protistology, and arguably all microbiology, needs more simple aids to communicate what these organisms are “like” at a basic level. A good place to start would be tools to clearly describe the basic features of major protist groups, to quickly communicate what defines each lineage, what makes it unique, and what differentiates it from another given lineage. In short, we need tools to quickly and accurately create the mental image of the organism that comes automatically for squirrels and trees. As a small, but hopefully useful first step, we have created a series of technical diagrams of protist body plans representing members of every major supergroup of protists, scattered around the tree of eukaryotes (Fig 1), and below describe some of the principles we tried to adhere to and how these might be used.

## Morphological diversity of protists in body plan diagrams

**Fig 2:**
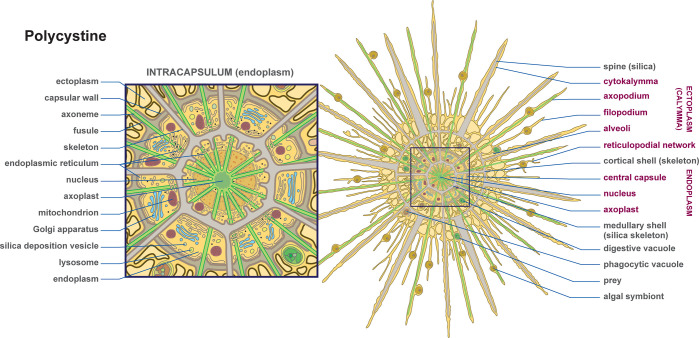
A complex body plan diagram of a polycystine (radiolarian) protist. These large cells have a complex internal skeleton and structural organization that is difficult to capture in a photo or electron micrograph. Here, we also zoom into the central portion of the cell to highlight the organization. This image is provided in the supplemental files in PDF, SVG, and JPG formats, with and without labels.

**Fig 3:**
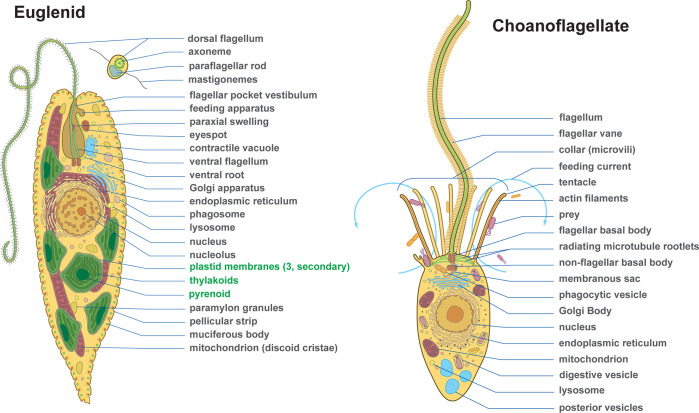
Body plan diagrams of a photosynthetic euglenid and a heterotrophic choanoflagellate. Examples of body plan diagrams from a photosynthetic euglenid (right) and heterotrophic choanoflagellate (left). These images are provided in the supplemental files in PDF, SVG, and JPG formats, with and without labels.

**Fig 4:**
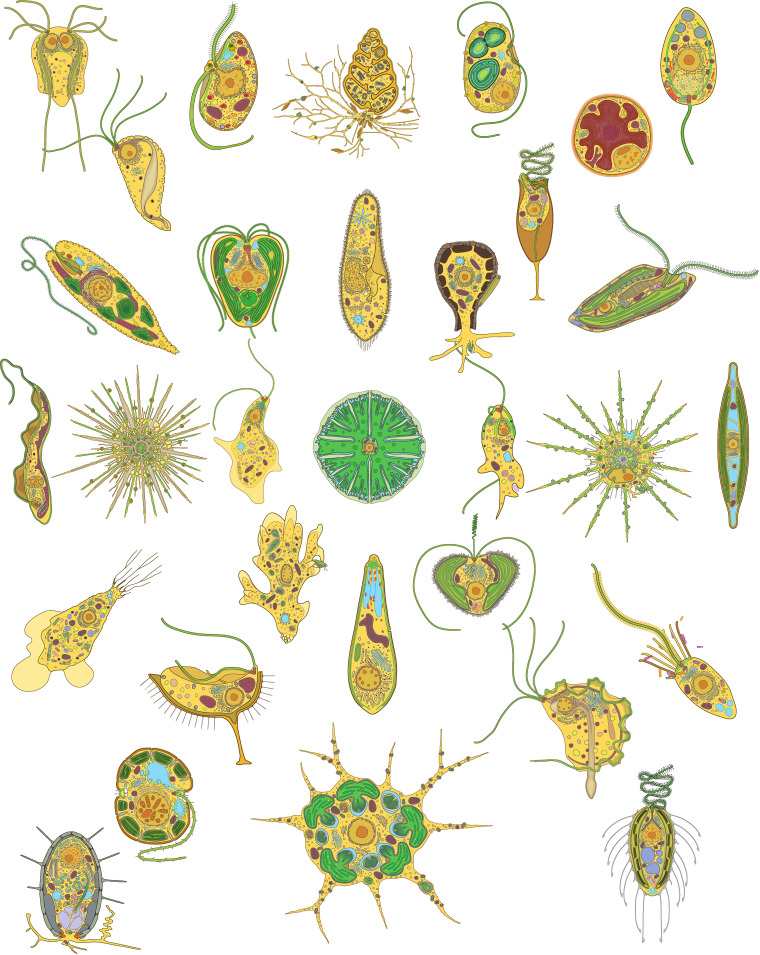
A collage of cell diagrams showing overall protist diversity. The images of cells shown are all provided in high-resolution in supplemental files in PDF, SVG, and JPG formats, with and without labels. This collage is also provided with a black background in the supplemental files.

In Figs 2–4 and in S1–S6 Files, we provide 33 diagrams, each representing a major lineage of eukaryotes. Their position in the tree of eukaryotes is illustrated in Fig 1. Each diagram shows the overall shape of the cell and outlines the major features of its body plan, including common organelles like the nucleus, mitochondria, plastids, flagella, and basal bodies, as well as any more unique and distinctive morphological and ultrastructural features used to identify or define the lineage. All images are available in JPG and SVG formats (compressed) and as a single PDF for quick reference. All are provided both with and without descriptive labels highlighting the main features of the cell. In addition, we also provide simplified thumbnails to represent the diversity of eukaryotic forms in a smaller format where the higher level of detail would not scale down sufficiently. As an example, we used the simplified thumbnails to illustrate the diversity across the tree of eukaryotes and the distribution of body plans across various major lineages in Fig 1. All these images are under a Creative Commons Attribution (CC BY) license, and so are free be used, modified, or distributed as long as the original source (this publication) is attributed. All images have also been deposited and are publicly available in Zenodo [20].

The purpose of these diagrams is to clearly convey a large amount of information to a nonexpert, a goal that faces 2 challenges in particular: the vast diversity of protists and their individual complexity. To balance these, we followed a few general principles in deciding which organisms to select and how to represent them.

## Representing diversity

A selection of even hundreds of species would barely scratch the surface of protist diversity, but would at the same time also overwhelm with details. As a starting point, we sought to represent as many major groups as possible, taking into consideration which groups were known to be abundant, ecologically and/or evolutionarily significant, and relatively well-studied. The goal being to make the diagrams useful to those seeking to show diversity across the tree, or to compare body plans between major groups in general, while at the same time providing diagrams representing many of the best-studied groups that can be used individually to familiarize an audience with that subject group (for example, to help orient the viewer when paired with photographs). For balance, the collection includes at least 1 representative from each major eukaryotic “supergroup” (see Fig 1) as well as representatives of most particularly well-studied lineages.

## Generalizing defining characteristics

Any diagram representing a group as massive and diverse as those chosen here (many of which are vaguely like a “kingdom” of life) is bound to be a generalization, regardless of how understudied the group is. Two approaches to this problem are to select an organism that best represents its close relatives or show an idealized form that represents the features defining the group. Where possible we chose the former, but nevertheless refrained from labeling diagrams with specific taxonomic names, only providing names of the larger group to which they represent. Experts may recognize particular genera among the collection, but as these are aimed at the nonexpert, more formal names and subdivisions would be an unnecessary distraction and added terminology. Moreover, in many lineages, the best studied taxa are poor representatives of the body plan most common to the group; for example, *Giardia* is the best studied diplomonad because of human medical significance, but its body plan is highly reorganized compared with most members of the lineage and would be a poor representative for the group as a whole. We also aim to make the images useful on their own and not just as a collection, so have refrained from cross-referencing between groups. We have also not added scale bars, although we do recognize that size is important, since the images are meant to represent a range of species in a large group, and size like other characters does vary between them. Suffice to say, these organisms are mostly quite small.

## Including features consistently across lineages

There are many cellular features that do not distinguish a particular lineage, but are common to many kinds of eukaryotes. Others are common to many but not all eukaryotes and can be part of a suite of characteristics that only collectively define a lineage. All this complexity is an important aspect of eukaryotic cells in general, and so we have shown several such features consistently across diagrams, regardless of their relevance to a given lineage and what defines it. This is important to show where variation in the feature exists, while at the same time making the drawing intelligible on its own and reminding the viewer of the baseline of complexity in the eukaryotic cell.

## What else is to be done?

Obviously, a handful of technical diagrams of protists is a small drop in the bucket when held up to the massive diversity of the microbial world and the challenges we face in describing and communicating it to nonexperts. So it is worth emphasizing that, however small this step may be, it already fills a hole, since even such basic tools are not already easily available. This is some indication of how badly underdeveloped these resources are, which is a serious problem: drawing new talented and energetic researchers to a “non-mainstream” field already requires extra effort to capture the imaginations of students who are in all likelihood completely unfamiliar with the organisms and questions surrounding them [5]. A handful of diagrams may be useful, but we require a deep and sustained effort to develop tools on multiple different fronts.

The problems associated with communicating the complexity and diversity of the microbial world are not unique to the study of nonpathogenic microbes or protists, so we might look to other fields that have been much more proactive and successful in the area of communication to see what actually works. One obvious source is other areas of biodiversity, but communication about microbial diversity shares a surprising number of challenges with astronomy and cosmology: they have similar problems of unimaginable scope and scale; they share a strong tradition of research based on exploration (in part due to the scale); and both require imagination to see past the abstract and otherworldly nature of the subjects. But whereas microbial diversity remains in the shadows and research focusing on it requires constant justification, exploring the cosmos is widely regarded as a significant human endeavor. Drawing on all these other fields, we can see a few obvious directions that would likely have significant impact.

## New textbooks and introductory courses

This sounds ridiculously obvious, but without a textbook, a field is always going to be neglected. We engage students early through our formal teaching, and without an easily available textbook it is difficult to teach an introductory course, even for an expert. Without introductory courses, most students are not even aware the field exists. Few introductory courses focused on protists exist today, anywhere in the world, making them probably the least-covered slice of the tree of life (perhaps tied with Archaea). A good, free, and widely available textbook would make teaching such a course more accessible to even nonexpert instructors and would make taking such a course more accessible to students. Expert curation probably plays an important role here, but open-source teaching materials are also powerful, and available platforms like Wikipedia have already been adapted for biodiversity.

## Scholarly organizations can have a role

The internet offers an alternative to traditional textbook publishing, which for fields outside the mainstream has historically led to no publishing at all, or to small, expensive, and short-lived runs that have little long-term impact on teaching. It is a good time to re-think how to produce and disseminate textbooks. Producing a text may require the knowledge and hard work of the experts writing it, but there are still significant gaps traditionally provided by publishers, in particular in production (e.g., graphics) and dissemination. Scholarly societies could have an outsized role in their field simply by filling these gaps. They could support professional illustration and graphic design and could host the final product in perpetuity on their website for free distribution (as the International Society for Protistologists recently did in freely hosting the Illustrated Guide to the Protozoa). Such actions would truly be an invaluable contribution to the field they represent and would also increase the society’s profile both inside and outside the field.

In the non-virtual world, academic organizations can also have a role, particularly those with already strong public outreach activities. Most natural history museums represent microbial diversity very poorly, with microbes occasionally as a footnote in a few displays, and predictable biases shown prominently in a “tree of life.” One could argue (and museums do) that you have to give the public what they are already interested in; however, it can also be argued that the job of such institutions is to surprise visitors with a reality that does not match their biases. Evidence that this can be effective is on prominent display in Amsterdam’s engaging and, to our knowledge, unique museum of microbes, Micropia.

## Reiterating the importance of video and graphical images and community engagement

We have focused largely on illustrations and made a case specifically for technical diagrams, but microscopic images are also valuable. Image databases have been promoted in the past, but many are static and lack community engagement. New generations of databases with more focus on synthesizing different kinds of information have the power to revive this effort, and the internet offers other models to encourage engagement even more widely. One very popular model is based on community involvement and discussion, for example, iNaturalist, and there is no reason such a model cannot extend to microbial life and microscopy.

But even more importantly, it would be a huge mistake overlook the greater power of video, especially on the internet. And by video, we do not just mean video microscopy; CGI animation is one of the most effective ways to convey an alien world, and this has been actively embraced by agencies trying to communicate the nature of other alien worlds, like NASA. The videos explaining new advances in space exploration are often partially or entirely animations, because they are much more effective communication tools for all the reasons discussed above. Realistic, immersive video of the microbial world has only rarely been attempted, and never with the kind of support that leads to the high production standards we see from NASA. It stretches ones imagination to even wonder, with such resources, how could the microbial world be portrayed? This is not to say that engaging people requires NASA-levels of funding, simply telling compelling stories in text, pictorial, or video formats all play a part. More active engagement is also not dependent on access to research-grade microscopy. The cameras and screens of millions of smartphones have made possible a variety of fun, DIY microscopy options, ranging from free to nearly free (e.g., Foldscope, imicro-scope).

## Conclusion

None of these problems are easy to solve, but they are not impossible. The microbial world has the raw material for stories every bit as compelling as the lions, gazelles, and even the grass of a Serengeti nature documentary, and more compelling than the monsters of antiquity or even the science fiction ones we invent today. These are biologically interesting and esthetically fascinating organisms of global ecological importance; if we do not spark the curiosity of the public to learn more about the microbial world, or even young scientists thinking of a potential research career in the field, then the failure is ours.

## Supporting information

S1 File (PDF)

S2 File (PDF)

S3 File (PDF)

S4 File (PDF)

S5 File (ZIP)

S6 File (ZIP)

## References

[1] Roush W. Extraterrestrials Cambridge MA. MIT Press; 2020.

[2] Morrison P, Morrison P. Powers of Ten. W.H. Freeman & Company; 1982.

[3] Mora C, Tittensor DP, Adl S, Simpson AGB, Worm B. How many species are there on Earth and in the ocean? PLoS Biol. 2011;9:e1001127. doi: 10.1371/journal.pbio.1001127 21886479 PMC3160336

[4] Purcell EM. Life at low Reynolds numbers. Am J Phys. 1977;45:3–11.

[5] Timmis K, Cavicchioli R, Garcia JL, Nogales B, Chavarría M, Stein L, et al. The urgent need for microbiology literacy in society. Environ Microbiol. 2019;21:1513–1528. doi: 10.1111/1462-2920.14611 30912268

[6] Worden AZ, Follows MJ, Giovannoni SJ, Wilken S, Zimmerman AE, Keeling PJ. Rethinking the marine carbon cycle: factoring in the multifarious lifestyles of microbes. Science. 2015;347:1257594. doi: 10.1126/science.1257594 25678667

[7] Falkowski PG, Fenchel T, Delong EF. The microbial engines that drive Earth’s biogeochemical cycles. Science. 2008;320:1034–1039. doi: 10.1126/science.1153213 18497287

[8] Jansson JK, Hofmockel KS. Soil microbiomes and climate change. Nat Rev Microbiol. 2020;18:35–46. doi: 10.1038/s41579-019-0265-7 31586158

[9] Brugerolle G, Lee JJ. Phylum Parabasalia. In: Lee JJ, Leedale GF, Bradbury PC, editors. An illustrated guide to the protozoa. Society of Protozoologists. 2000:1196–1250.

[10] Cepicka I, Hampl V, Kulda J. Critical taxonomic revision of Parabasalids with description of one new genus and three new species. Protist. 2010;161:400–433. doi: 10.1016/j.protis.2009.11.005 20093080

[11] Keeling PJ, Burki F. Progress towards the tree of eukaryotes. Curr Biol. 2019;29:R808–R817. doi: 10.1016/j.cub.2019.07.031 31430481

[12] Selosse M-A, Charpin M, Not F. Mixotrophy everywhere on land and in water: the grand écart hypothesis. Ecol Lett. 2017;20:246–263. doi: 10.1111/ele.12714 28032461

[13] Larson BT, Garbus J, Pollack JB, Marchall WF. A unicellular walker controlled by a microtubule-based finite-state machine. Curr Biol. 2022;32:3745–3757.e7. doi: 10.1016/j.cub.2022.07.034 35963241 PMC9474717

[14] Gavelis GS, Wakeman KC, Tillmann U, Ripken C, Mitarai S, Herranz M, et al. Microbial arms race: Ballistic “nematocysts” in dinoflagellates represent a new extreme in organelle complexity. Sci Adv. 2017;31:e1602552. doi: 10.1126/sciadv.1602552 28435864 PMC5375639

[15] Buonanno F, Anesi A, Guella G, Kumar S, Bharti D, La Terza A, et al. Chemical offense by means of toxicysts in the freshwater ciliate. Coleps hirtus J Eukaryot Microbiol. 2014;61:293–304. doi: 10.1111/jeu.12106 24512001

[16] Plattner H, Kissmehl R. Molecular aspects of membrane trafficking in paramecium. Int Rev Cytol. 2003;232:185–216. doi: 10.1016/s0074-7696(03)32005-4 14711119

[17] Lee JJ, Leedale GF, Bradbury PC, editors. An illustrated guide to the protozoa. Society of Protozoologists: 2000.

[18] Hausmann K, Hülsmann N, Radek R. Protistology. 3rd ed. E. Schweizerbart’sche Verlagsbuchhandlung, Stuttgart; 2003.

[19] Weiss J. The Hidden Beauty of the Microscopic World: What the tiniest forms of life can tells us about existence and our place in the universe. Penguin; 2021.

[20] Keeling P. Technical Diagrams of Protist Cells Zenodo. 2023. doi: 10.5281/zenodo.10018216

