# Supplementary material for: Openly available illustrations as tools to describe eukaryotic microbial diversity
Source: PLoS Biol. 2023 Nov 21;21(11):e3002395. doi: 10.1371/journal.pbio.3002395 (PMC10662721; doi:10.1371/journal.pbio.3002395)
Supplement: S1 File — (PDF) [file pbio.3002395.s001.pdf]

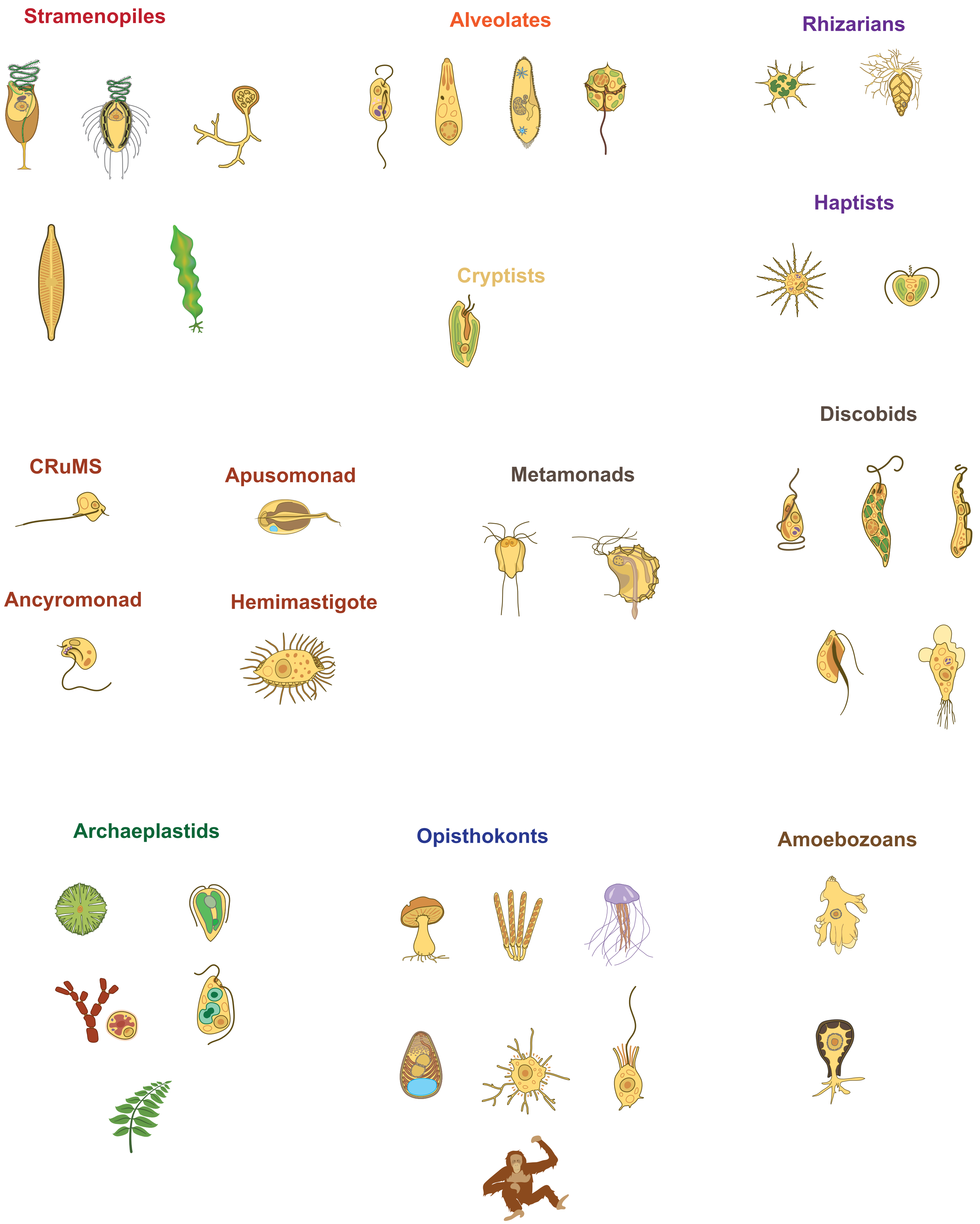
Stramenopiles
Alveolates
Rhizarians
Haptists
Cryptists
Discobids
CRuMS
Apusomonad
Metamonads
Ancyromonad
Hemimastigote
Archaeplastids
Opisthokonts
Amoebozoans
