# Supplementary material for: Openly available illustrations as tools to describe eukaryotic microbial diversity
Source: PLoS Biol. 2023 Nov 21;21(11):e3002395. doi: 10.1371/journal.pbio.3002395 (PMC10662721; doi:10.1371/journal.pbio.3002395)
Supplement: S2 File — (PDF) [file pbio.3002395.s002.pdf]

Kinetoplastid

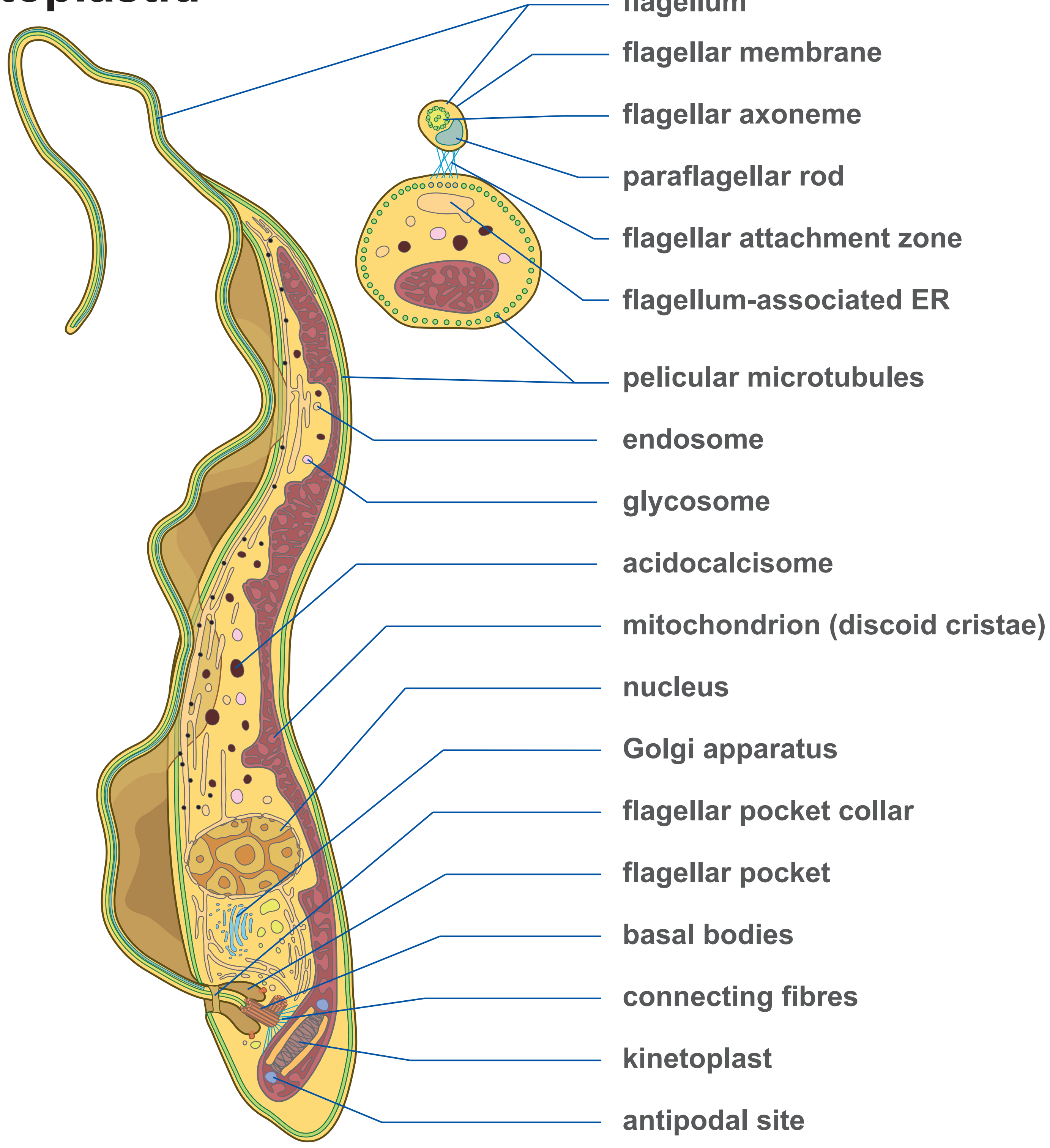

Euglenid

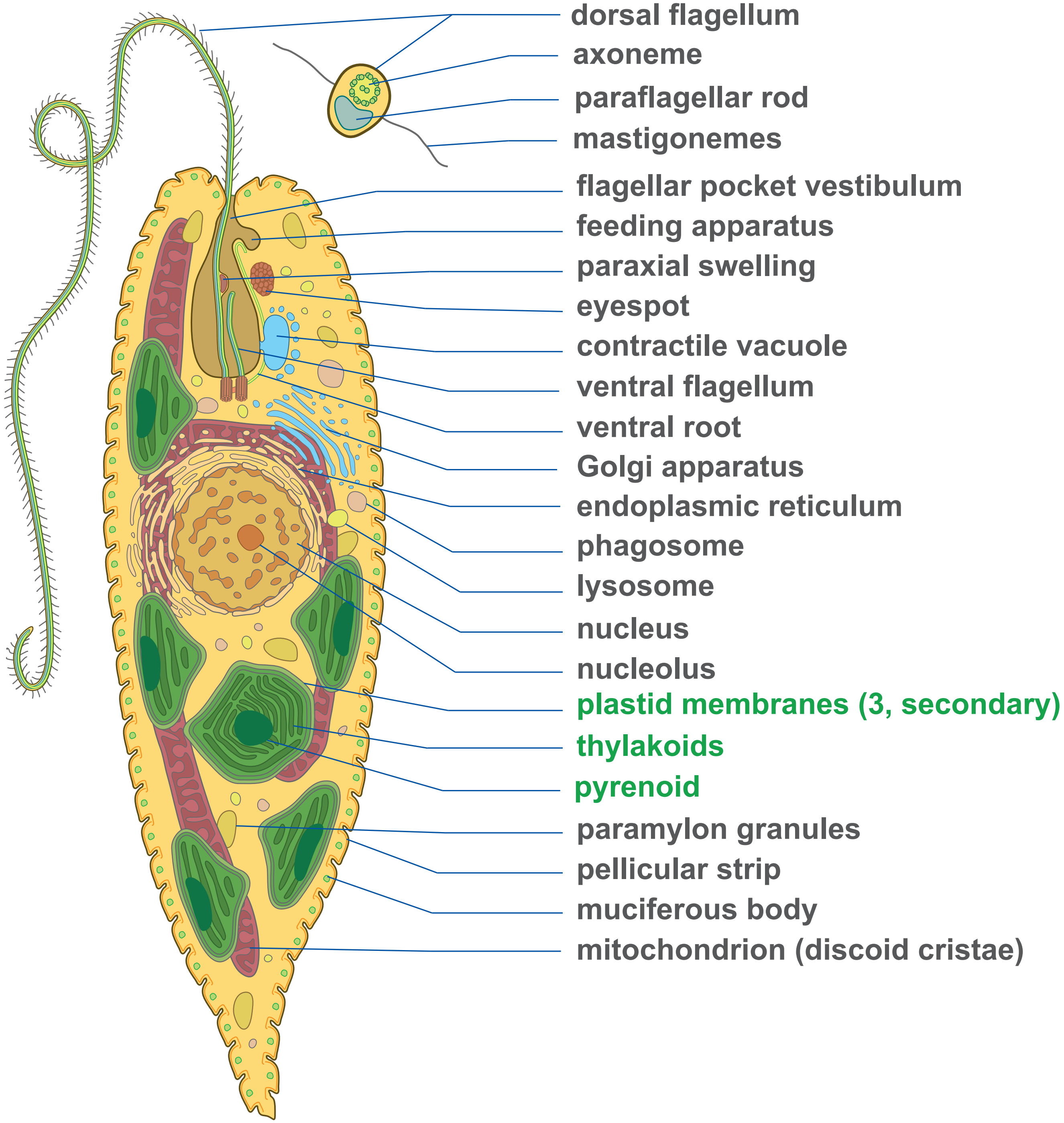
dorsal flagellum
axoneme
paraflagellar rod
mastigonemes
flagellar pocket vestibulum
feeding apparatus
paraxial swelling
eyespot
contractile vacuole
ventral flagellum
ventral root
Golgi apparatus
endoplasmic reticulum
phagosome
lysosome
nucleus
nucleolus
plastid membranes (3, secondary)
thylakoids
pyrenoid
paramylon granules
pellicular strip
muciferous body
mitochondrion (discoid cristae)

Heterolobosean

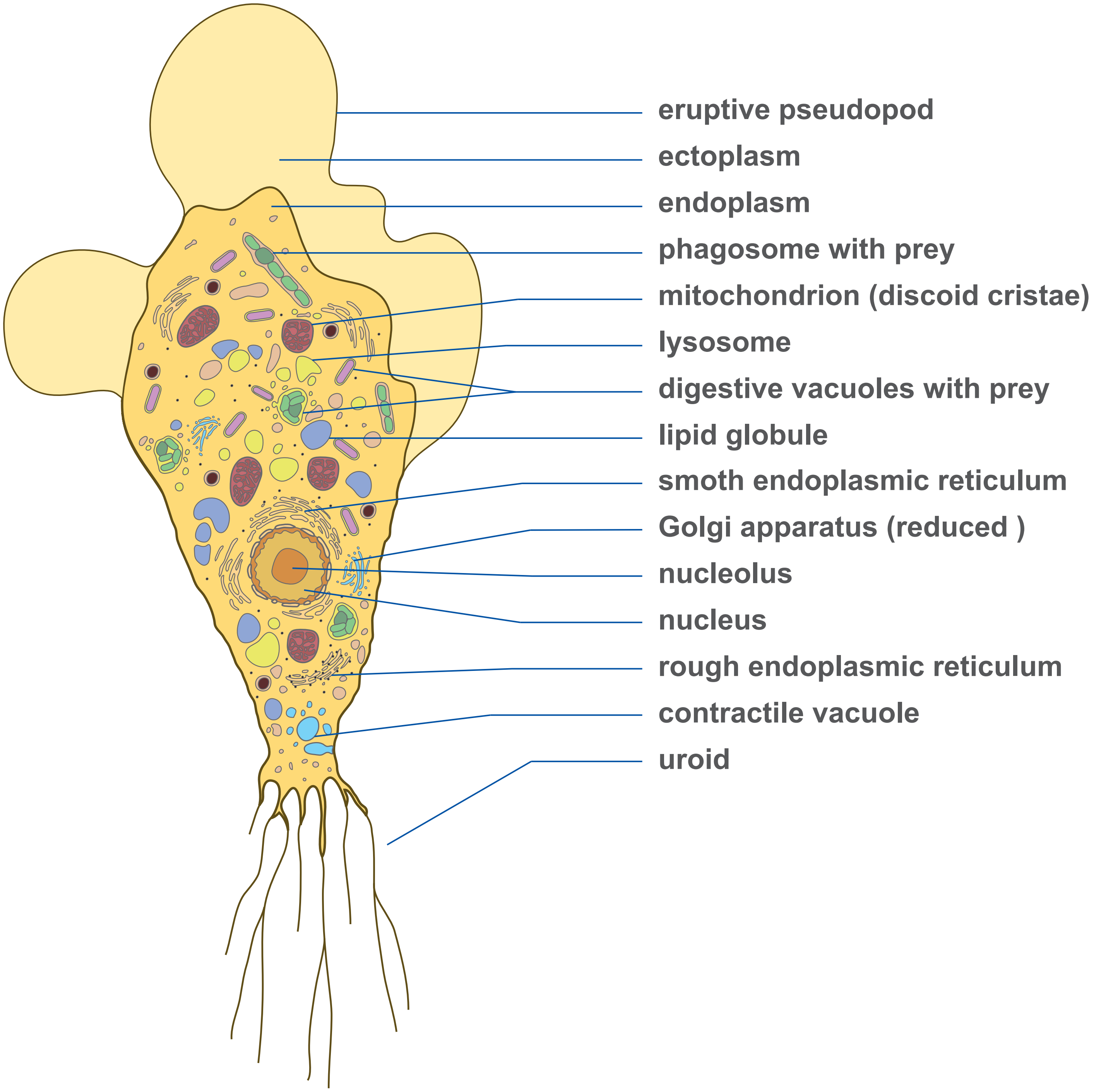

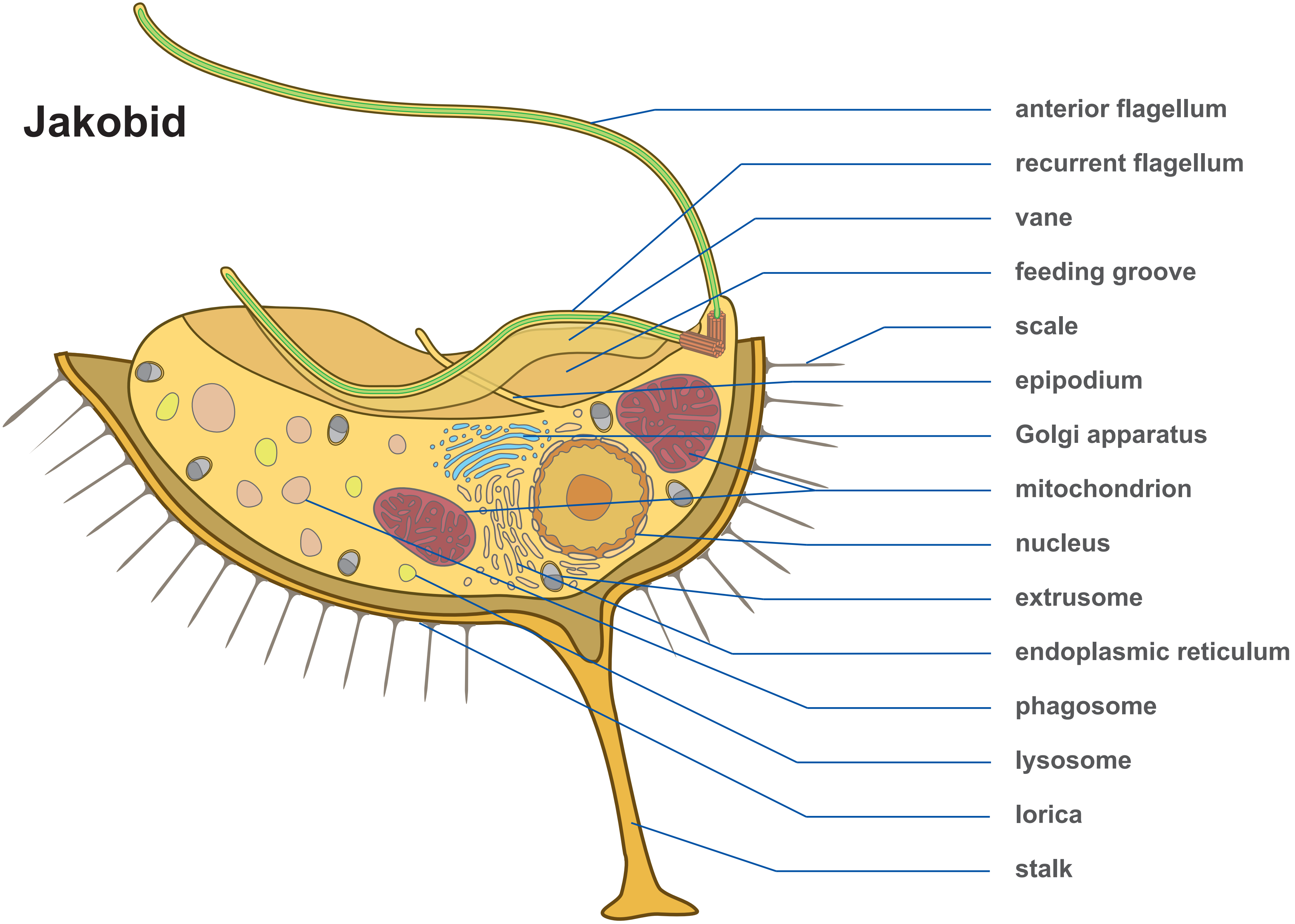
Jakobid
anterior flagellum
recurrent flagellum
vane
feeding groove
scale
epipodium
Golgi apparatus
mitochondrion
nucleus
extrusome
endoplasmic reticulum
phagosome
lysosome
lorica
stalk

Diplomonad

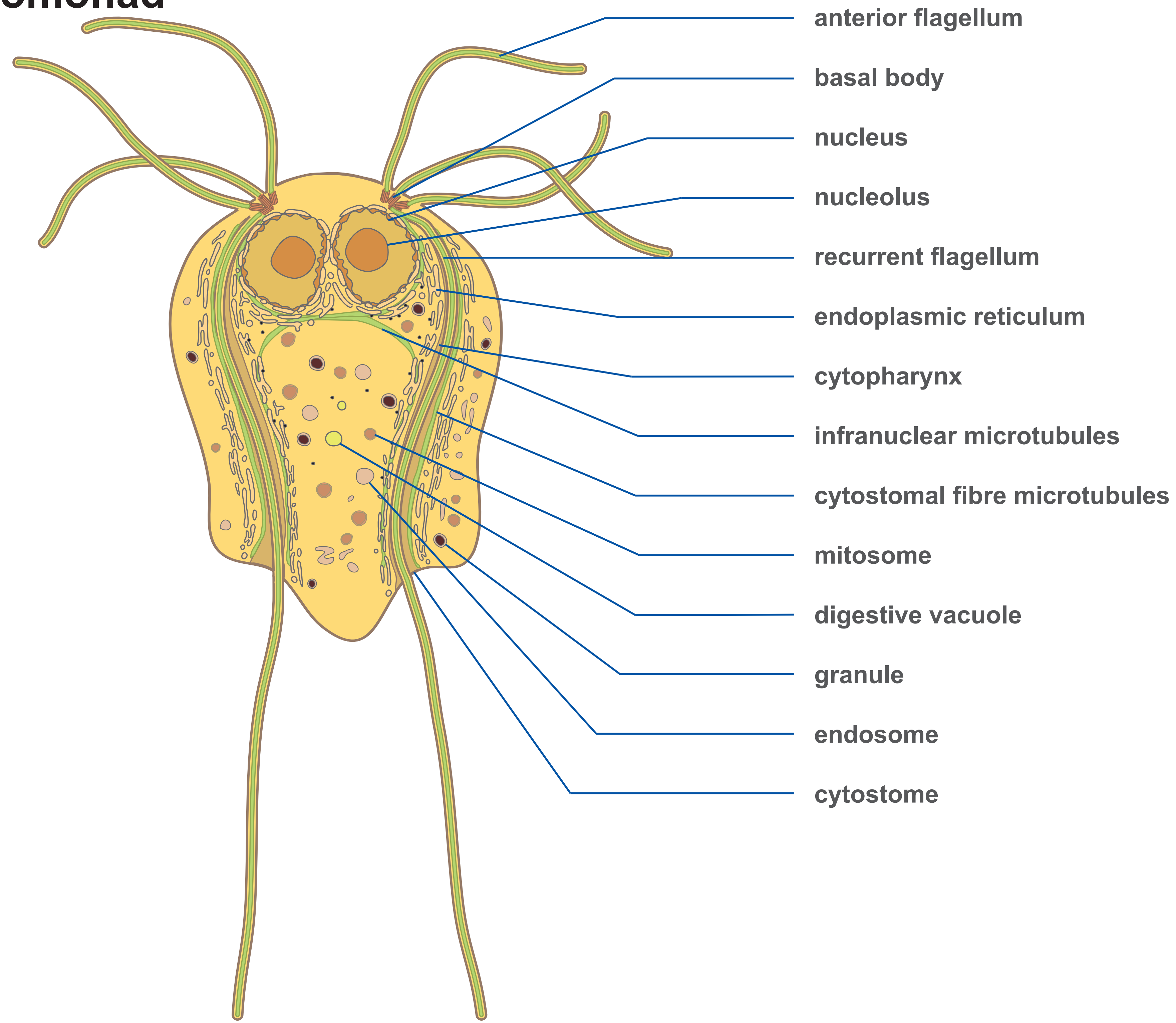
anterior flagellum
basal body
nucleus
nucleolus
recurrent flagellum
endoplasmic reticulum
cytopharynx
infranuclear microtubules
cytostomal fibre microtubules
mitosome
digestive vacuole
granule
endosome
cytostome

Oxymonad

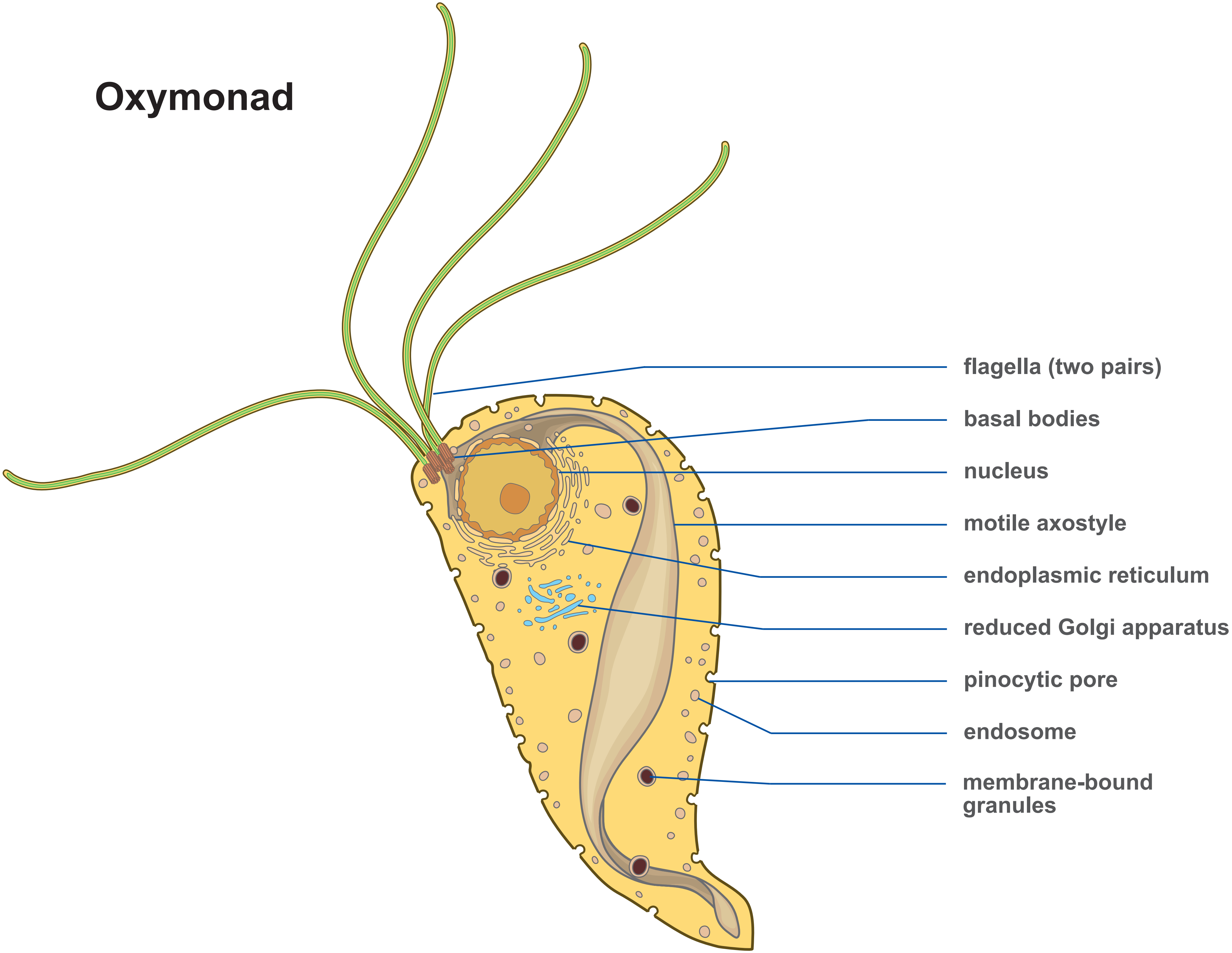
flagella (two pairs)
basal bodies
nucleus
motile axostyle
endoplasmic reticulum
reduced Golgi apparatus
pinocytic pore
endosome
membrane-bound granules

Parabasalian

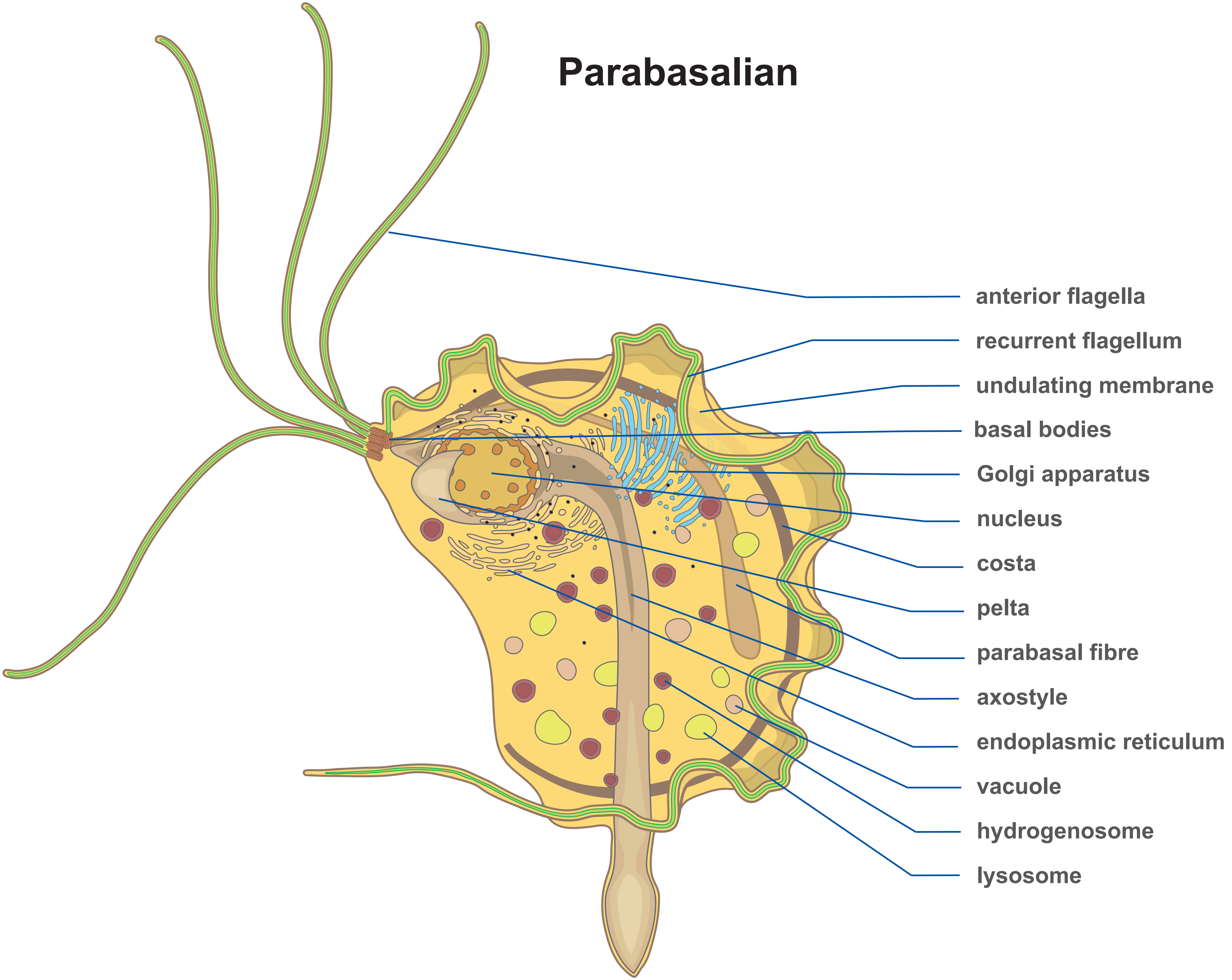

Myxogastrid

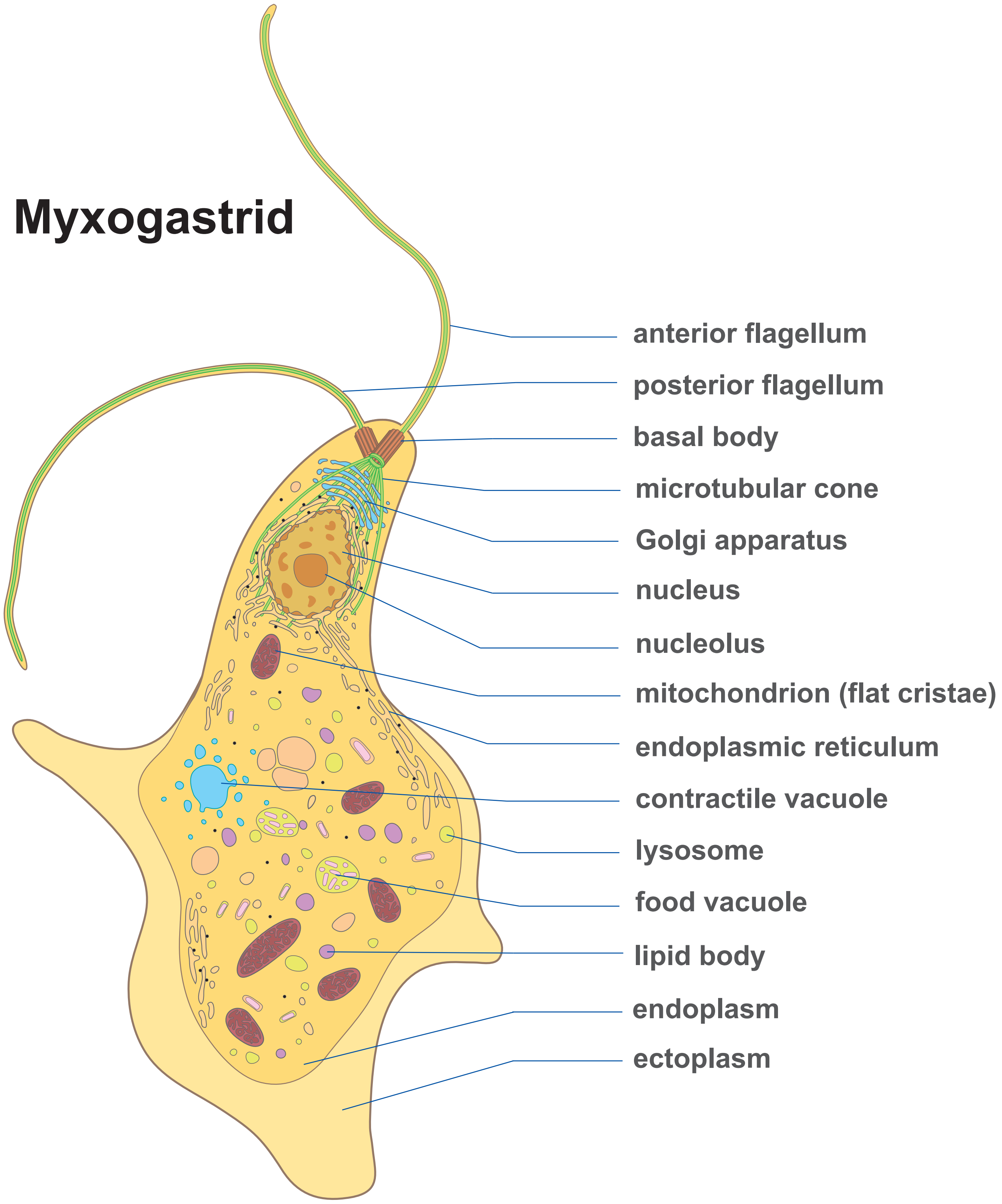

Arcellinid (Tubulinid)

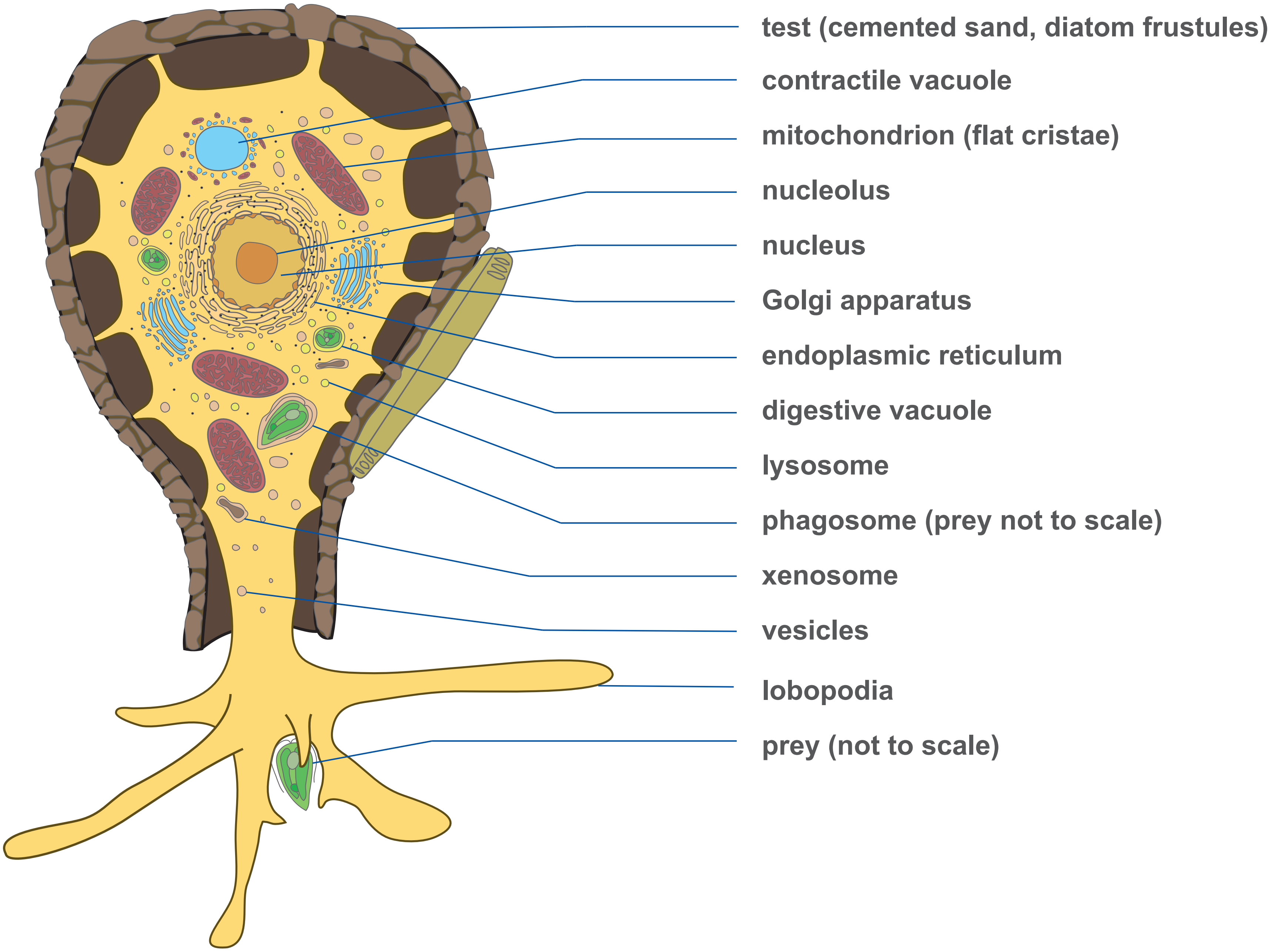

Lobosean (Tubulinid)

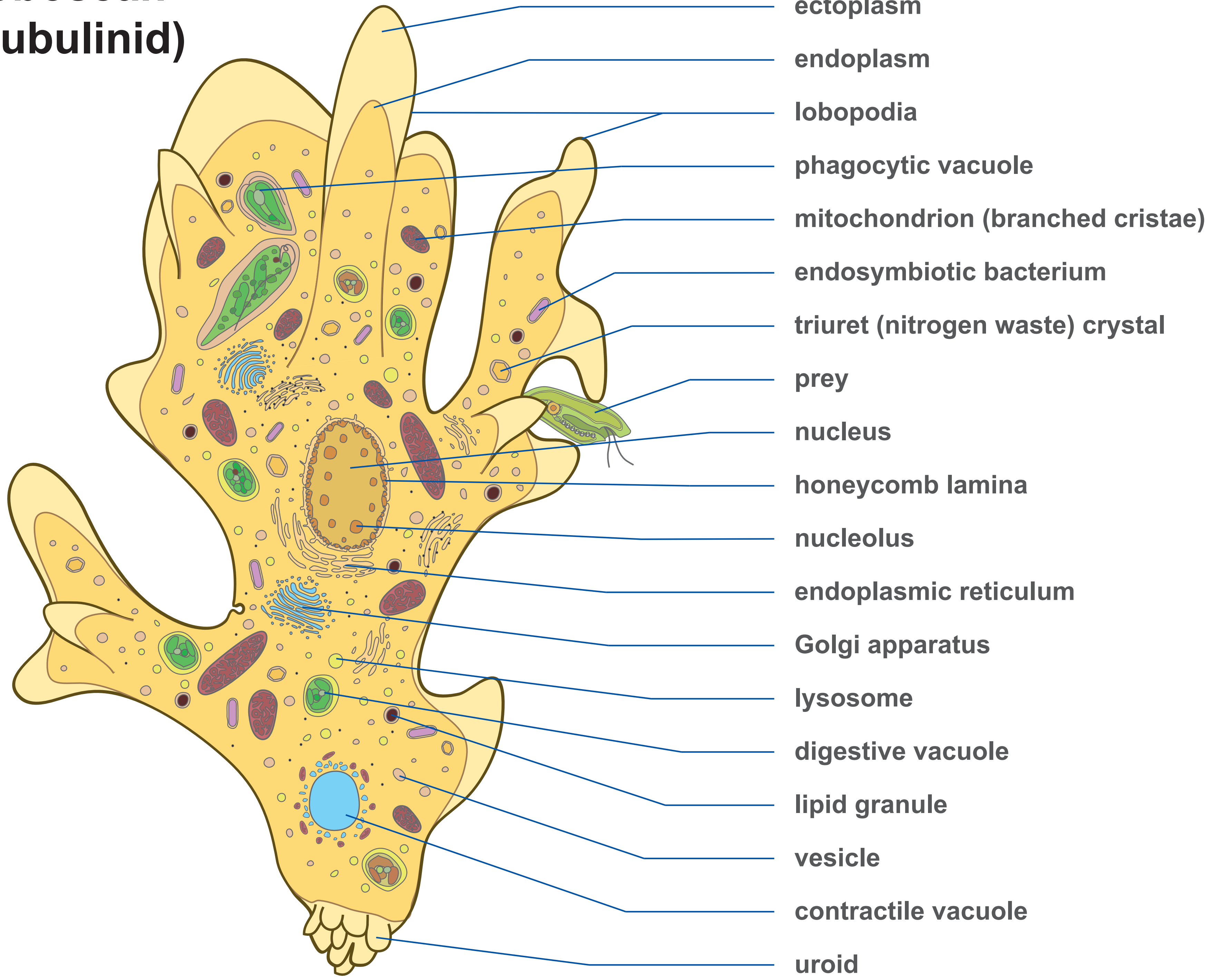

Chytrid

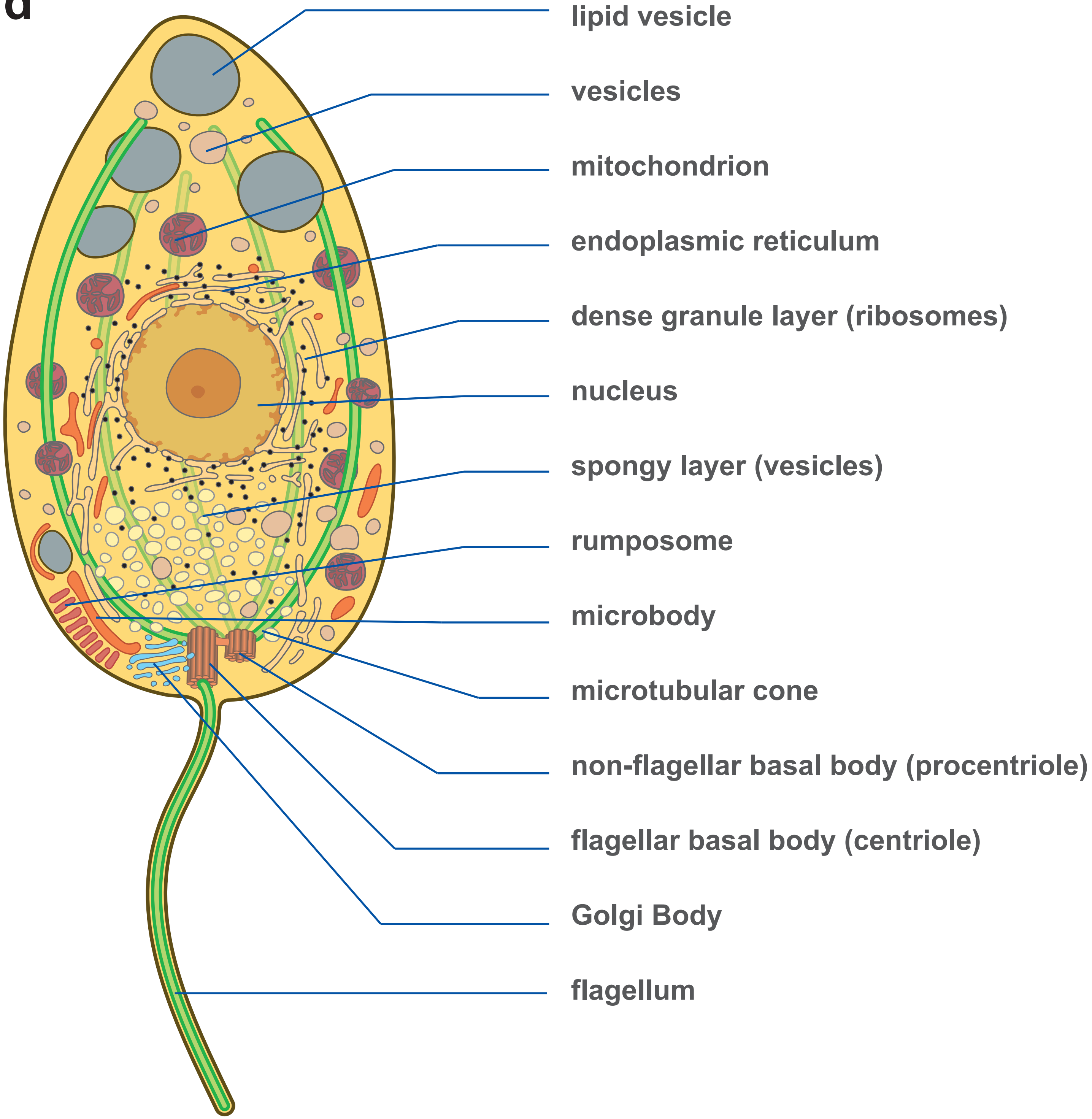

lipid vesicle
vesicles
mitochondrion
endoplasmic reticulum
dense granule layer (ribosomes)
nucleus
spongy layer (vesicles)
rumposome
microbody
microtubular cone
non-flagellar basal body (procentriole)
flagellar basal body (centriole)
Golgi Body
flagellum

Choanoflagellate

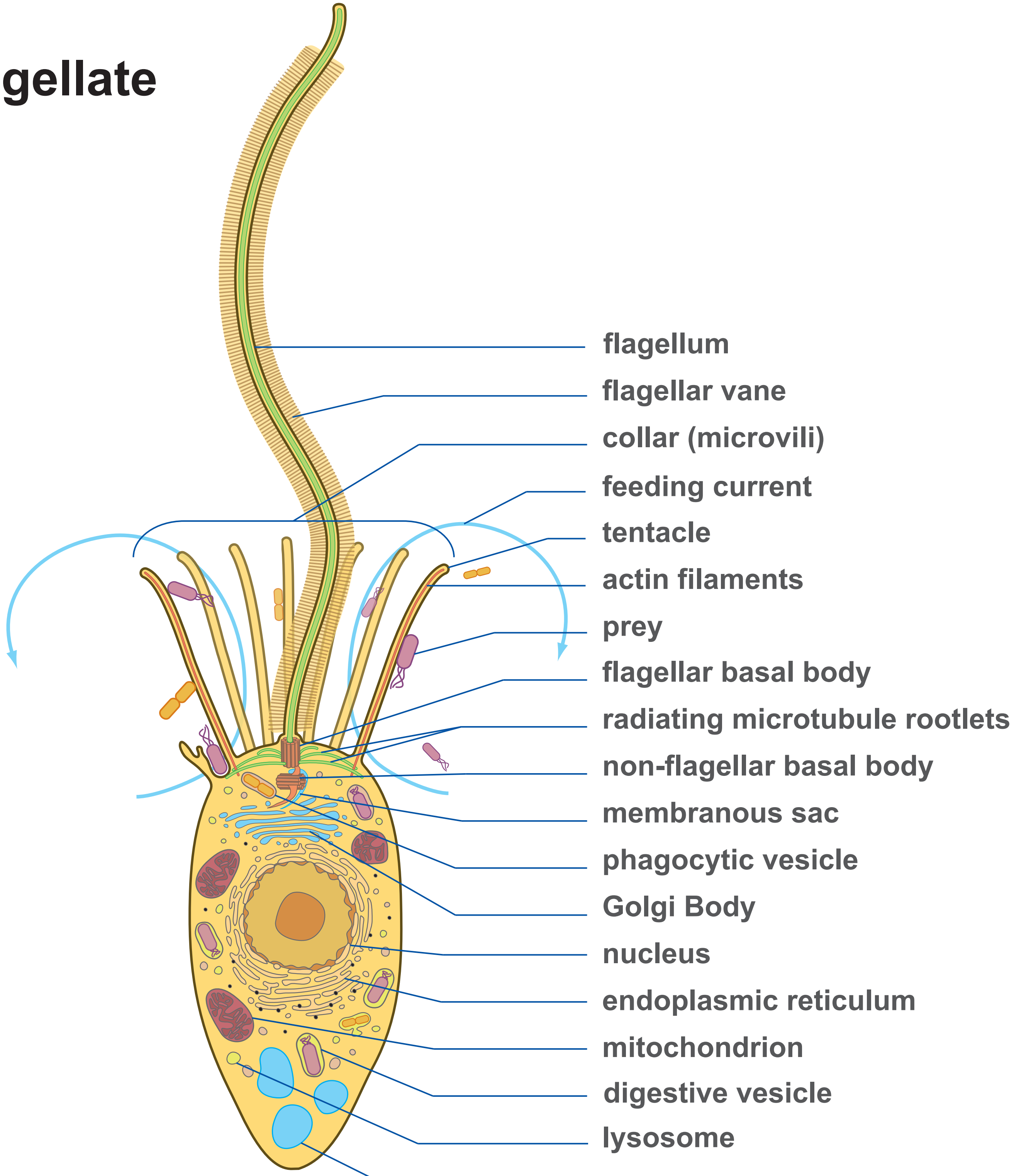

Choanocyte (poriferan feeding cell)

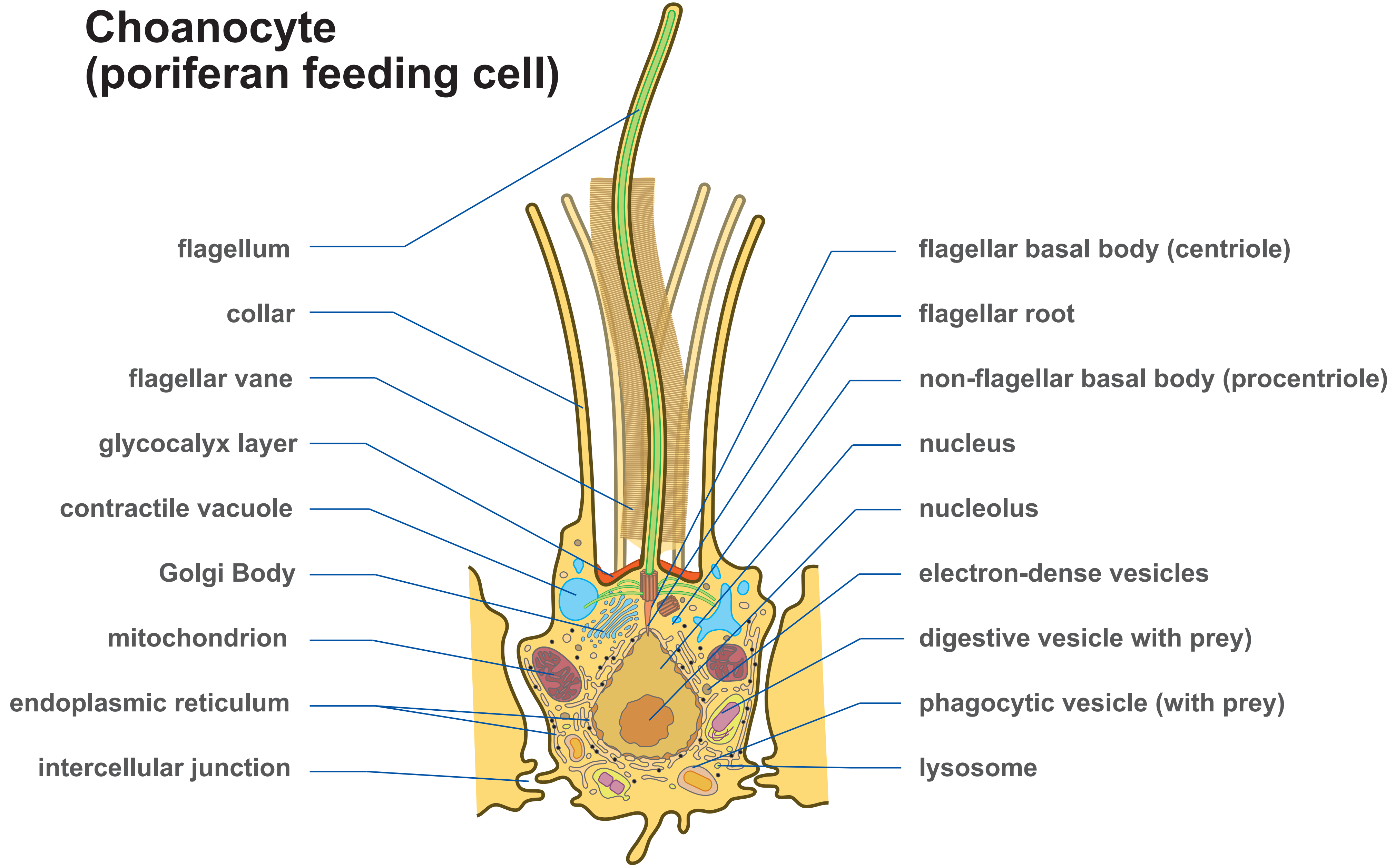

Charophyte

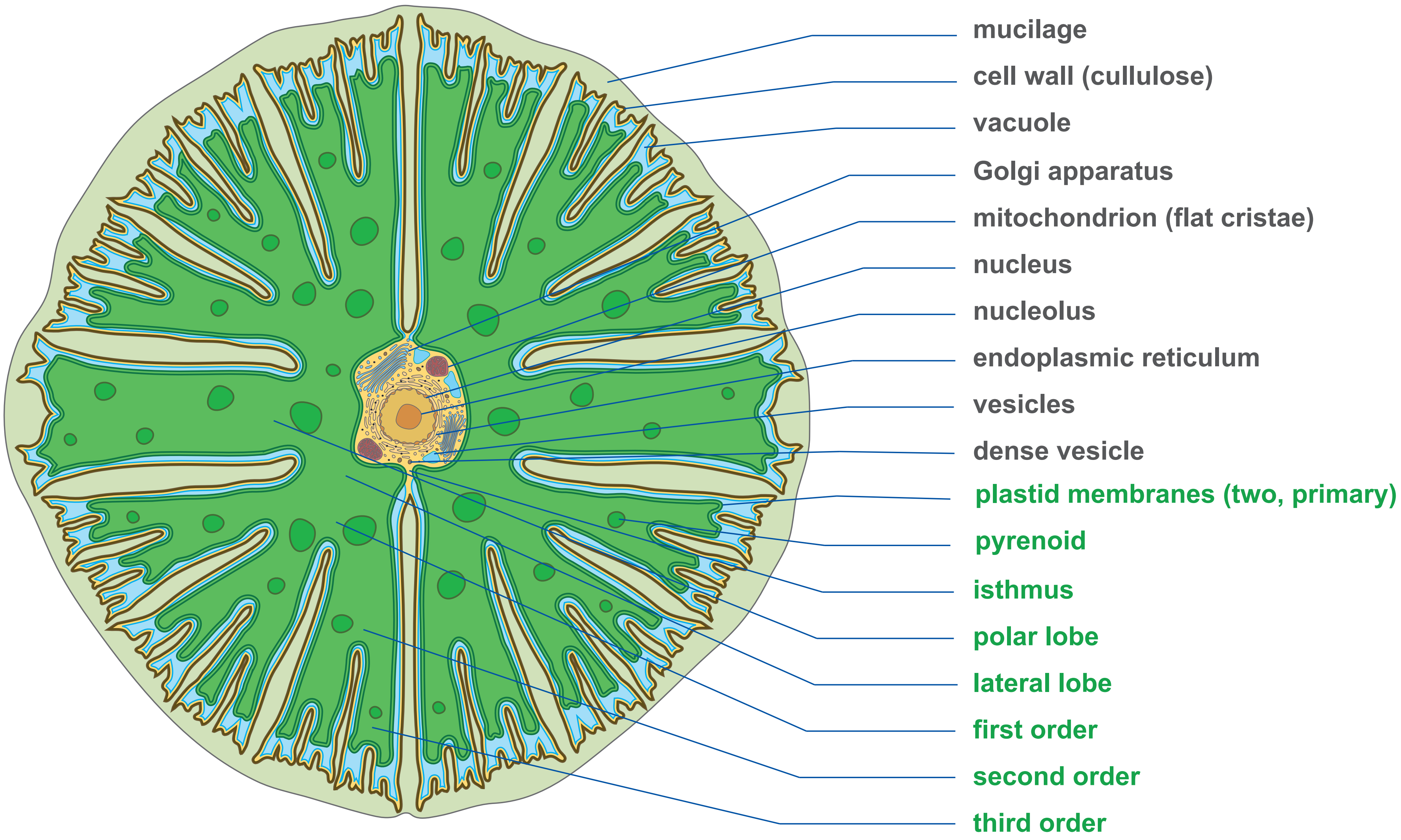

mucilage
cell wall (cullulose)
vacuole
Golgi apparatus
mitochondrion (flat cristae)
nucleus
nucleolus
endoplasmic reticulum
vesicles
dense vesicle
plastid membranes (two, primary)
pyrenoid
isthmus
polar lobe
lateral lobe
first order
second order
third order

Prasinophyte

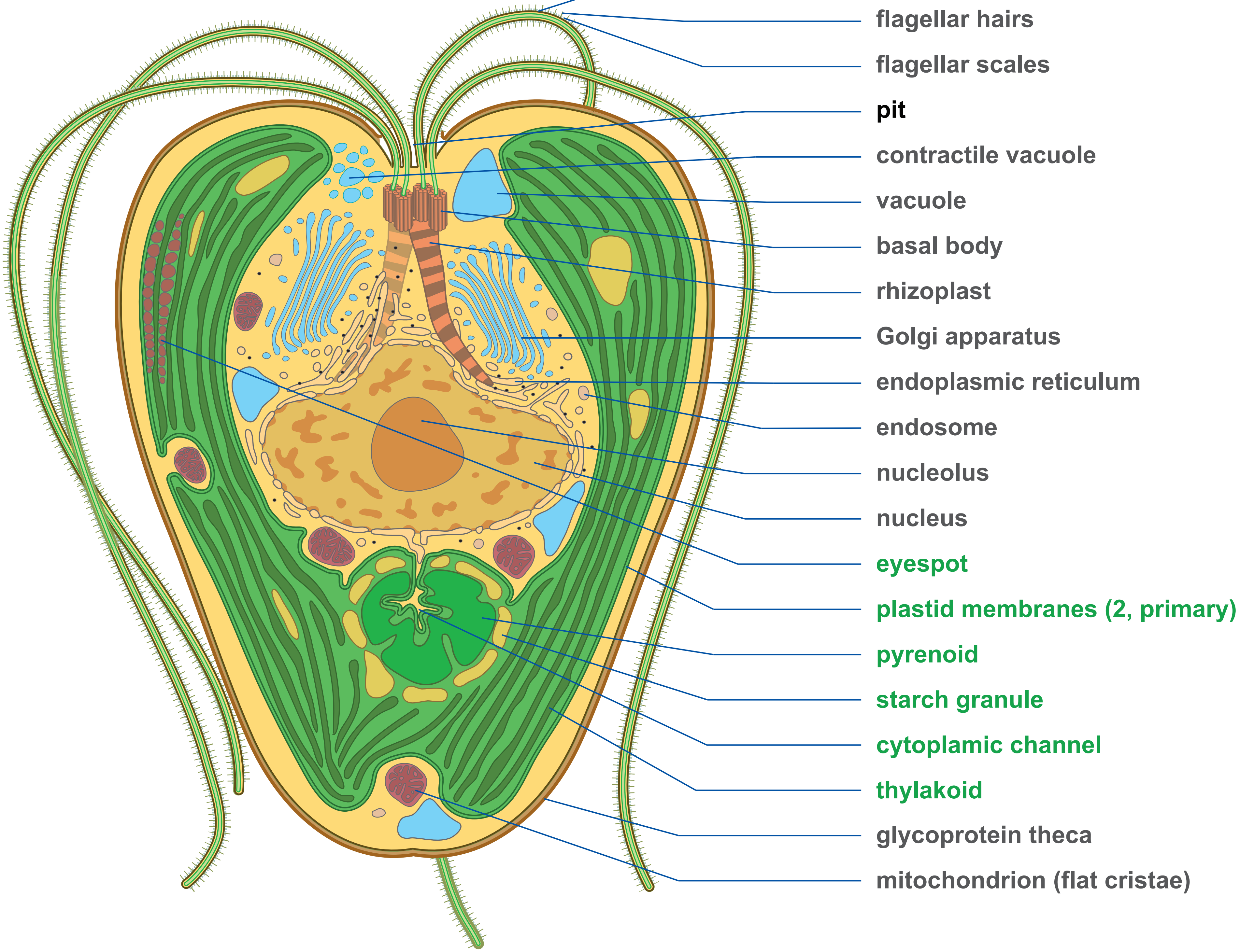

Rhodophyte (red alga)

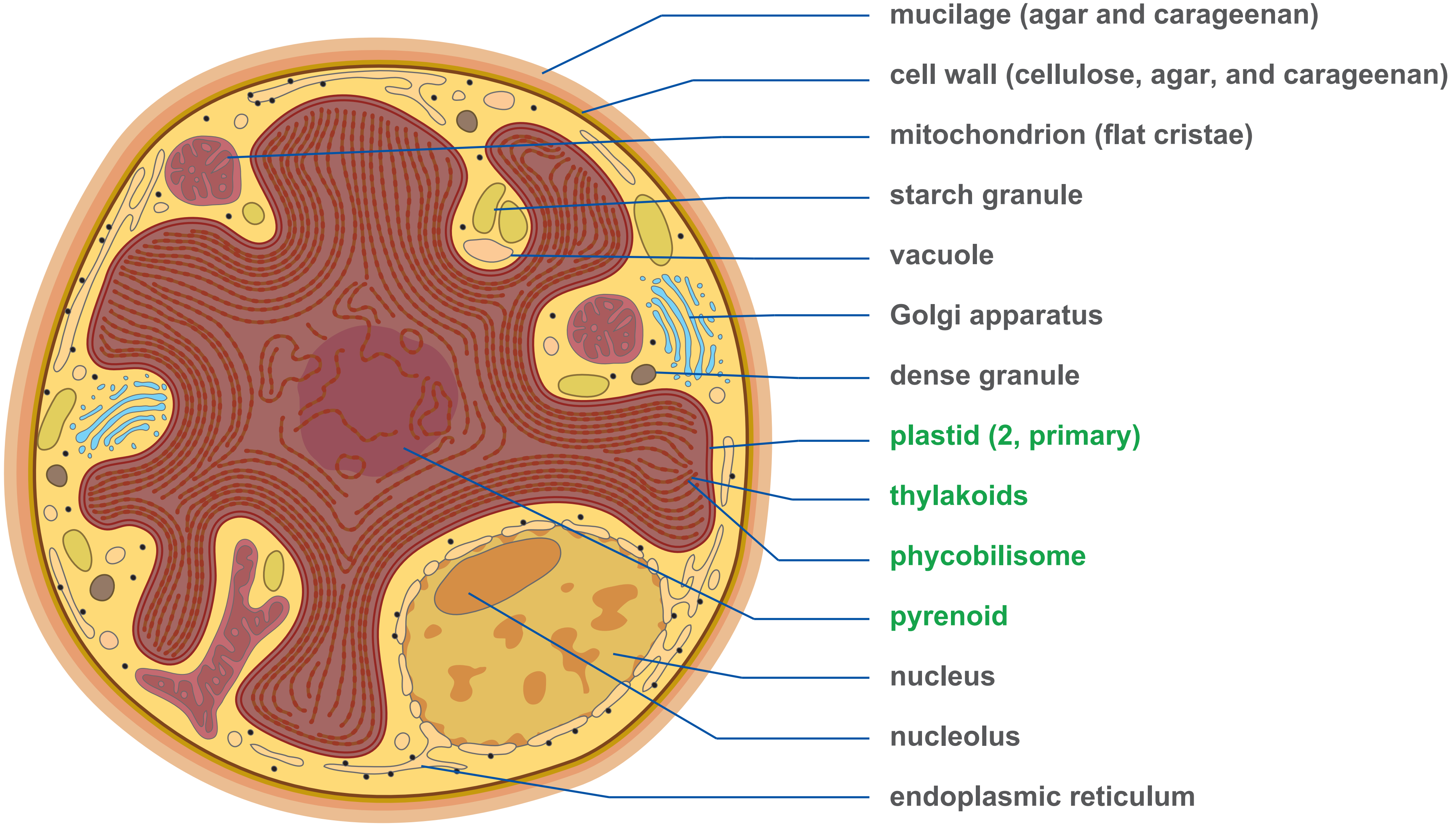

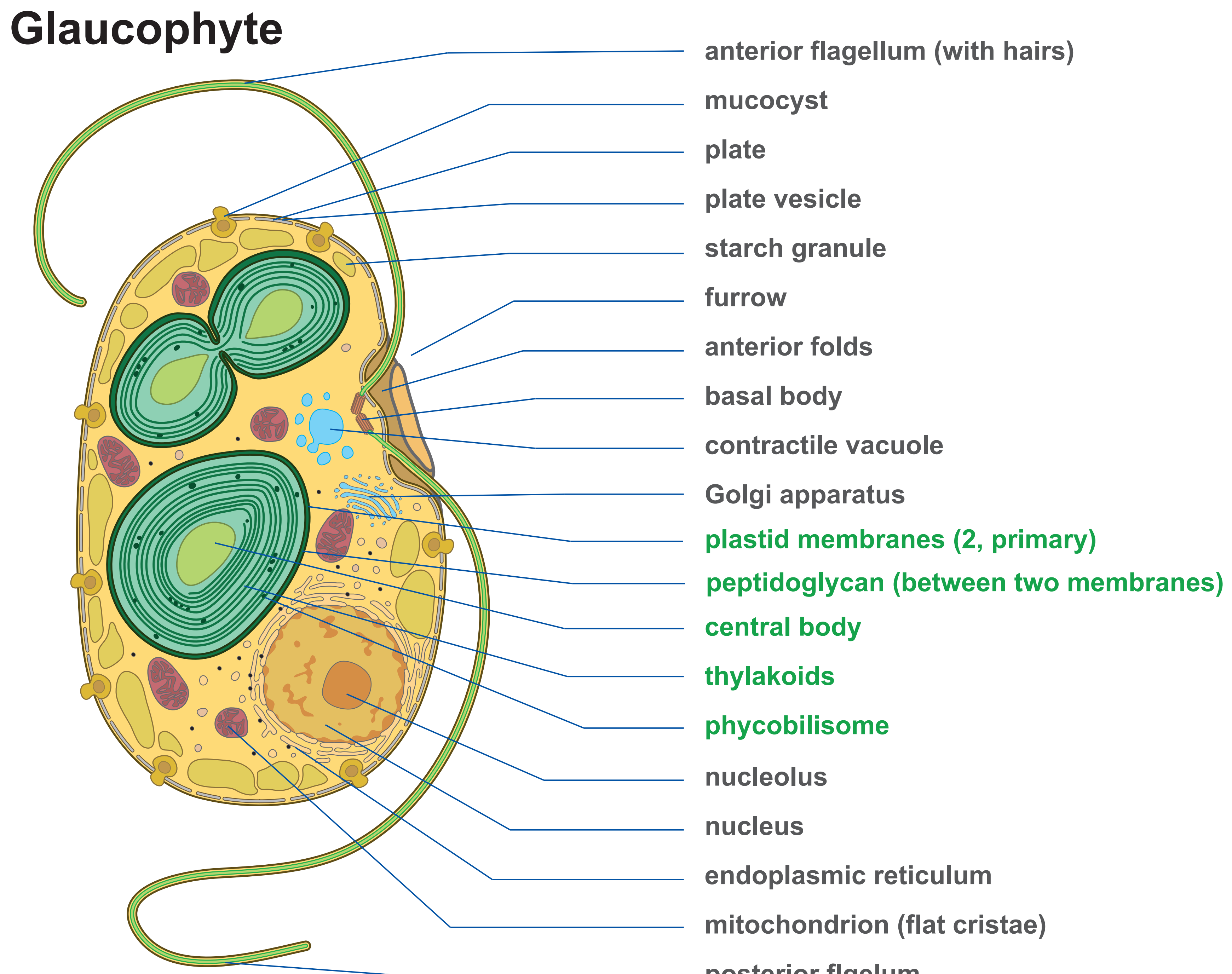
Glaucophyte
anterior flagellum (with hairs)
mucocyst
plate
plate vesicle
starch granule
furrow
anterior folds
basal body
contractile vacuole
Golgi apparatus
plastid membranes (2, primary)
peptidoglycan (between two membranes)
central body
thylakoids
phycobilisome
nucleolus
nucleus
endoplasmic reticulum
mitochondrion (flat cristae)
posterior flgelum

Cryptomonad

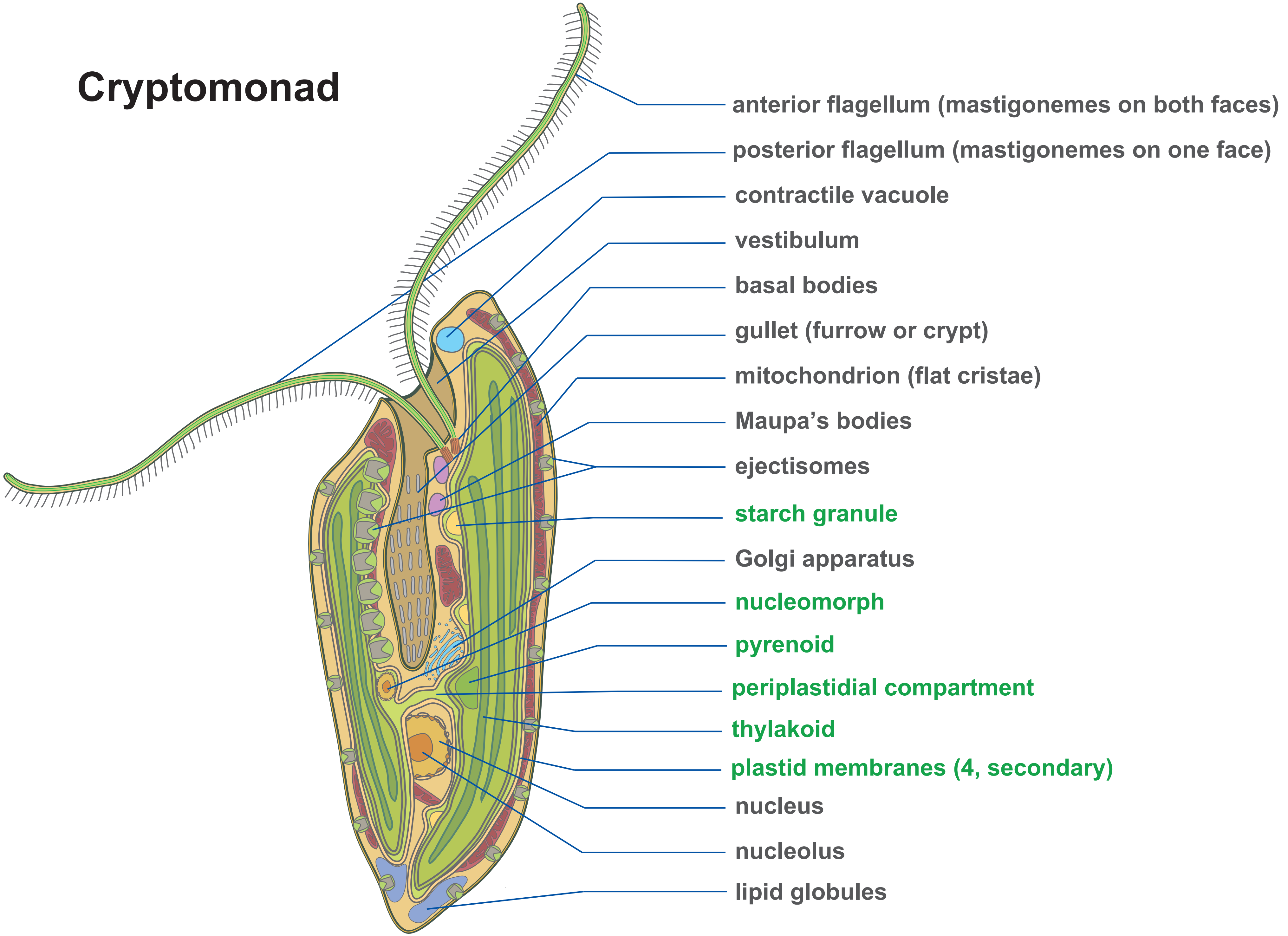

Centrohelid

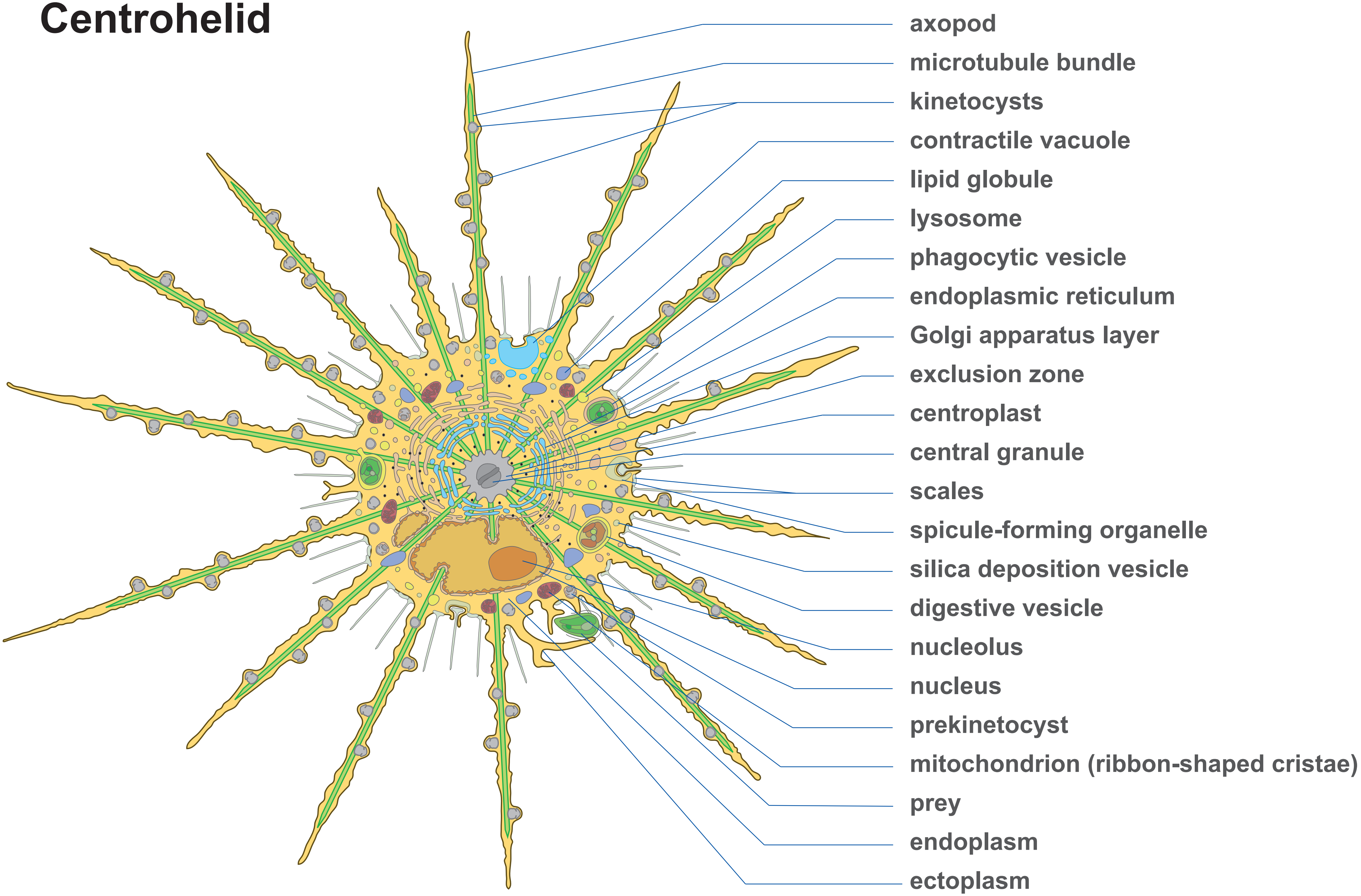

Haptophyte

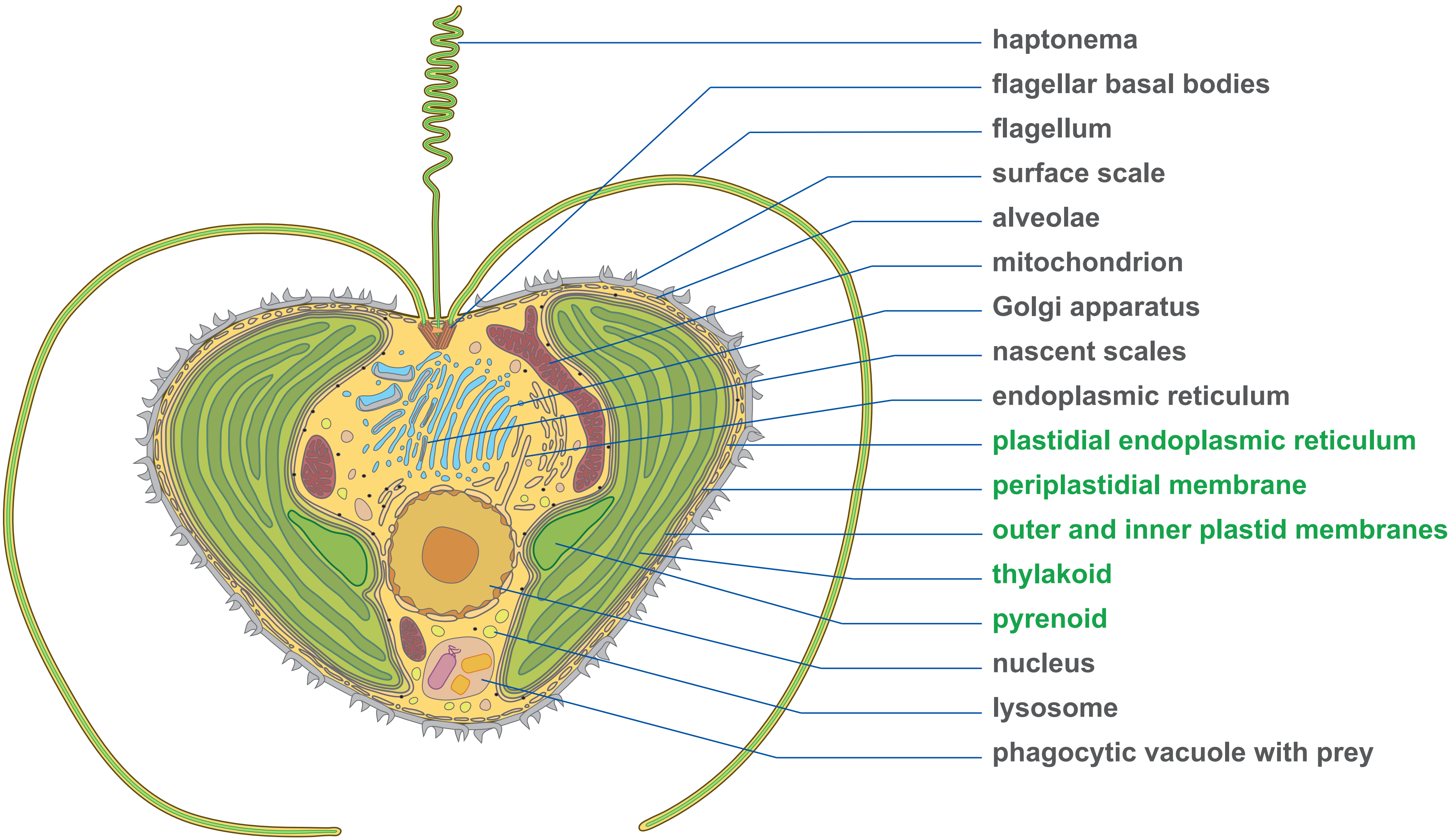
haptonema
flagellar basal bodies
flagellum
surface scale
alveolae
mitochondrion
Golgi apparatus
nascent scales
endoplasmic reticulum
plastidial endoplasmic reticulum
periplastidial membrane
outer and inner plastid membranes
thylakoid
pyrenoid
nucleus
lysosome
phagocytic vacuole with prey

Foraminiferan

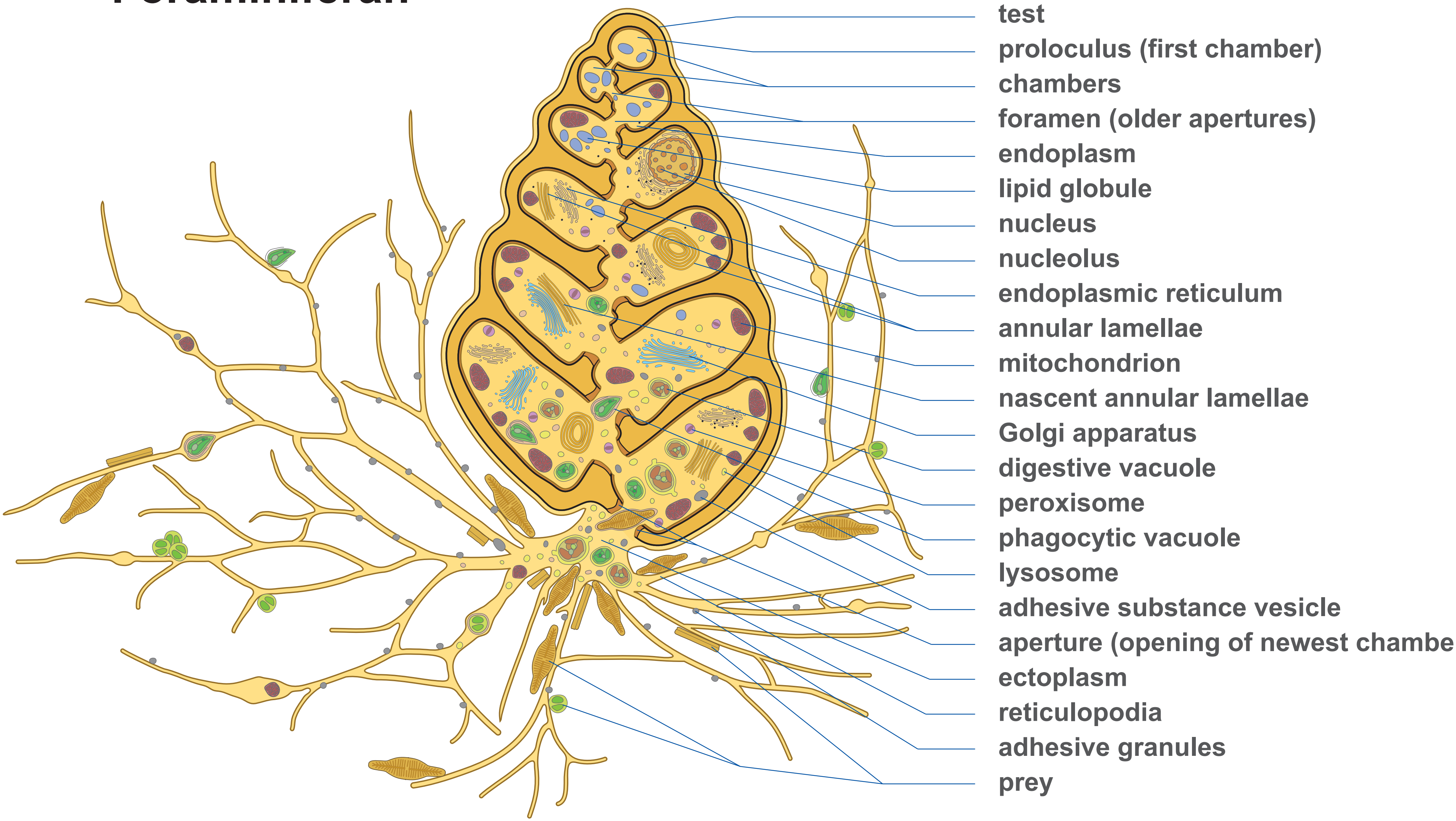

Polycystine

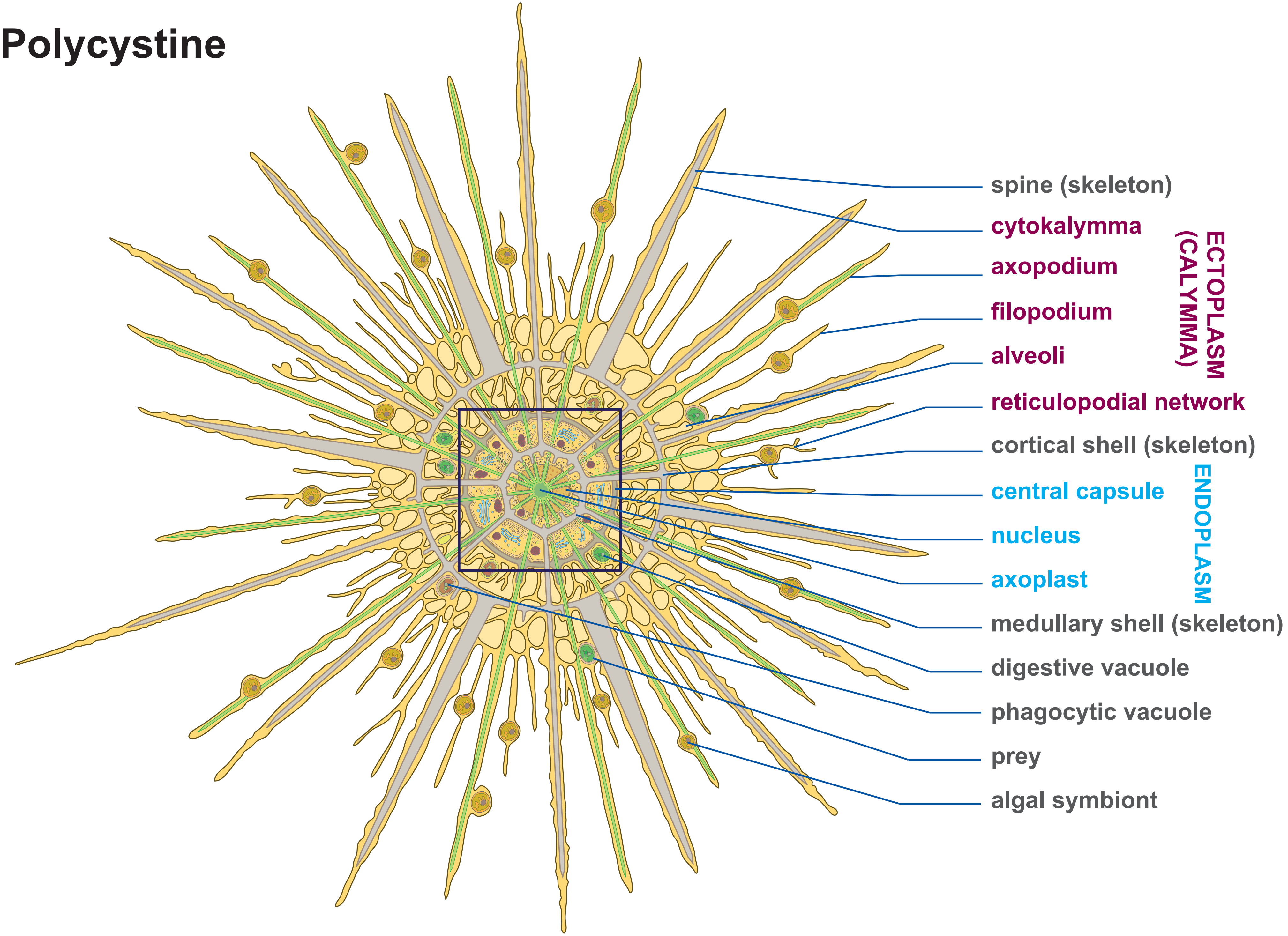

Polycystine (Intracapsulum)

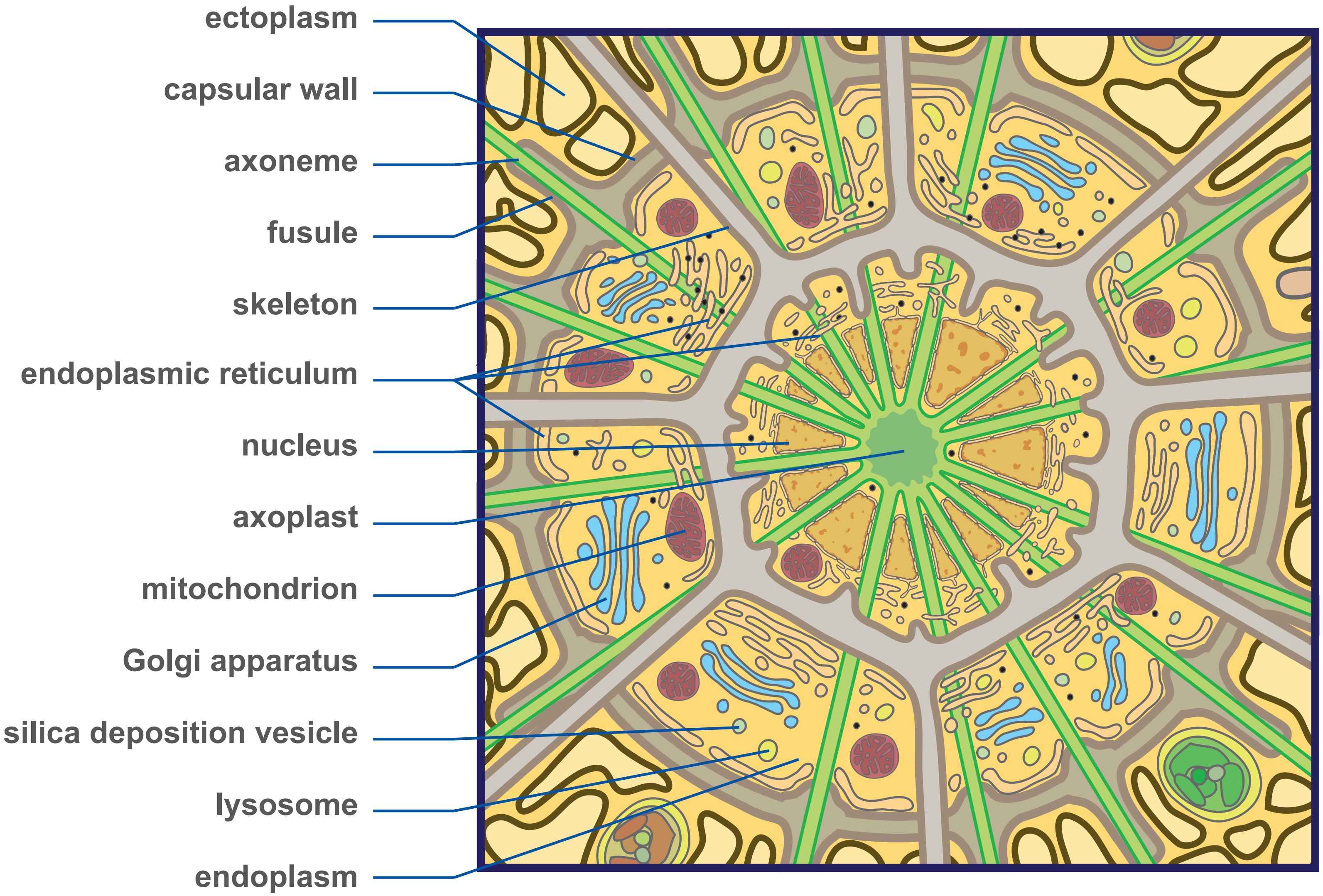

Cercomonad

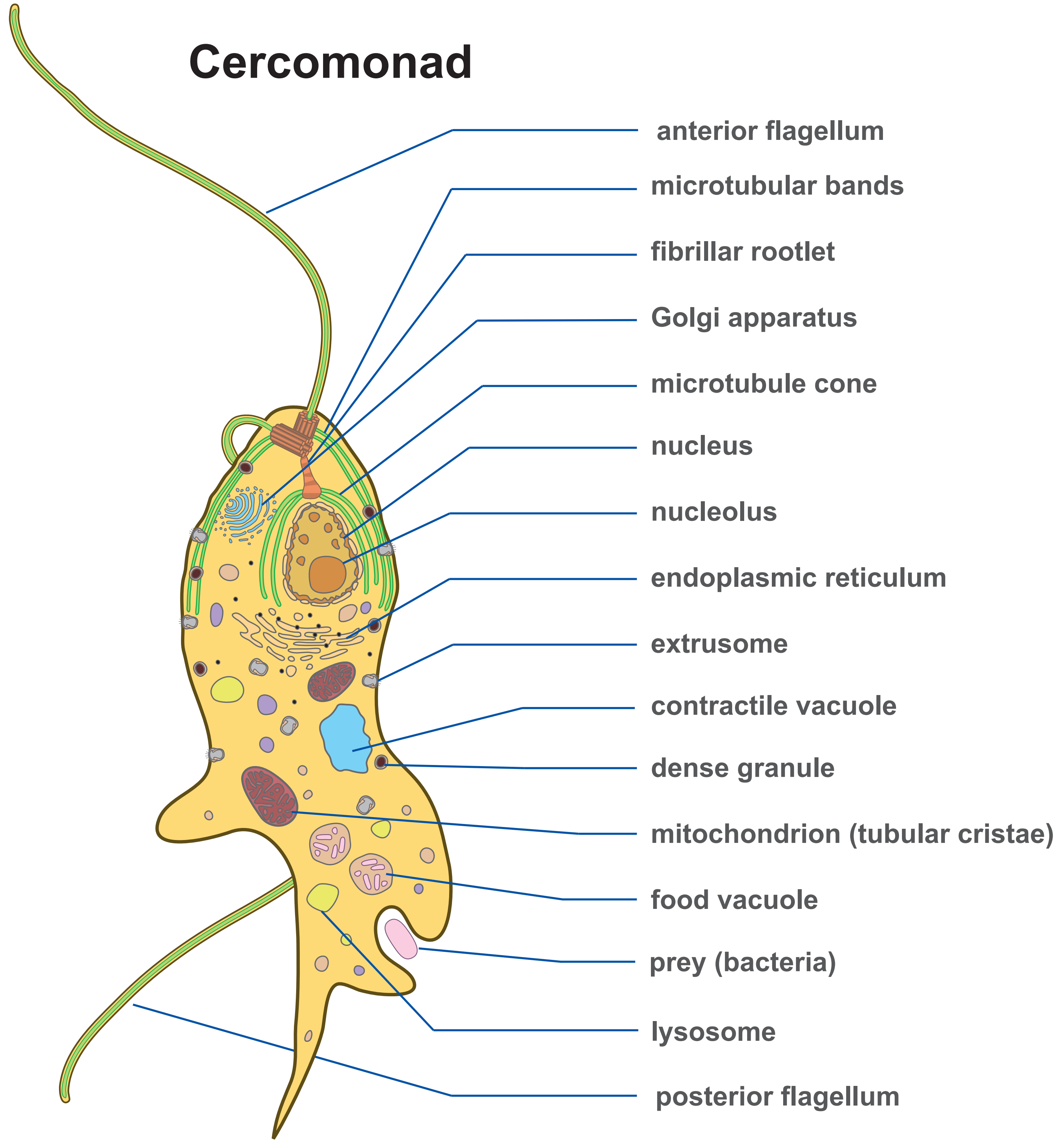
anterior flagellum
microtubular bands
fibrillar rootlet
Golgi apparatus
microtubule cone
nucleus
nucleolus
endoplasmic reticulum
extrusome
contractile vacuole
dense granule
mitochondrion (tubular cristae)
food vacuole
prey (bacteria)
lysosome
posterior flagellum

Chlorarachniophyte

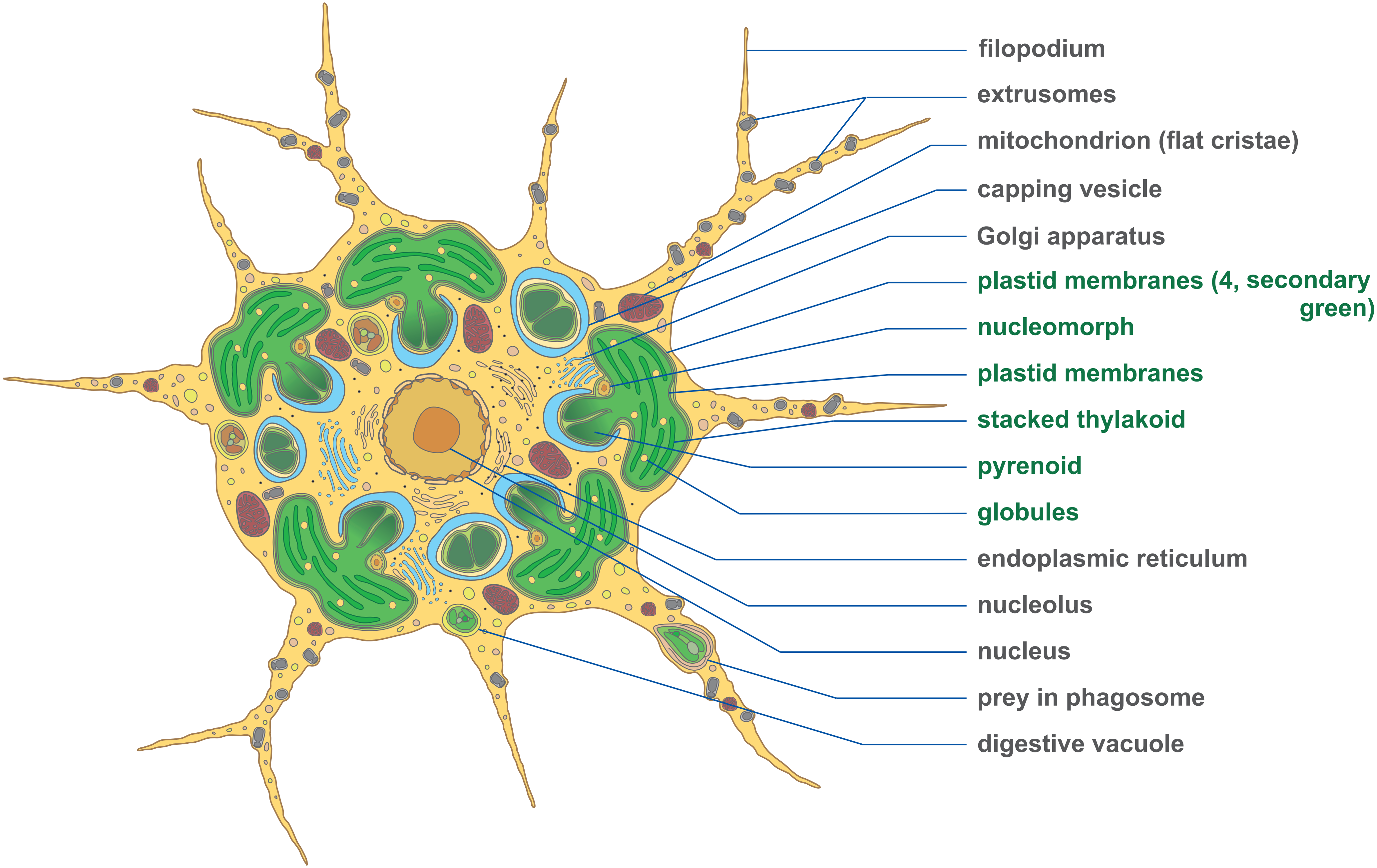

Euglyphid

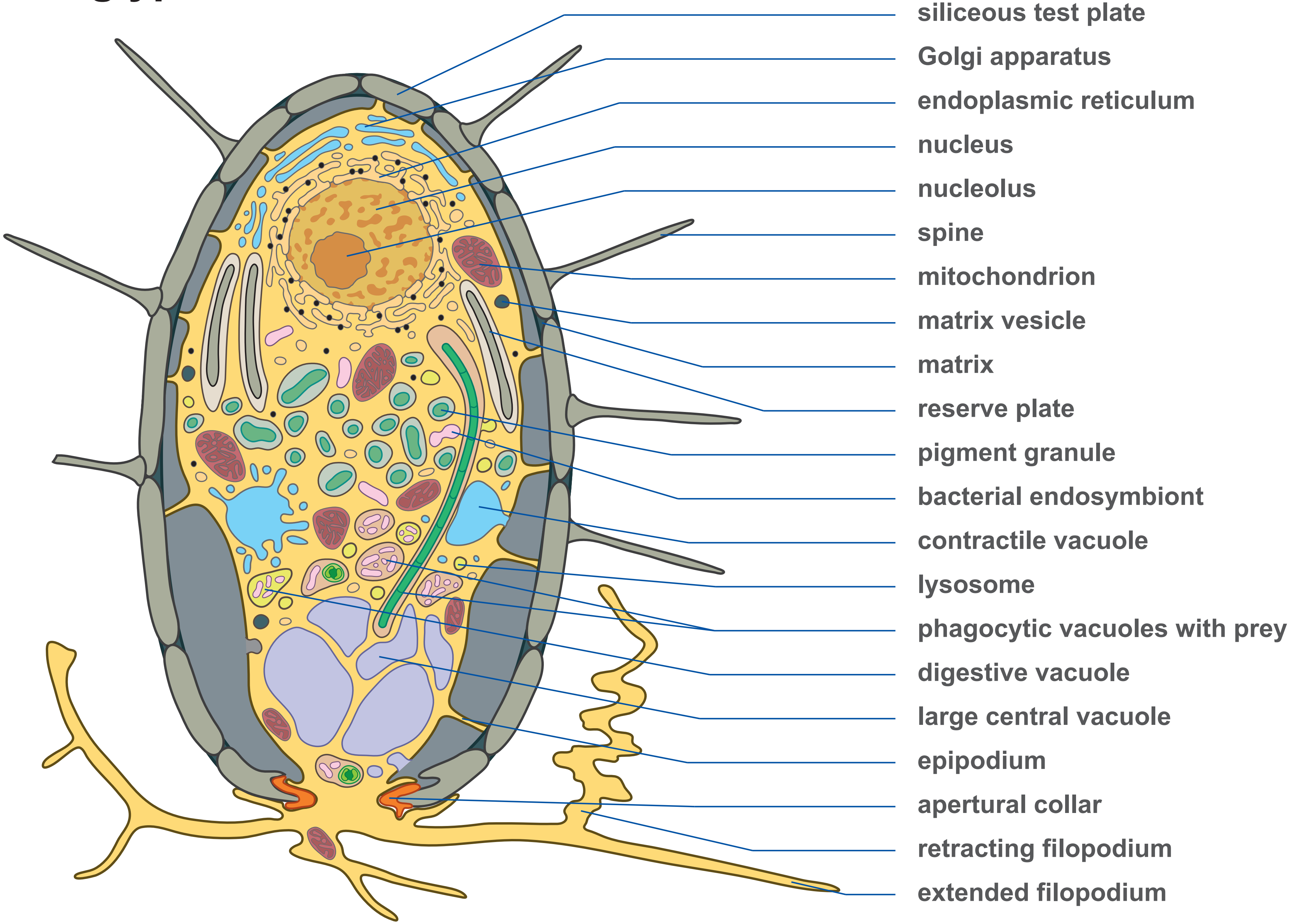
siliceous test plate
Golgi apparatus
endoplasmic reticulum
nucleus
nucleolus
spine
mitochondrion
matrix vesicle
matrix
reserve plate
pigment granule
bacterial endosymbiont
contractile vacuole
lysosome
phagocytic vacuoles with prey
digestive vacuole
large central vacuole
epipodium
apertural collar
retracting filopodium
extended filopodium

Ciliate

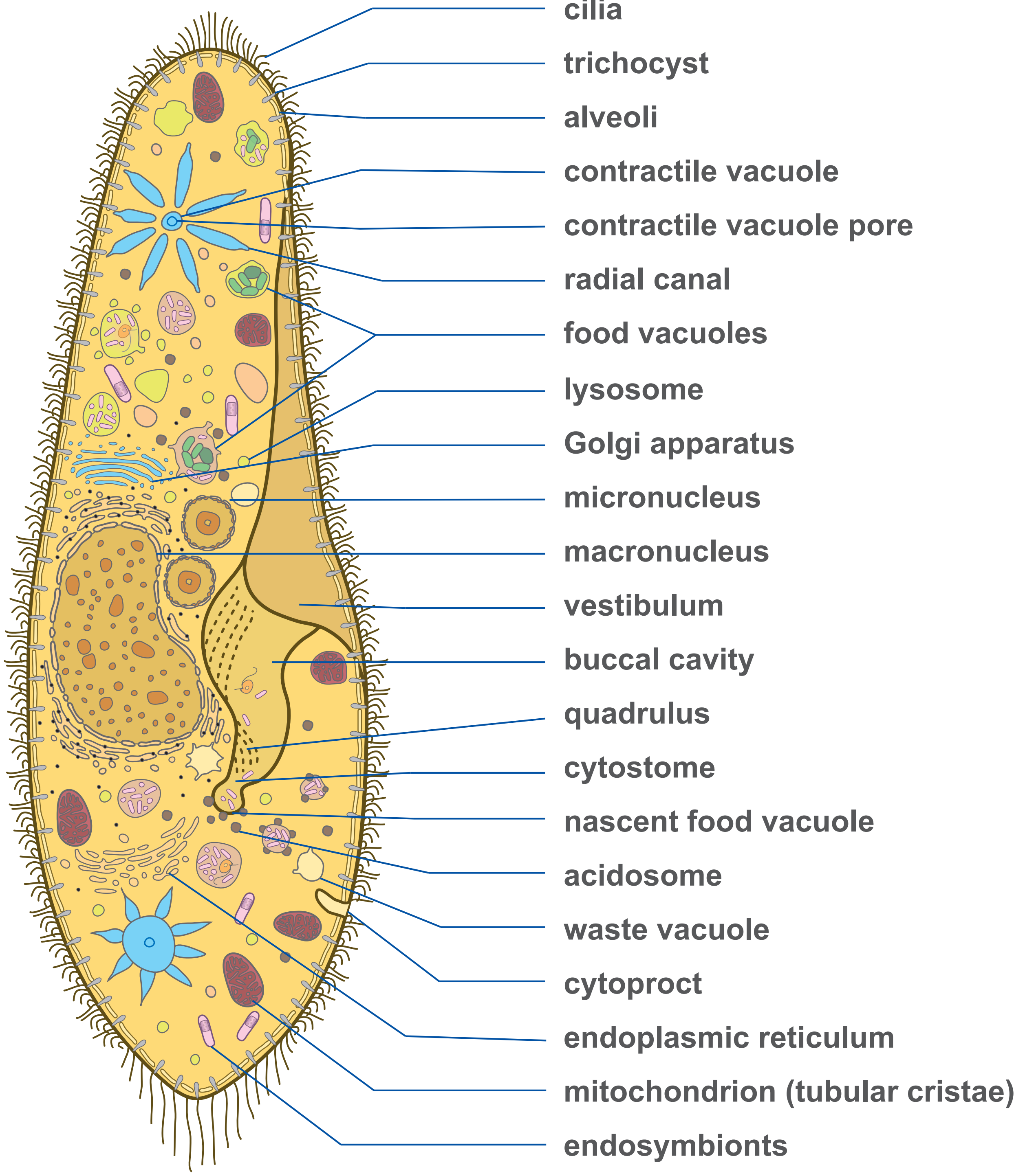
cilia
trichocyst
alveoli
contractile vacuole
contractile vacuole pore
radial canal
food vacuoles
lysosome
Golgi apparatus
micronucleus
macronucleus
vestibulum
buccal cavity
quadrulus
cytostome
nascent food vacuole
acidosome
waste vacuole
cytoproct
endoplasmic reticulum
mitochondrion (tubular cristae)
endosymbionts

Apicomplexan

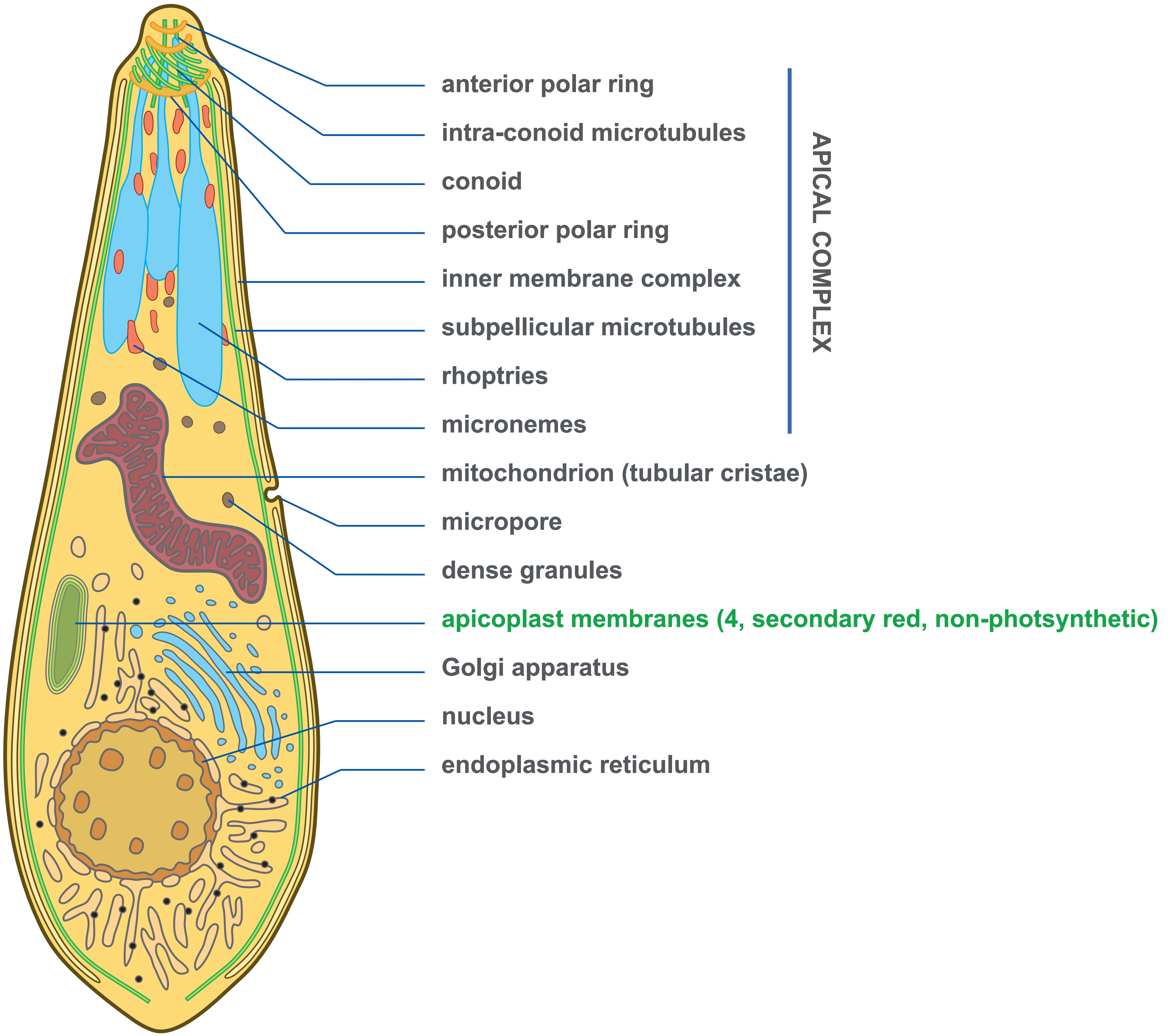

Dinoflagellate

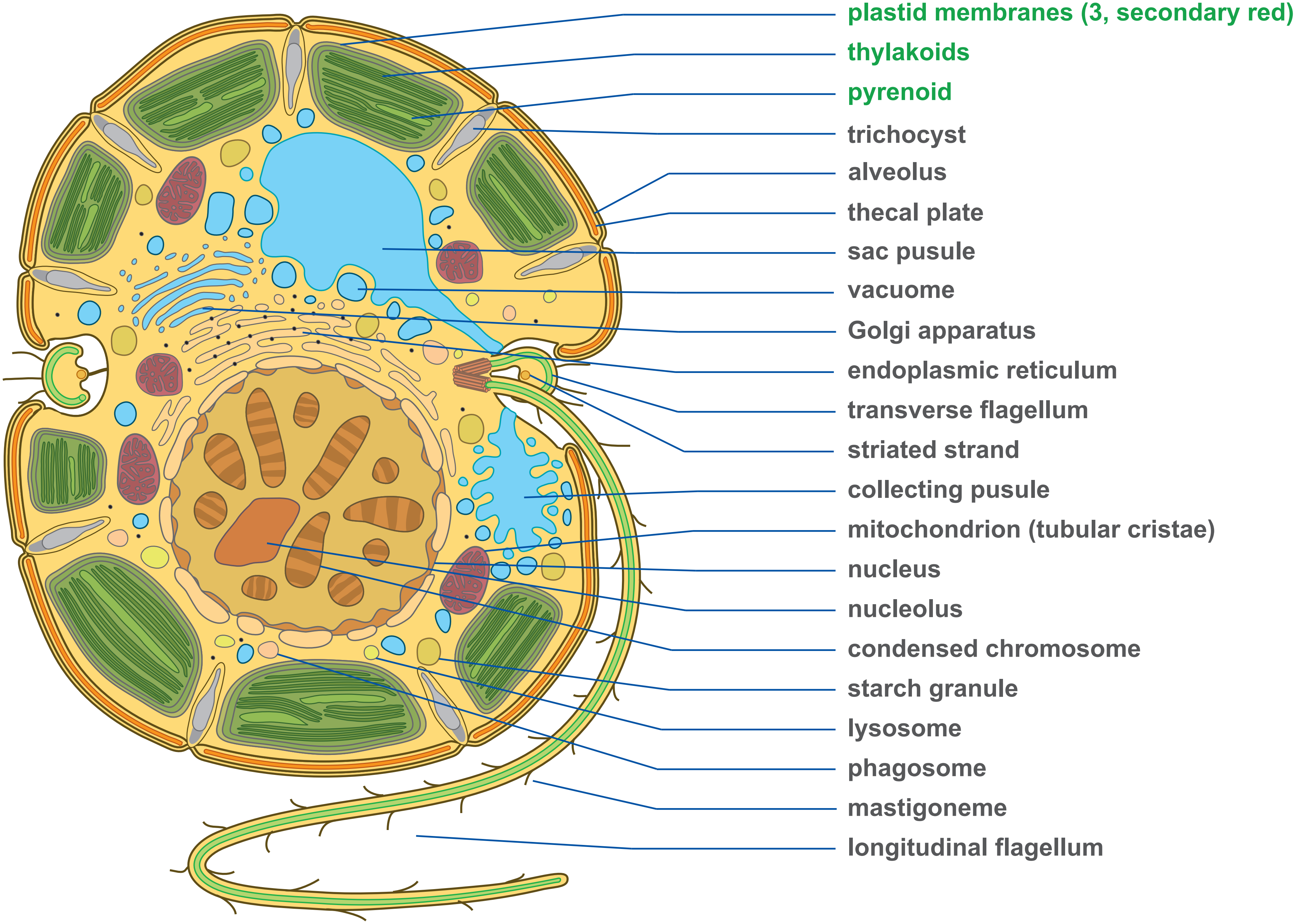

Bicosoecid

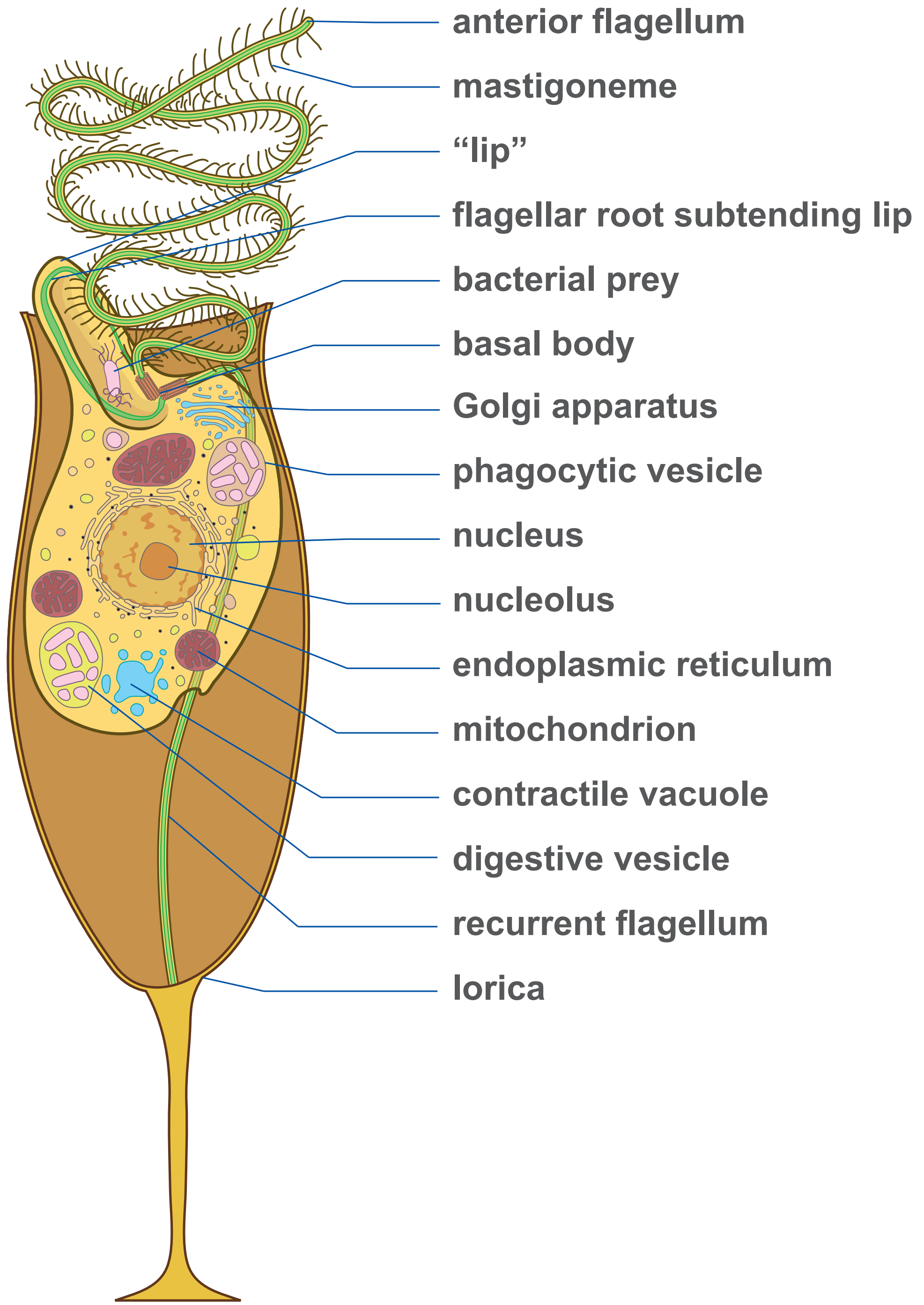
anterior flagellum
mastigoneme
“lip”
flagellar root subtending lip
bacterial prey
basal body
Golgi apparatus
phagocytic vesicle
nucleus
nucleolus
endoplasmic reticulum
mitochondrion
contractile vacuole
digestive vesicle
recurrent flagellum
lorica

Oomycete

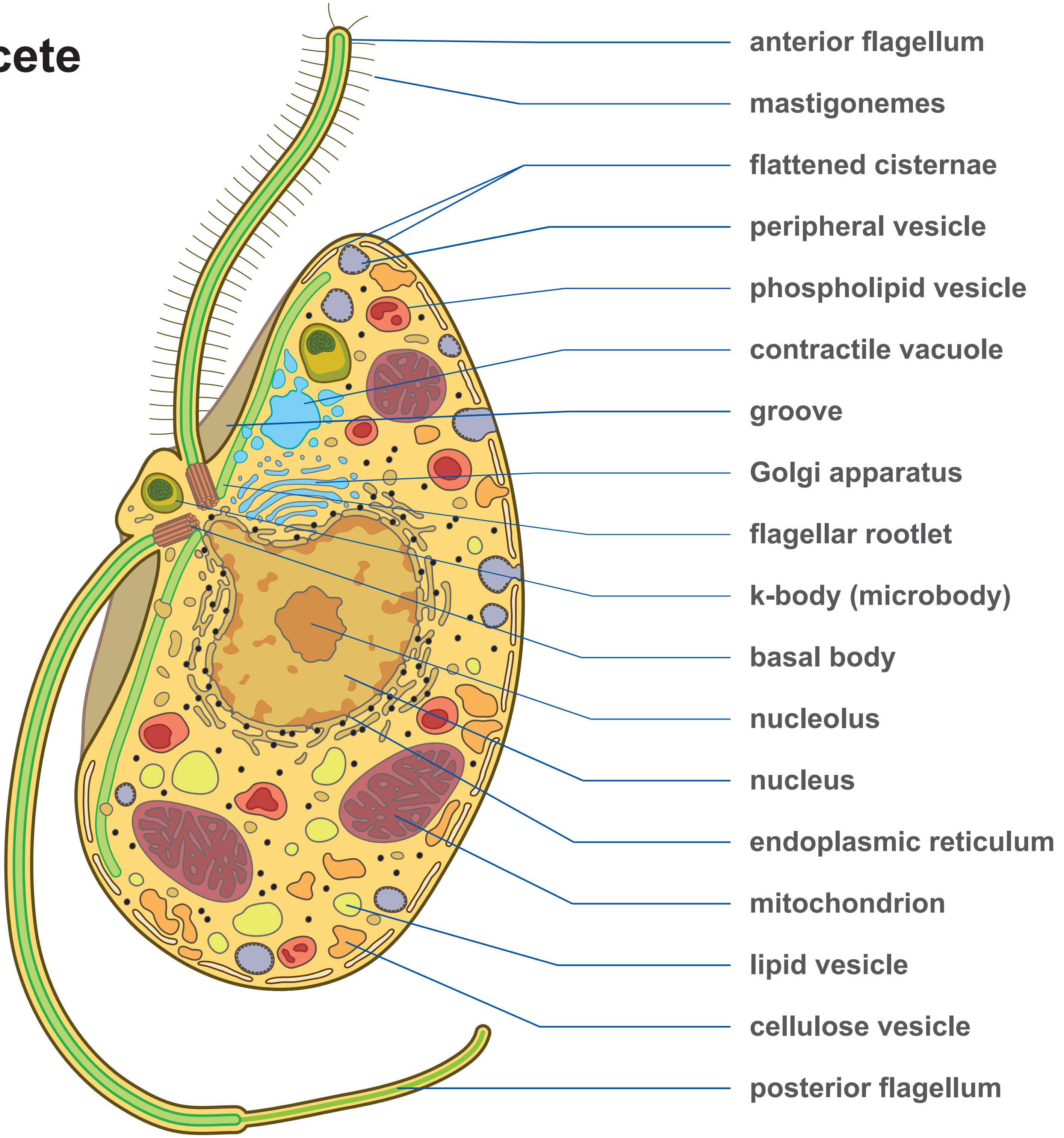
anterior flagellum
mastigonemes
flattened cisternae
peripheral vesicle
phospholipid vesicle
contractile vacuole
groove
Golgi apparatus
flagellar rootlet
k-body (microbody)
basal body
nucleolus
nucleus
endoplasmic reticulum
mitochondrion
lipid vesicle
cellulose vesicle
posterior flagellum

Oomycete invasion

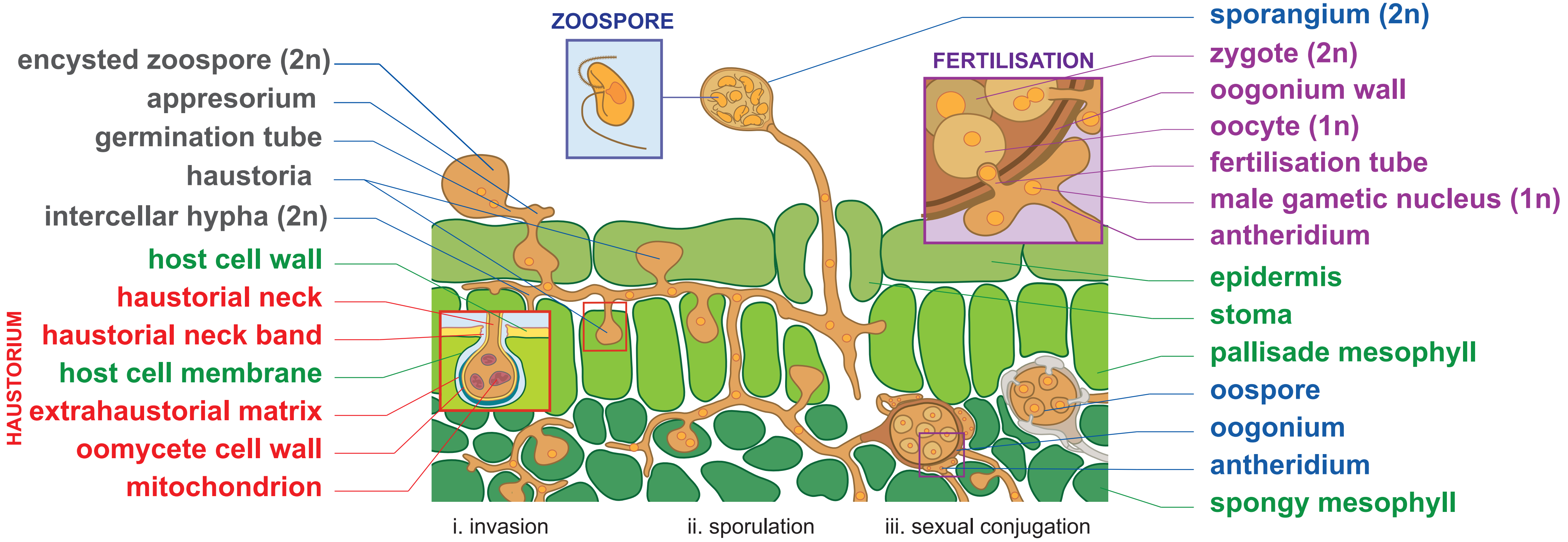

Synurophyte

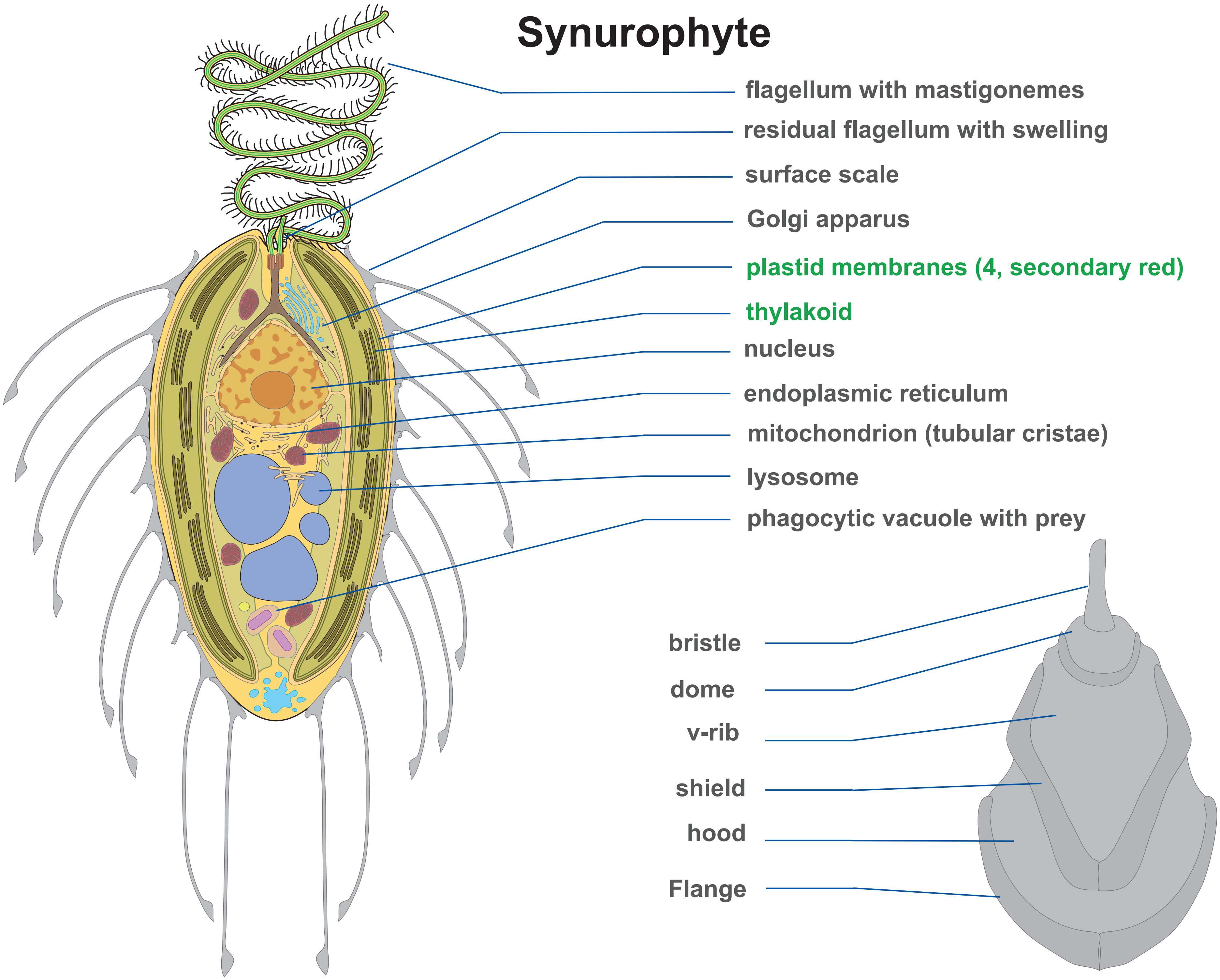

Diatom

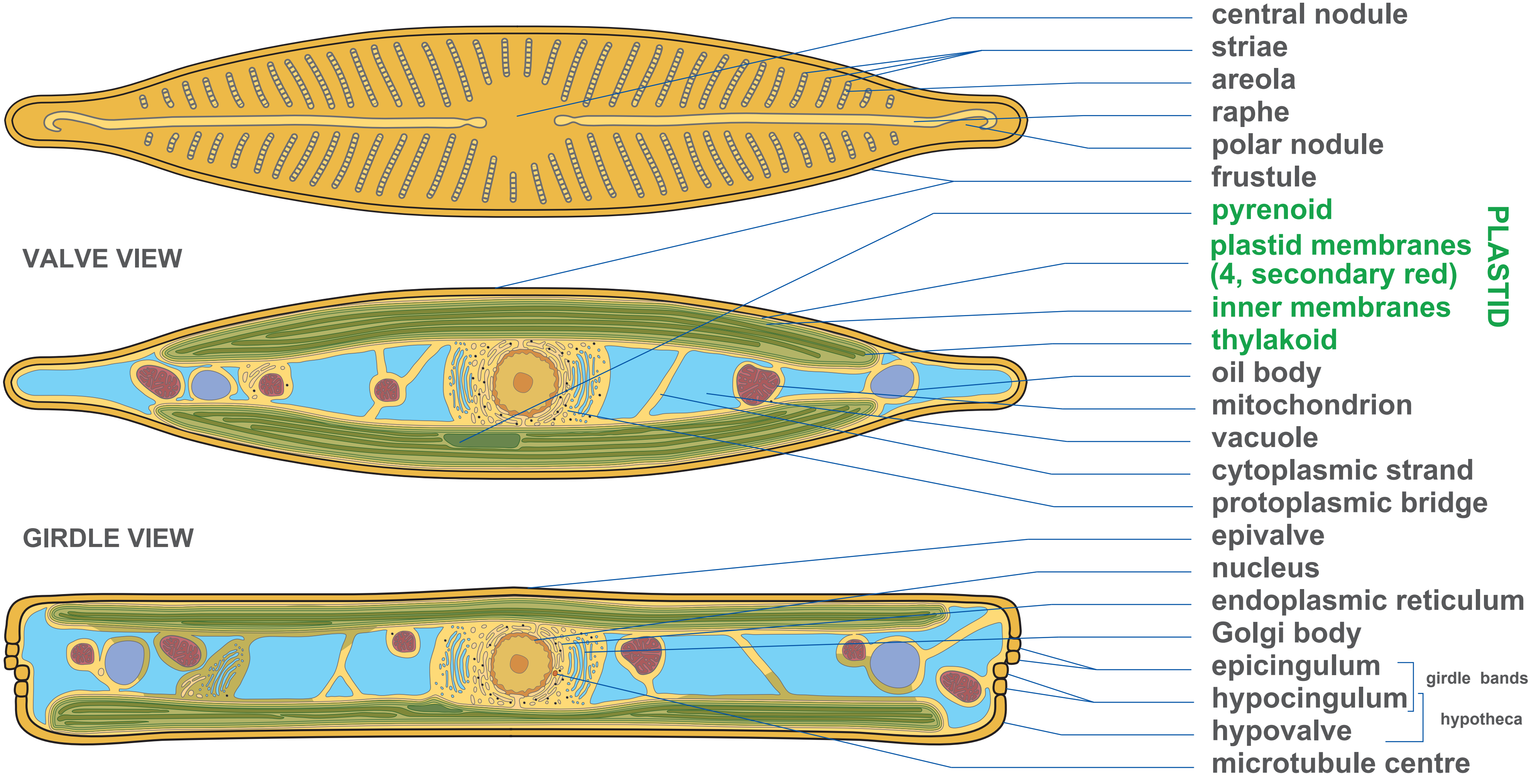
VALVE VIEW
GIRDLE VIEW
central nodule
striae
areola
raphe
polar nodule
frustule
pyrenoid
plastid membranes
(4, secondary red)
inner membranes
thylakoid
PLASTID
oil body
mitochondrion
vacuole
cytoplasmic strand
protoplasmic bridge
epivalve
nucleus
endoplasmic reticulum
Golgi body
epicingulum
hypocingulum
hypovalve
microtubule centre
girdle bands
hypotheca
