# Supplementary material for: Openly available illustrations as tools to describe eukaryotic microbial diversity
Source: PLoS Biol. 2023 Nov 21;21(11):e3002395. doi: 10.1371/journal.pbio.3002395 (PMC10662721; doi:10.1371/journal.pbio.3002395)

Kinetoplastid

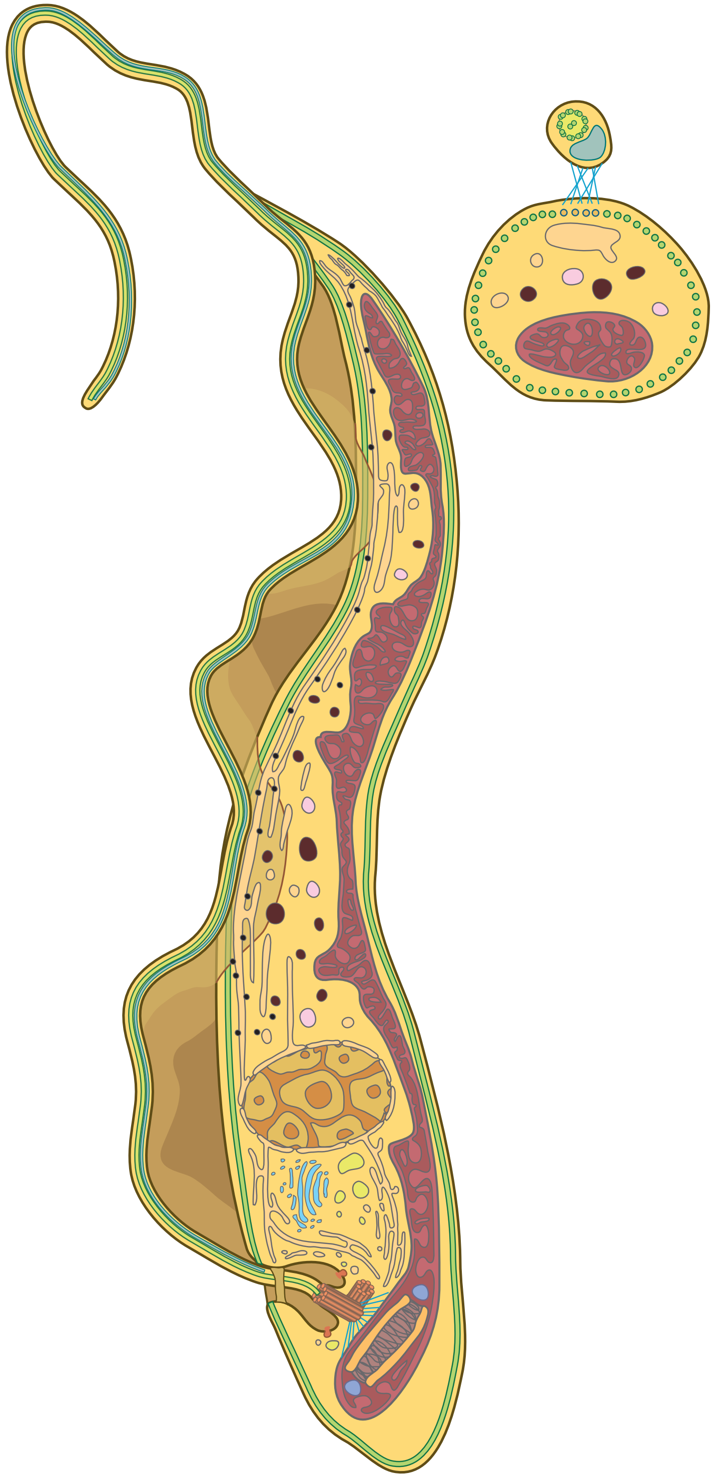

Euglenid

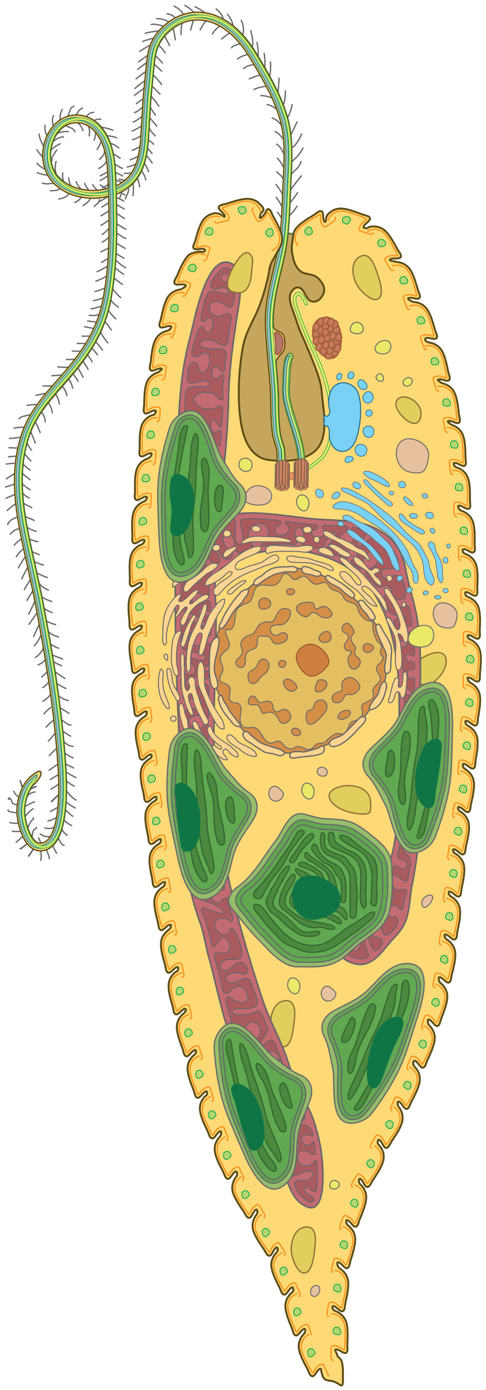

Heterolobosean

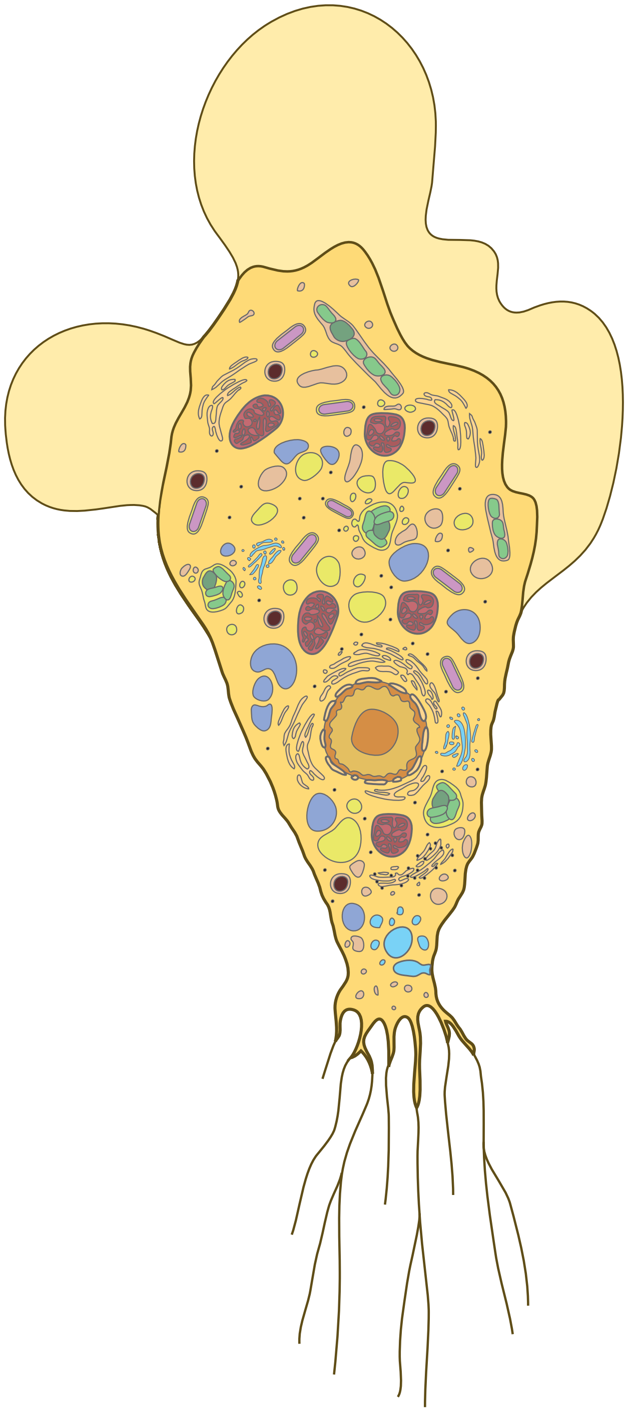

Jakobid

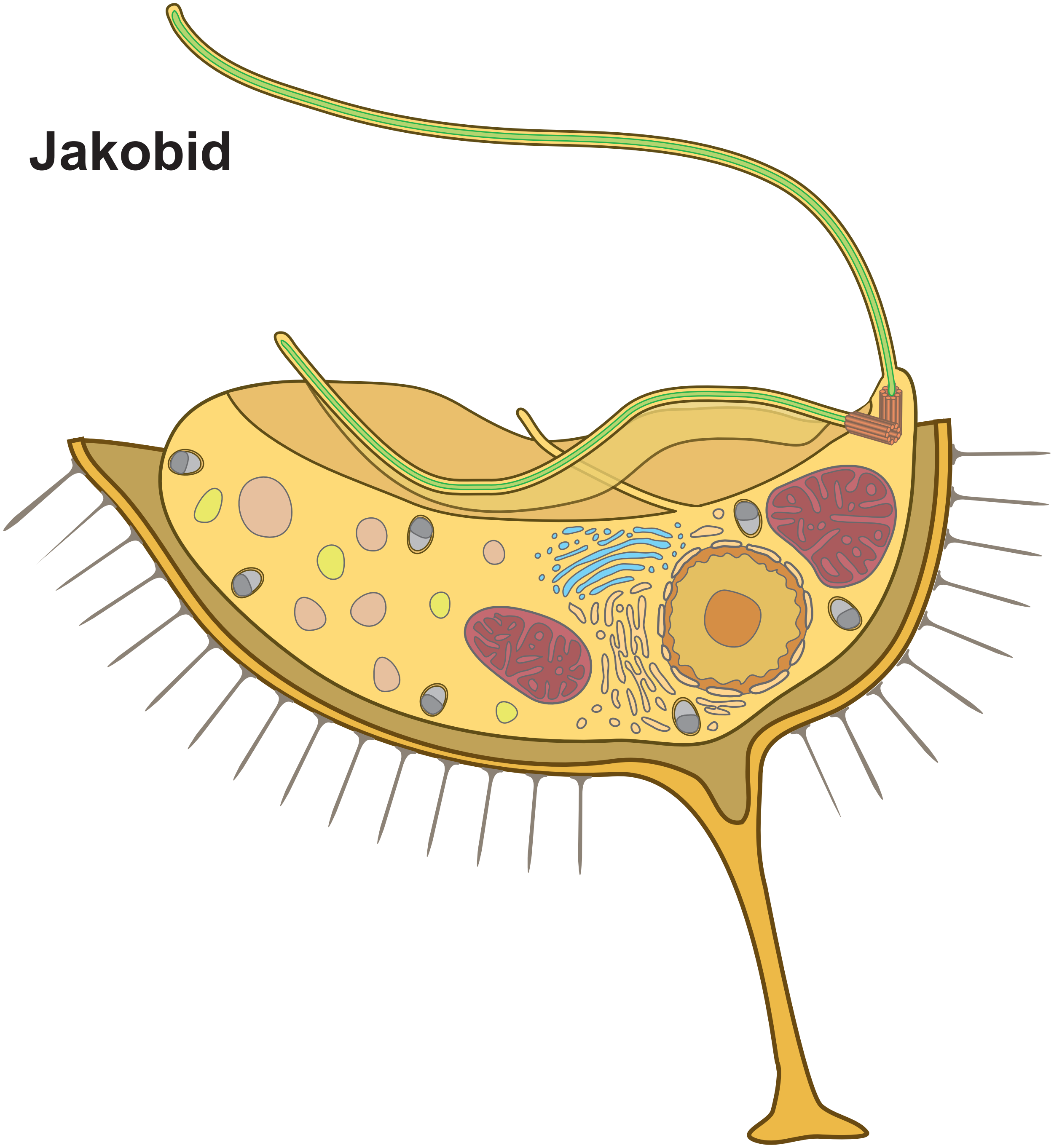

Diplomonad

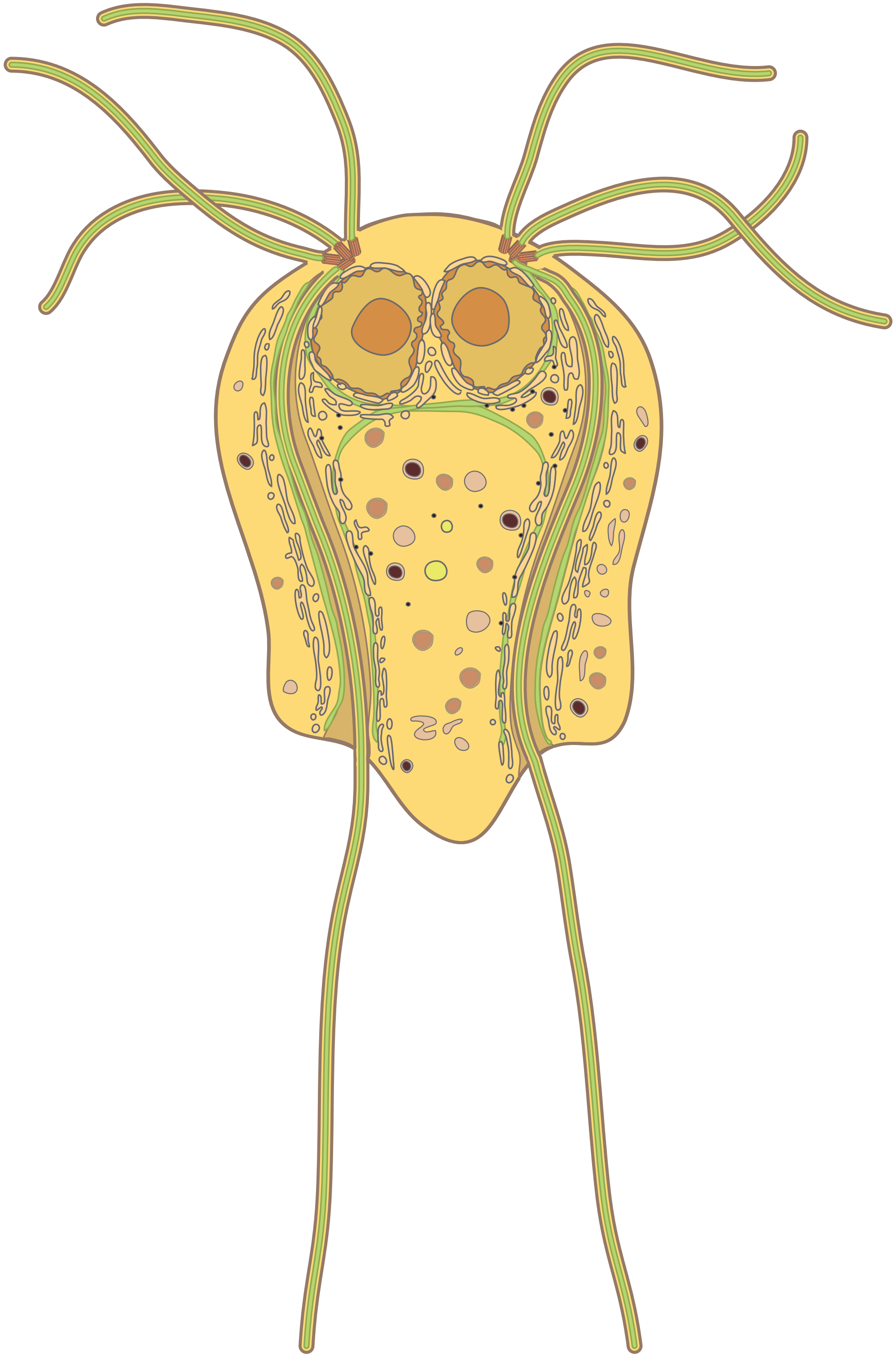

Oxymonad

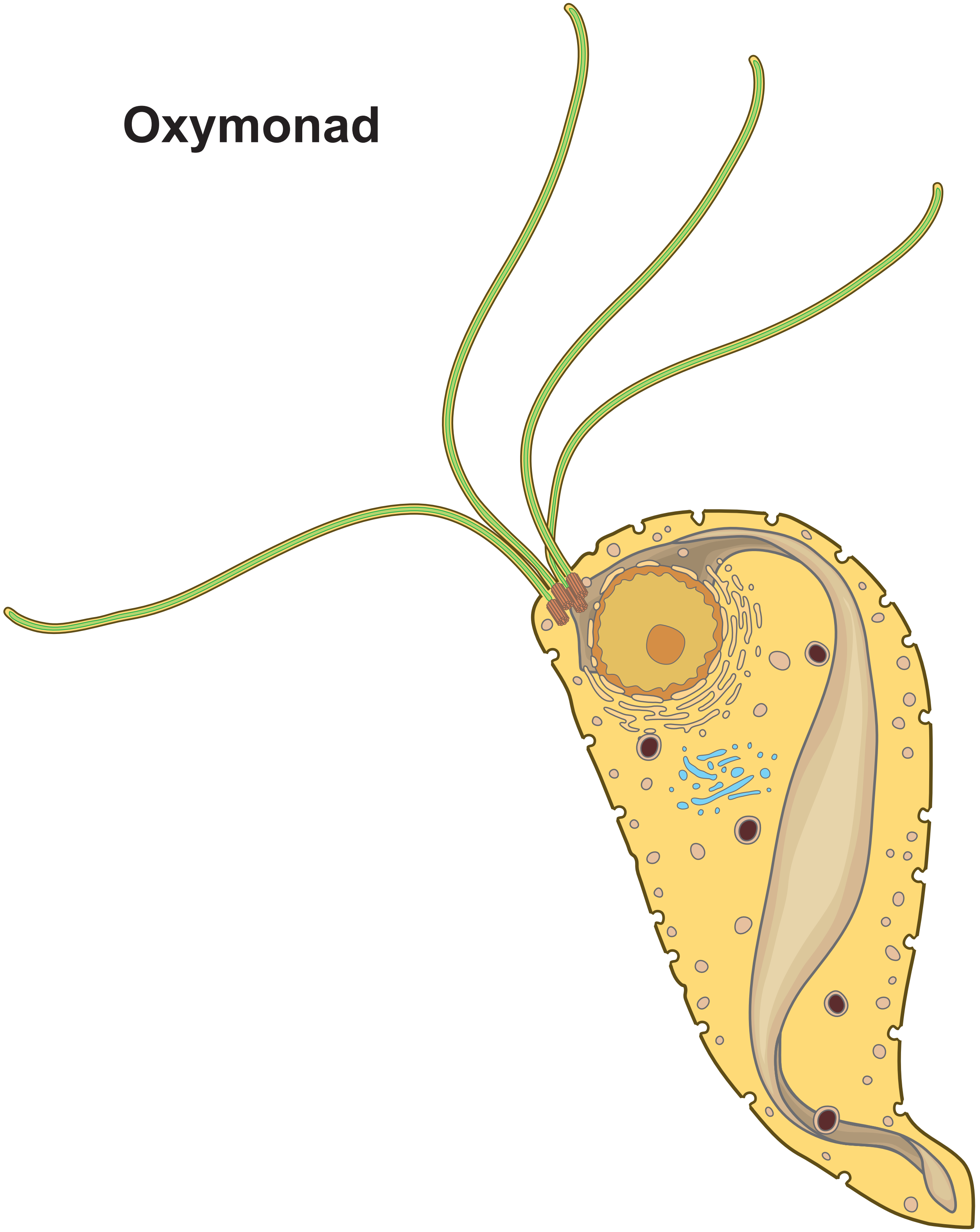

Parabasalian

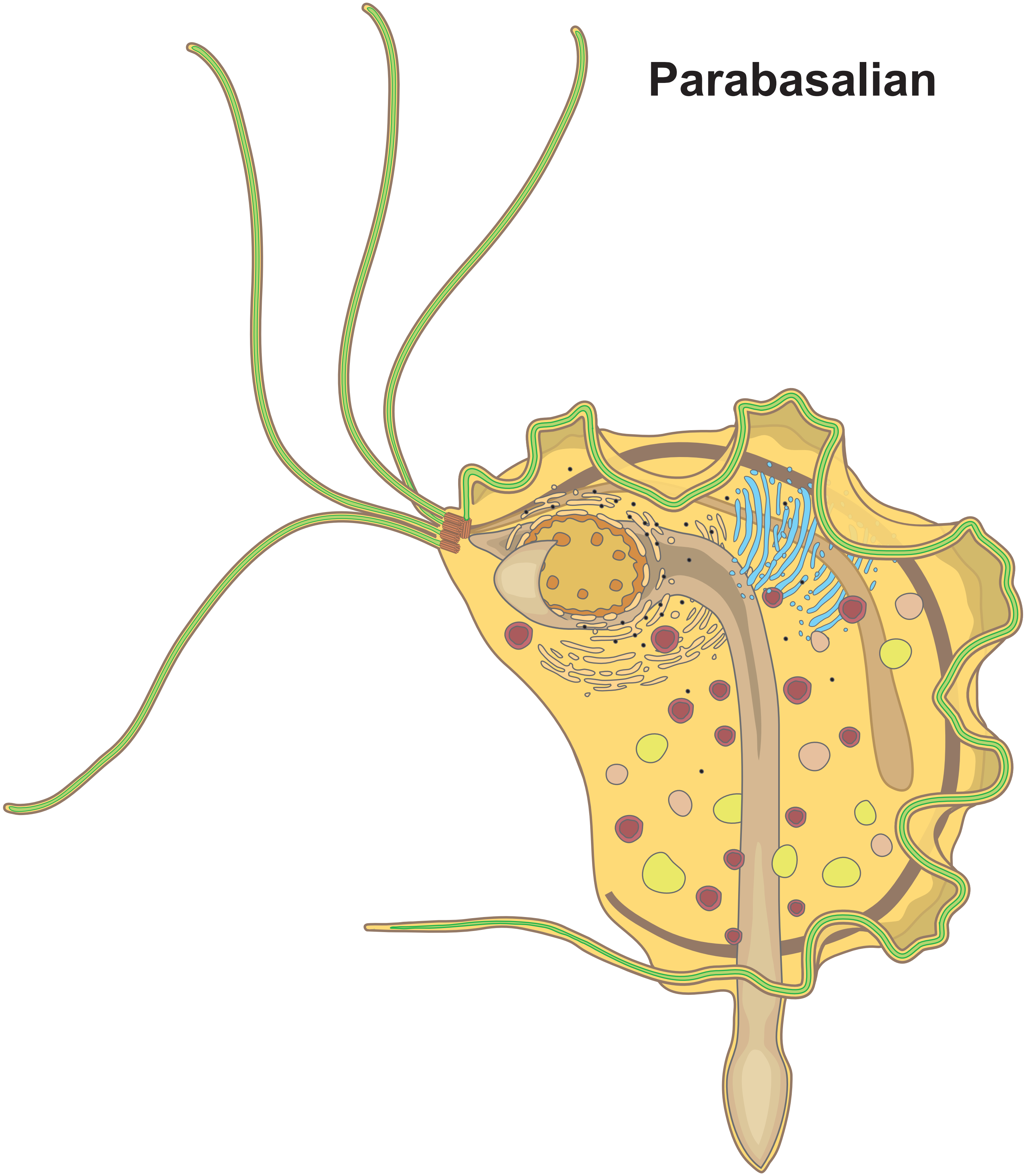

Myxogastrid

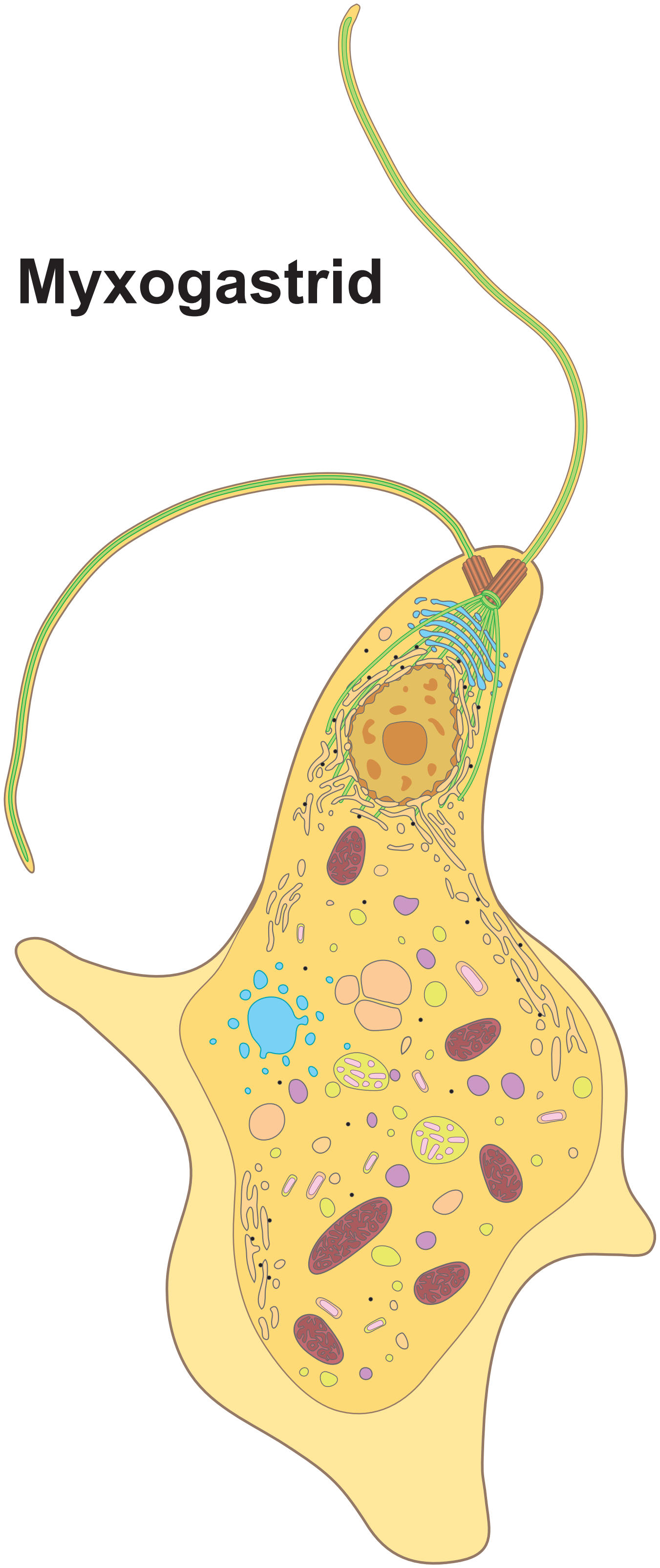

Arcellinid
(Tubulinid)

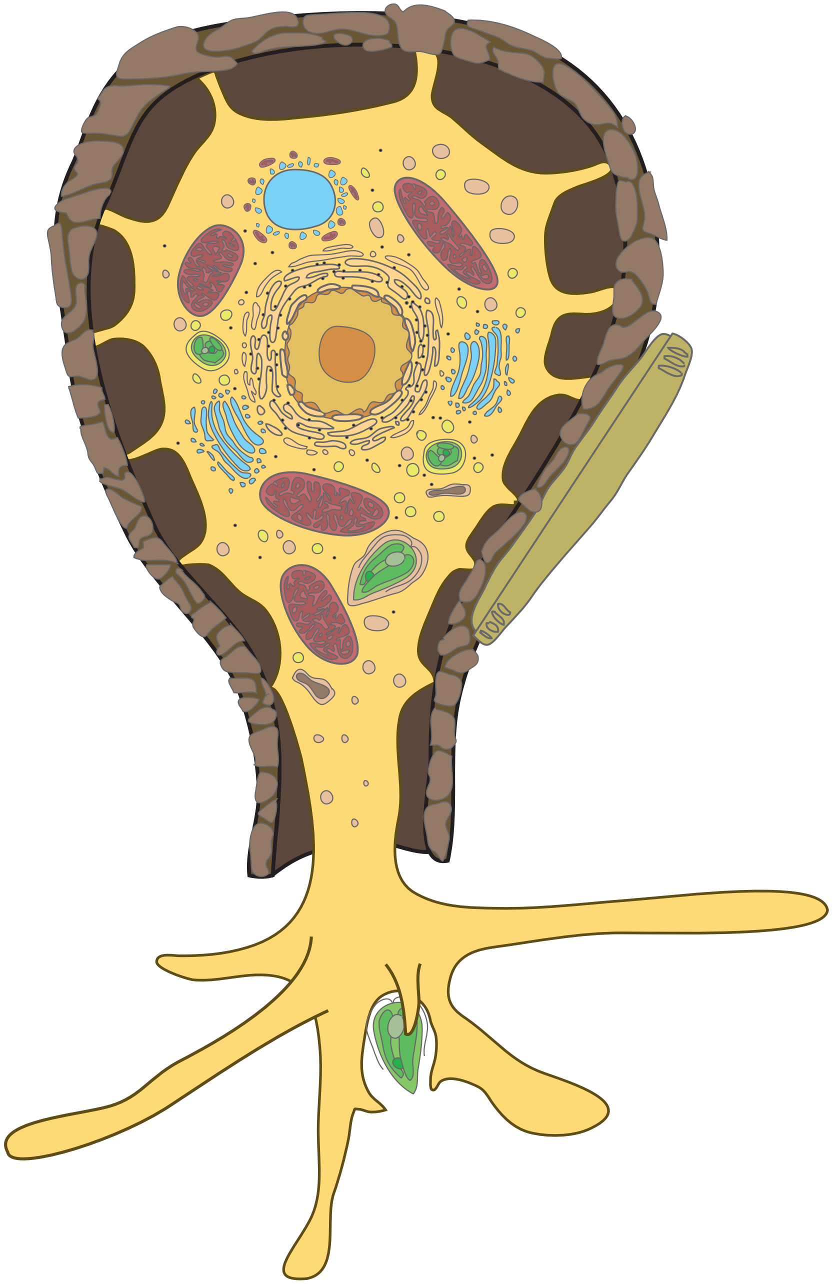

Lobosean
(Tubulinid)

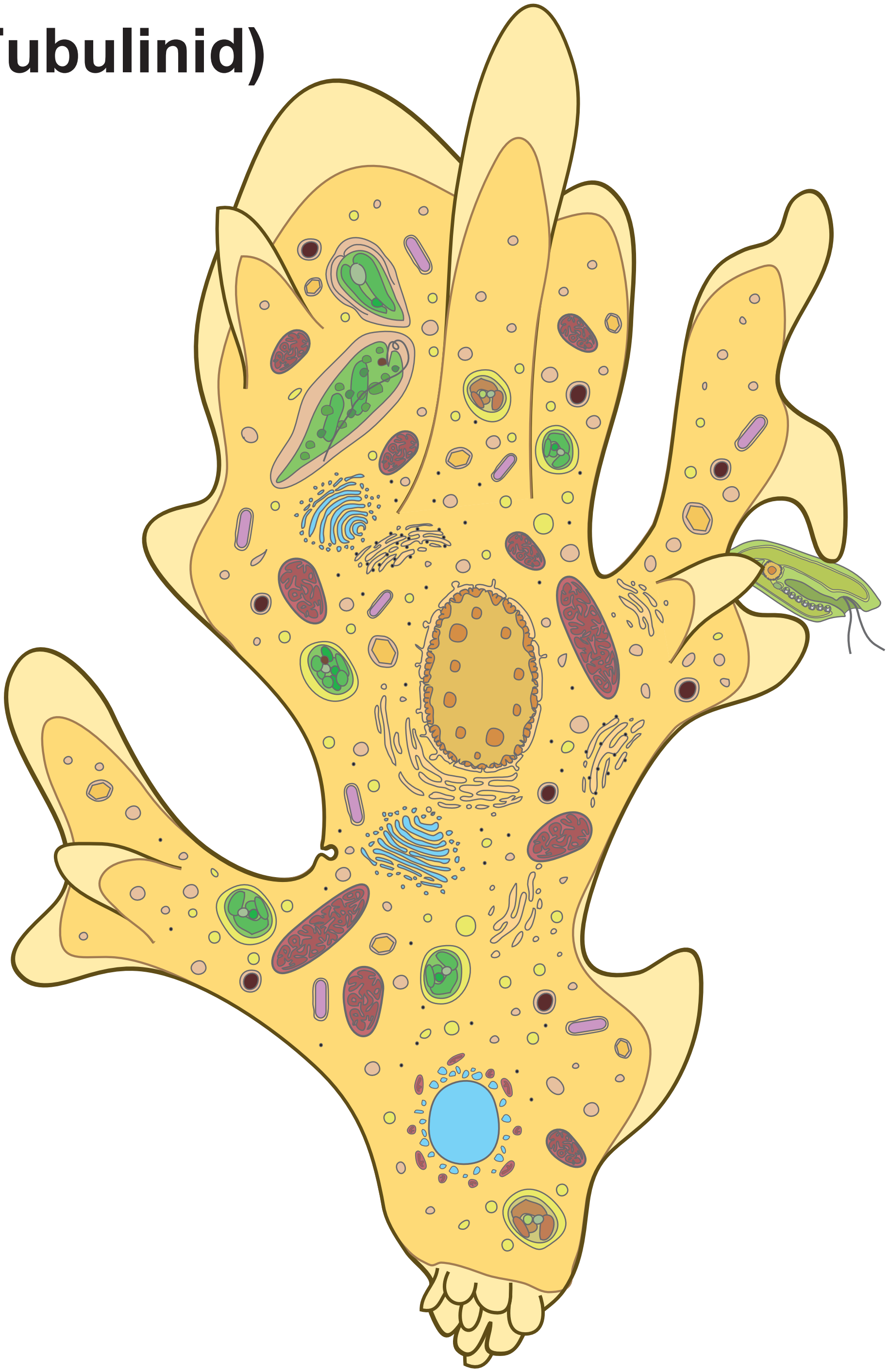

Chytrid

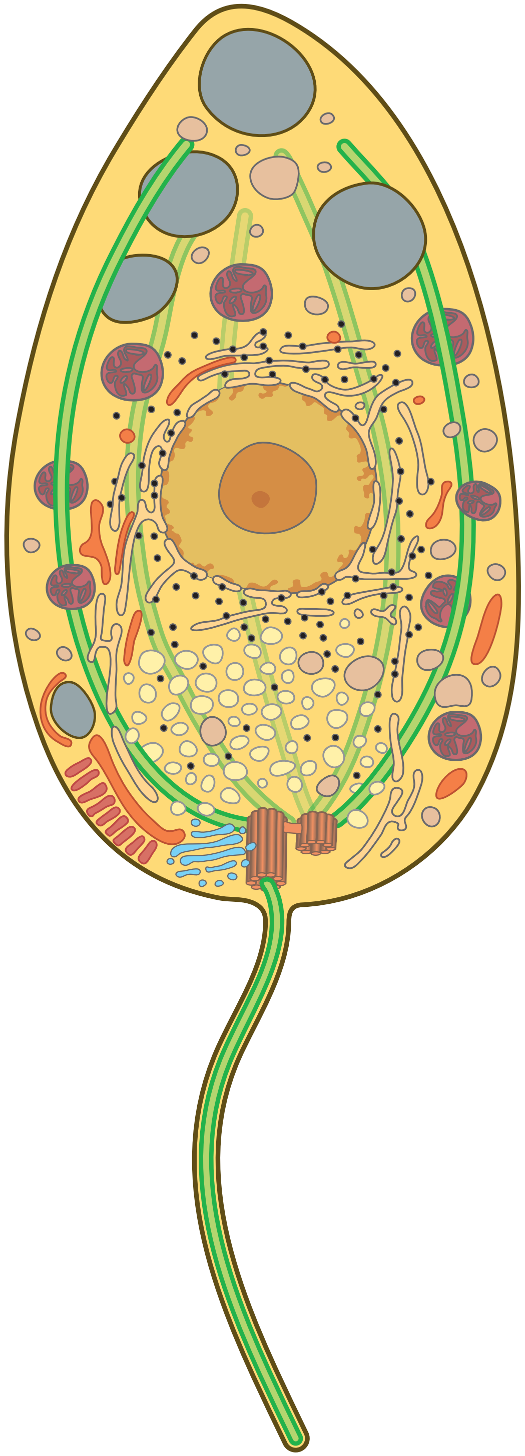

Choanoflagellate

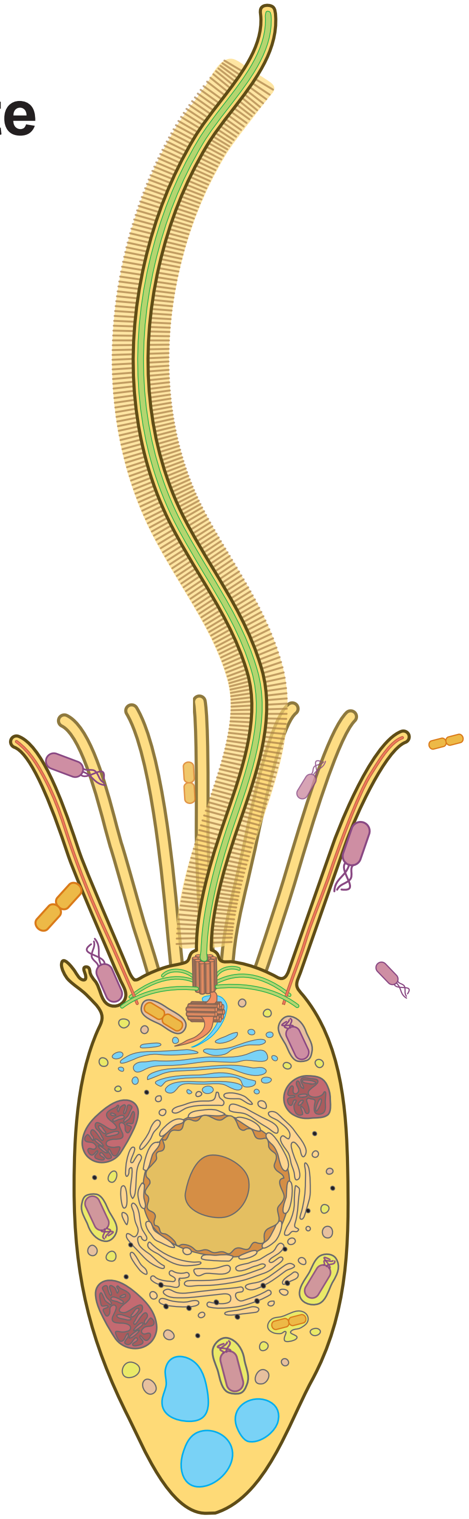

Choanocyte
(Poriferan feeding cell)

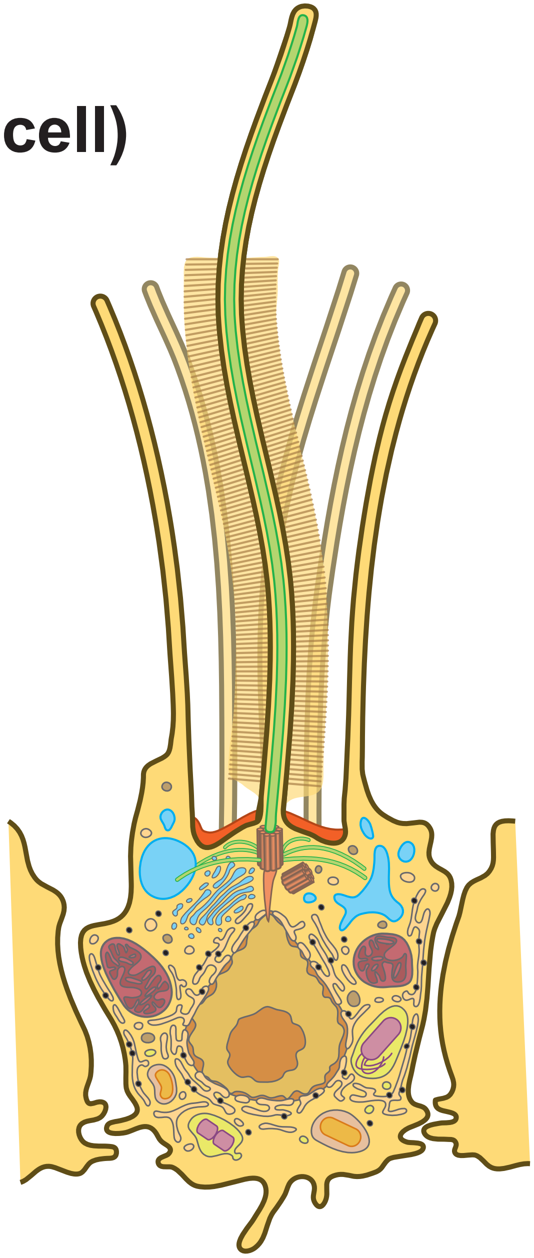

Charophyte

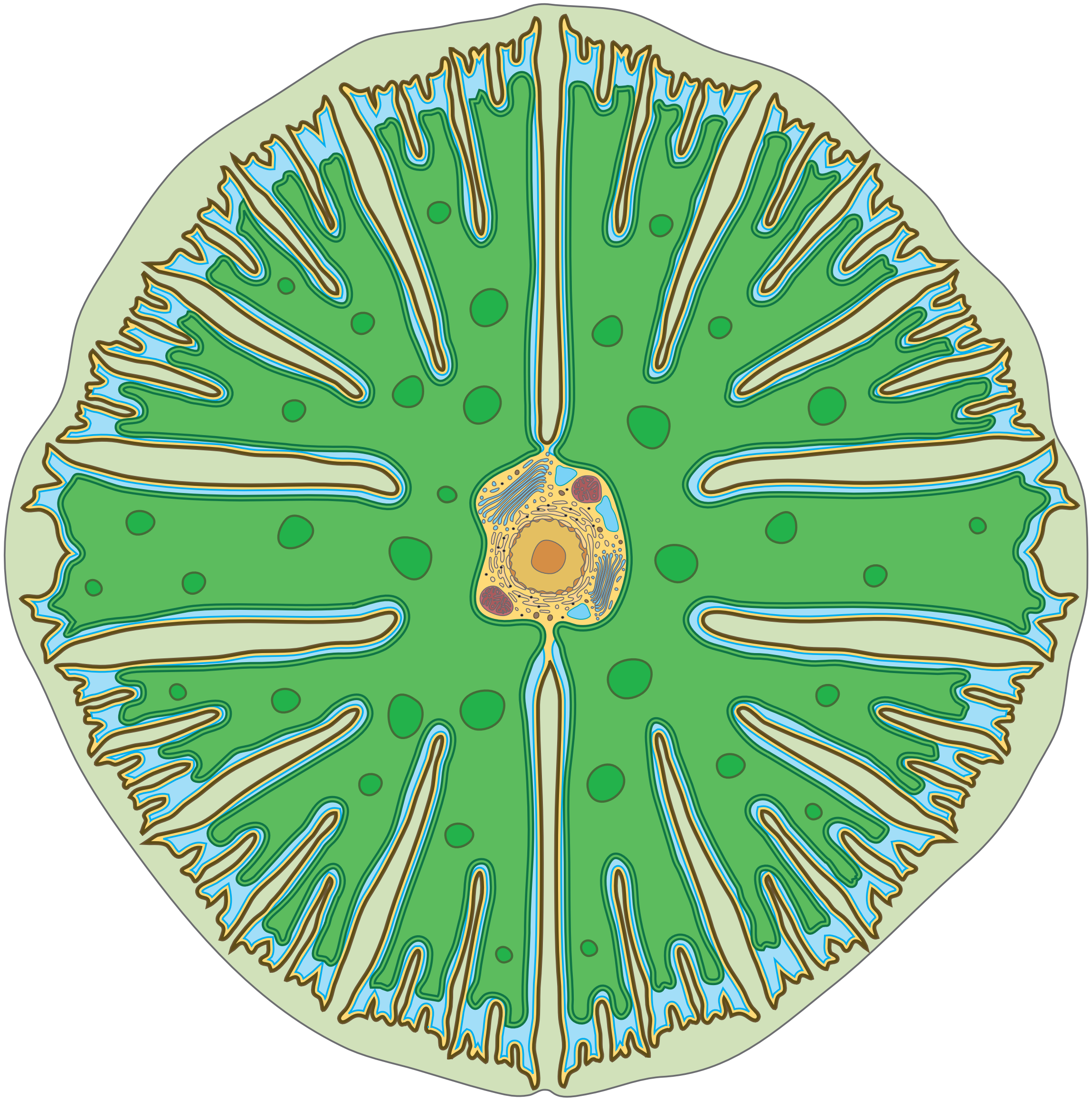

Prasinophyte

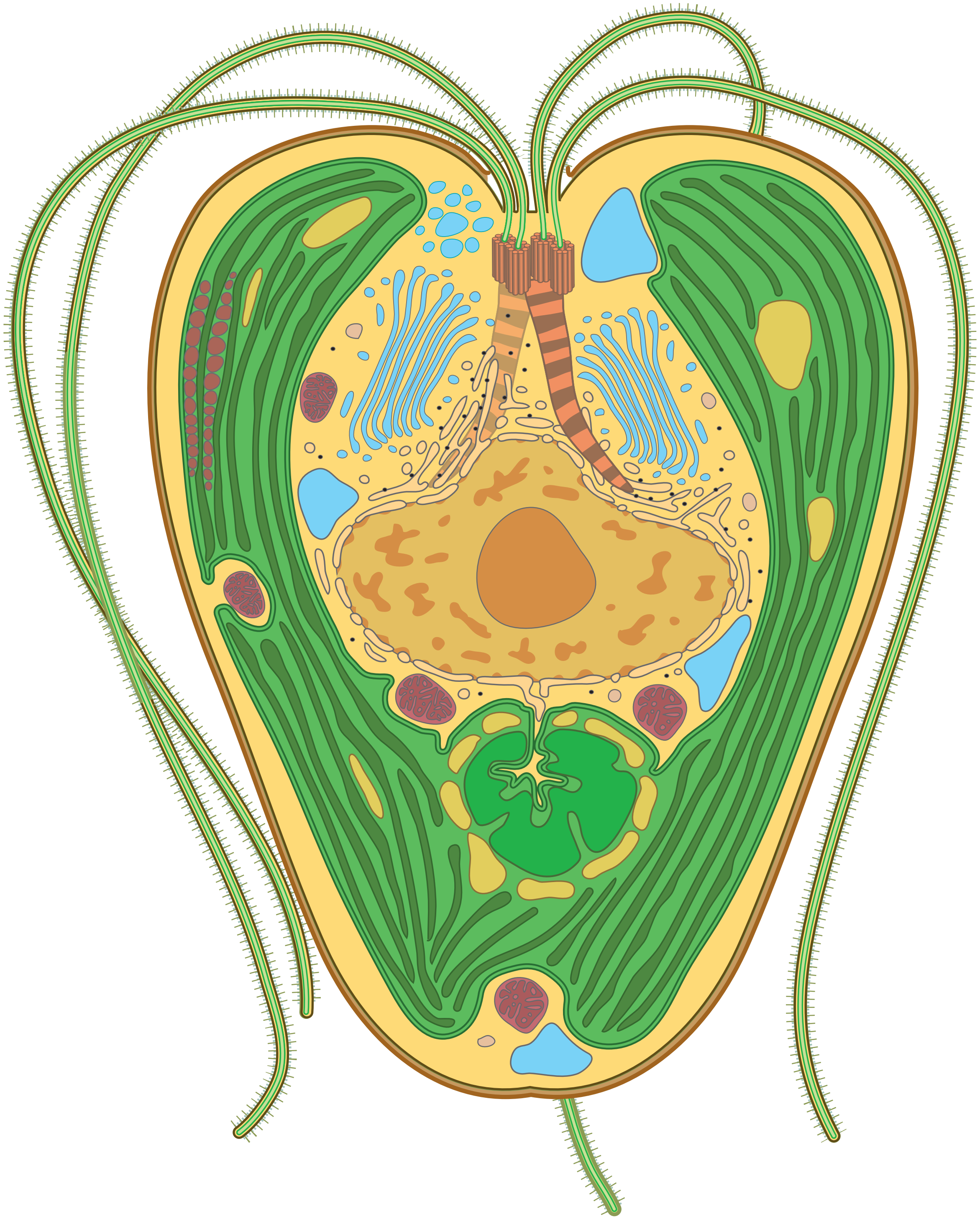

Rhodophyte
(red alga)

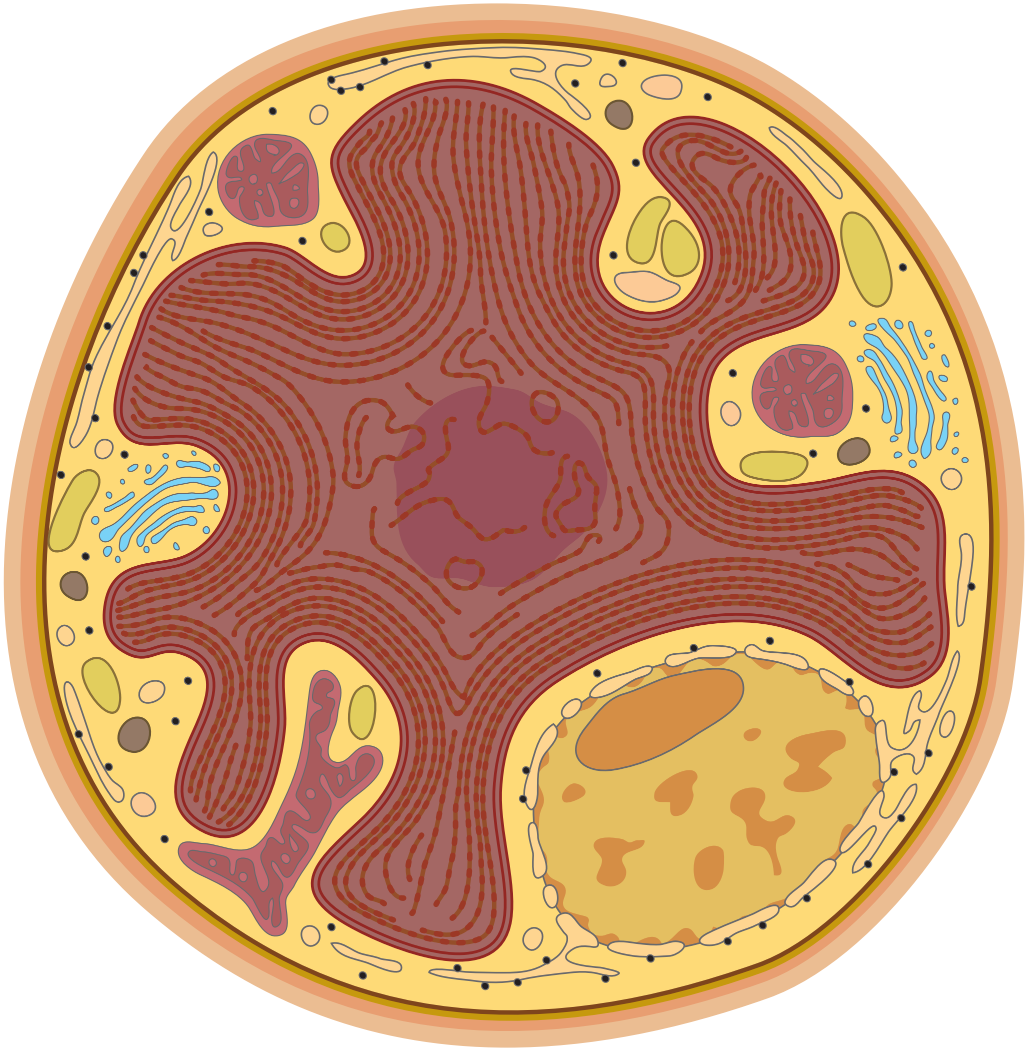

Glaucophyte

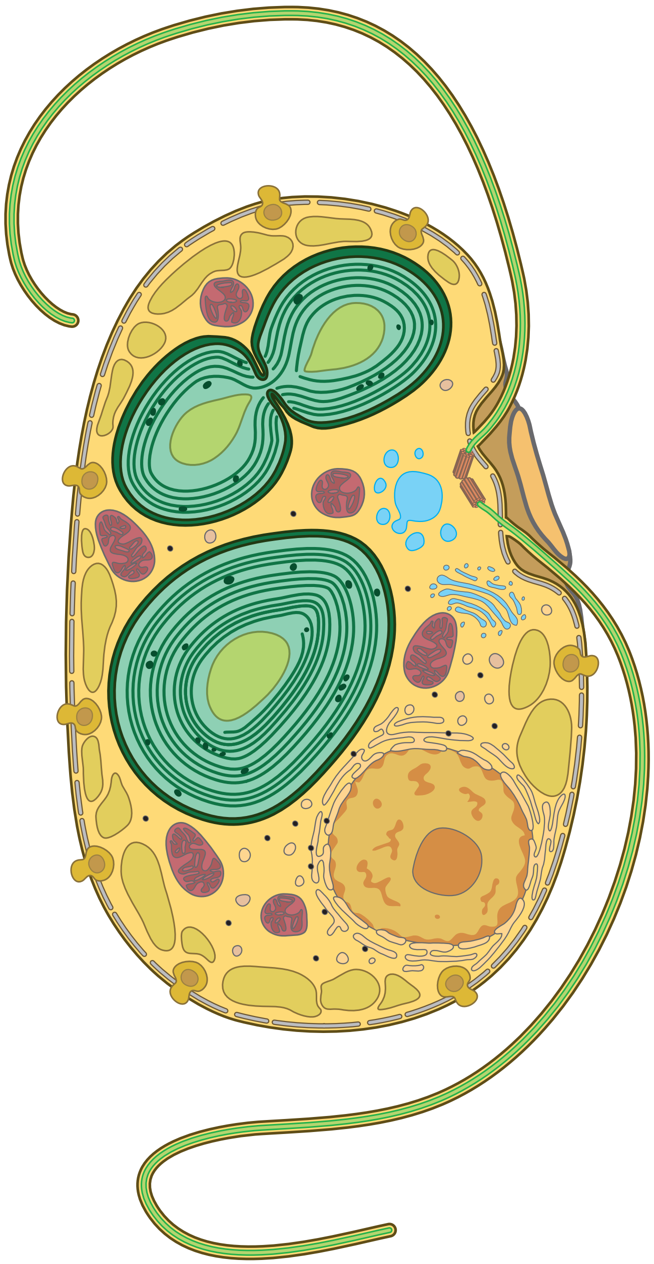

Cryptomonad

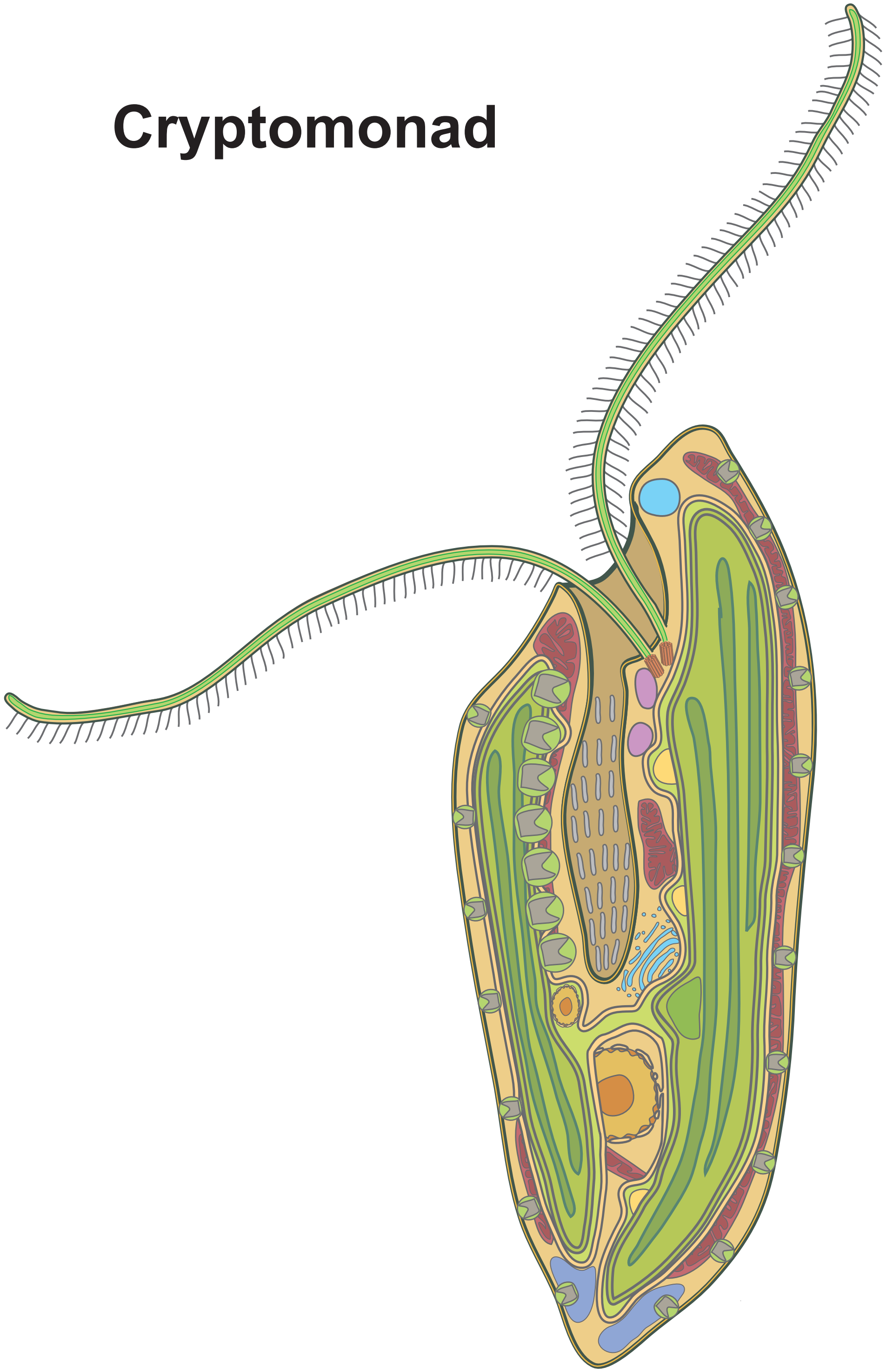

Centrohelid

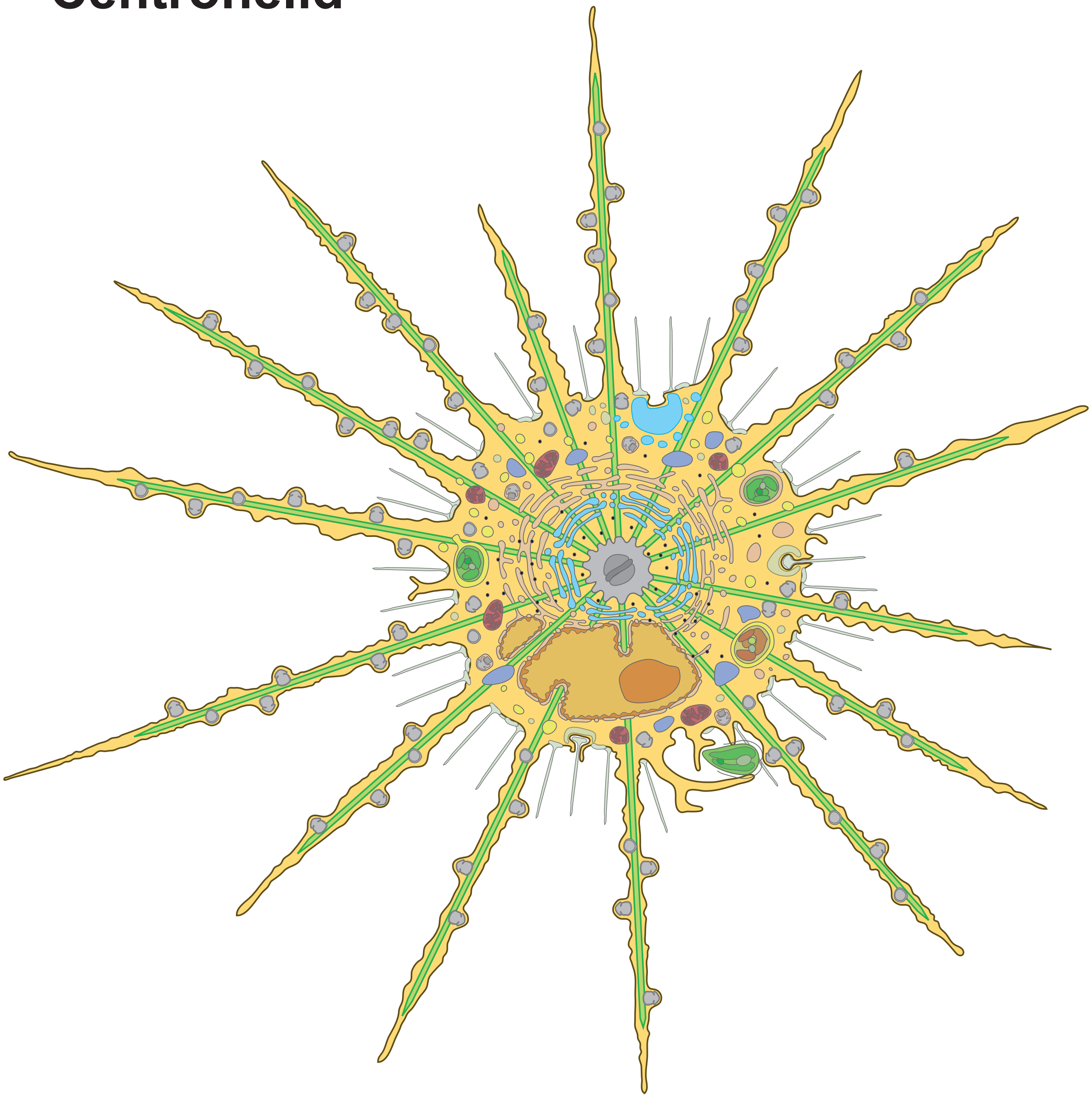

Haptophyte

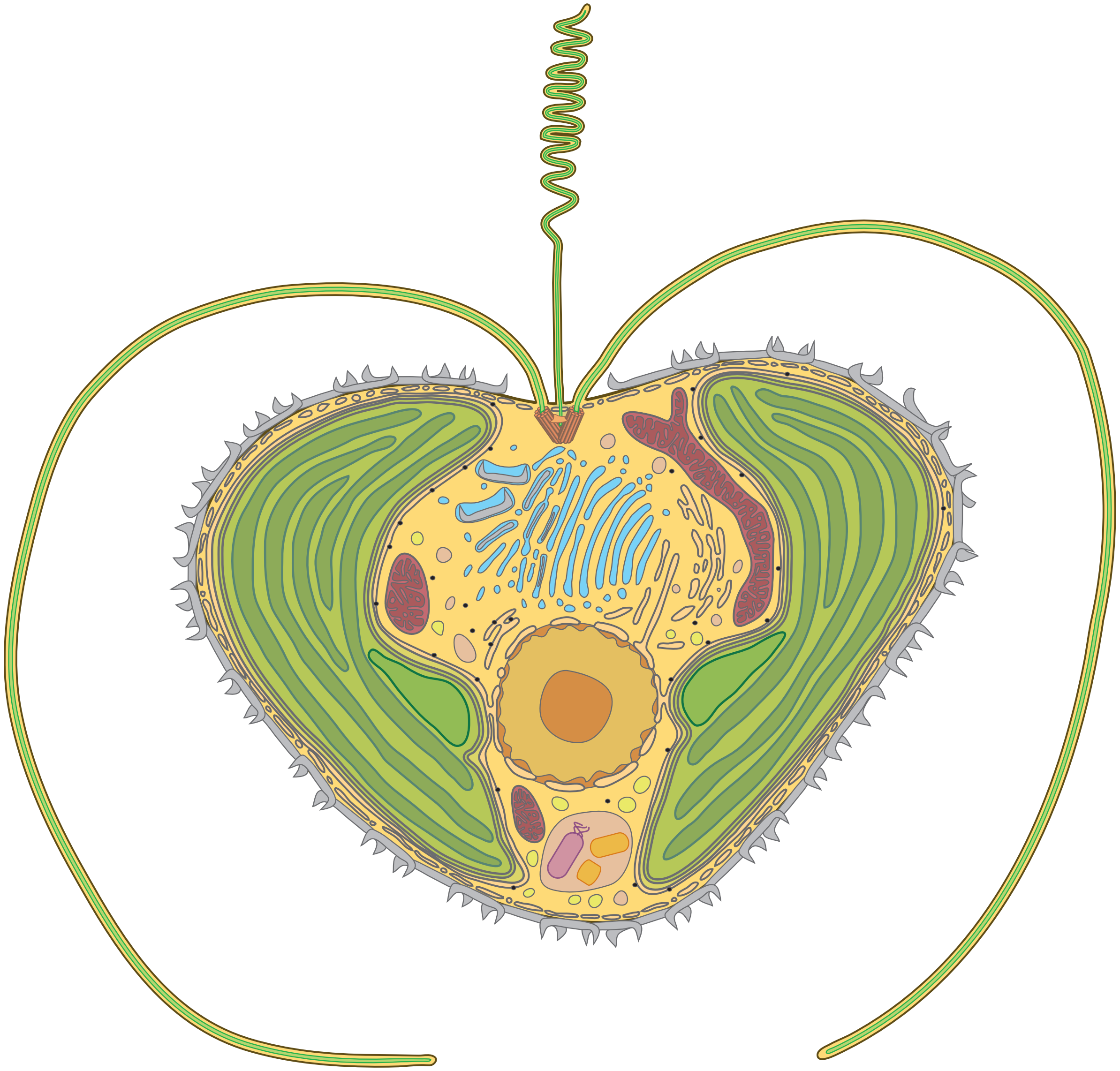

Foraminiferan

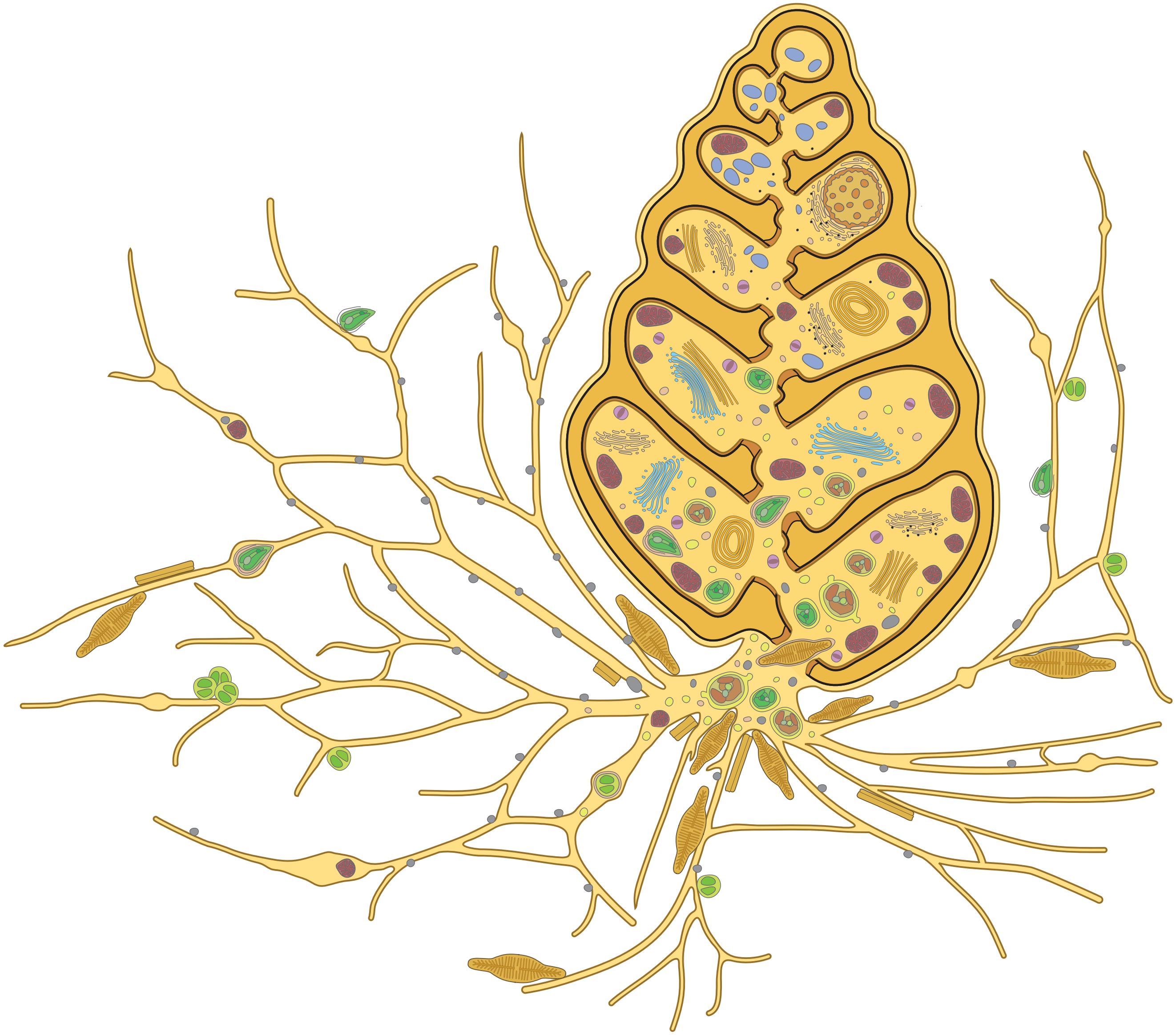

Polycystine

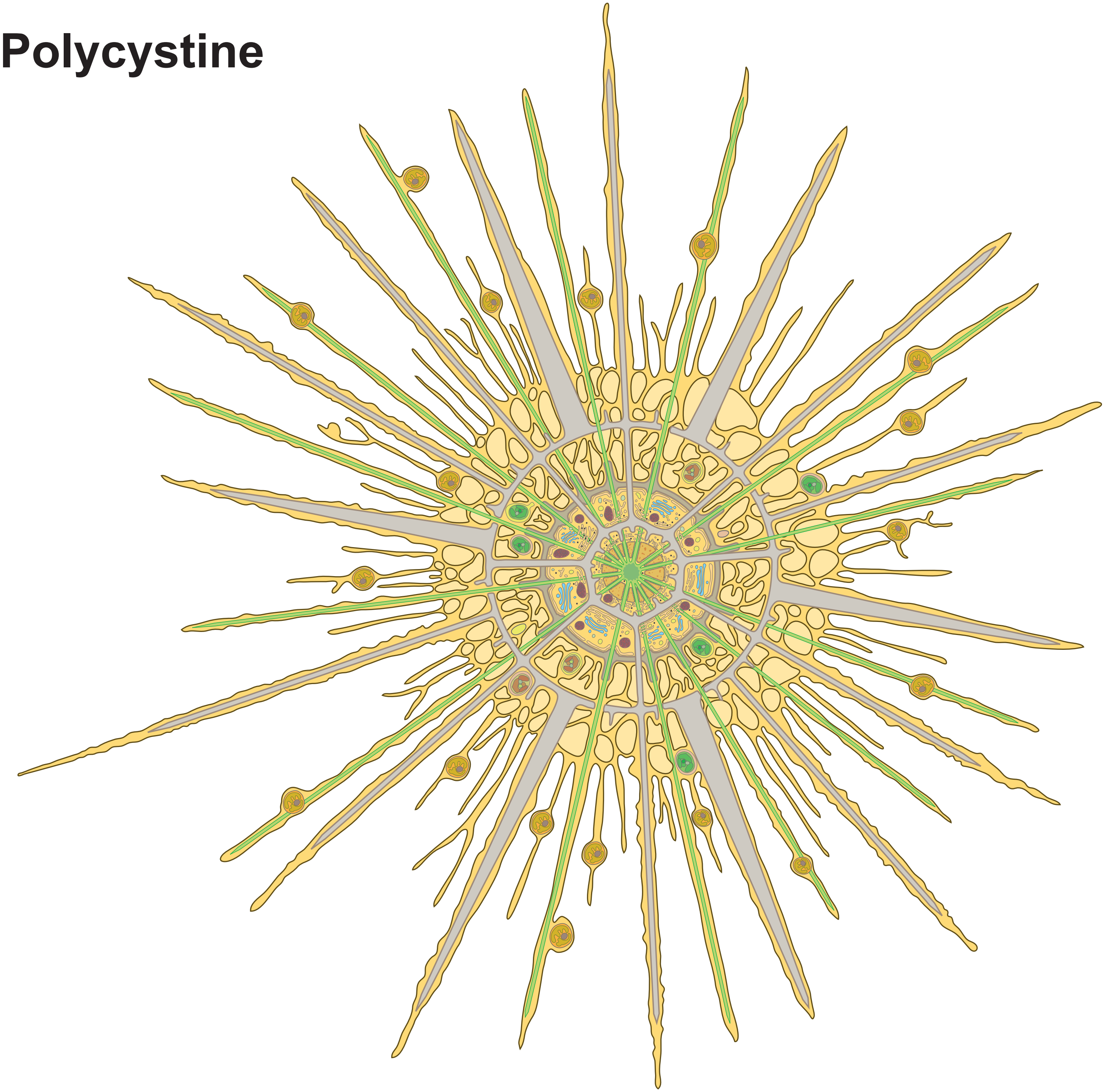

Polycystine (Intracapsulum)

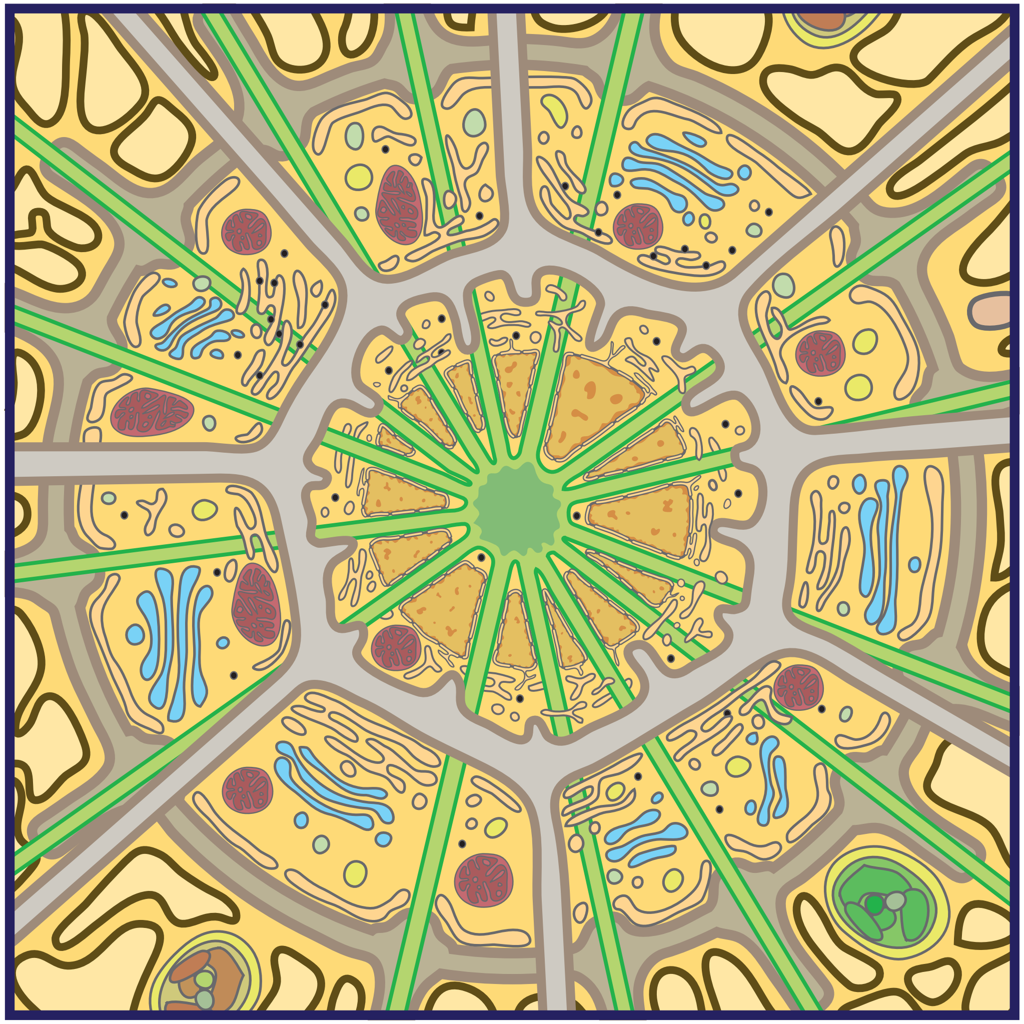

Cercomonad

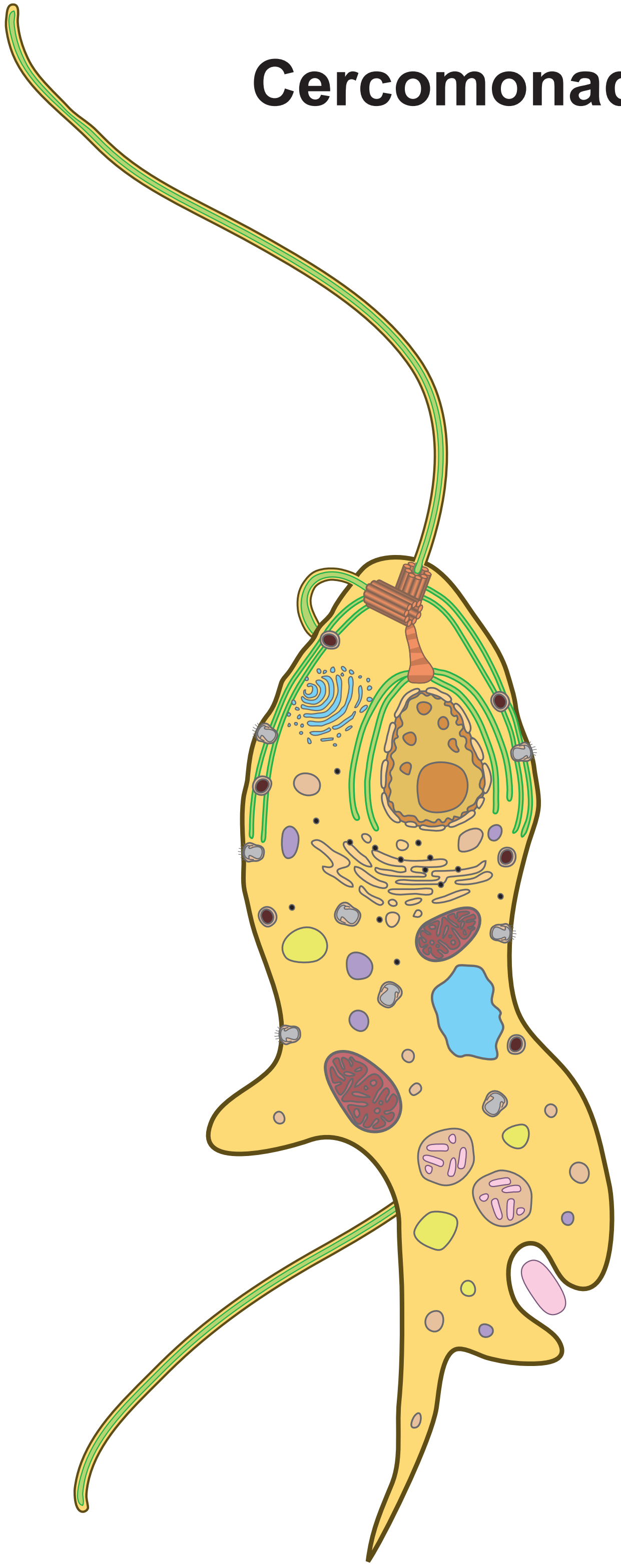

Chlorarachniophyte

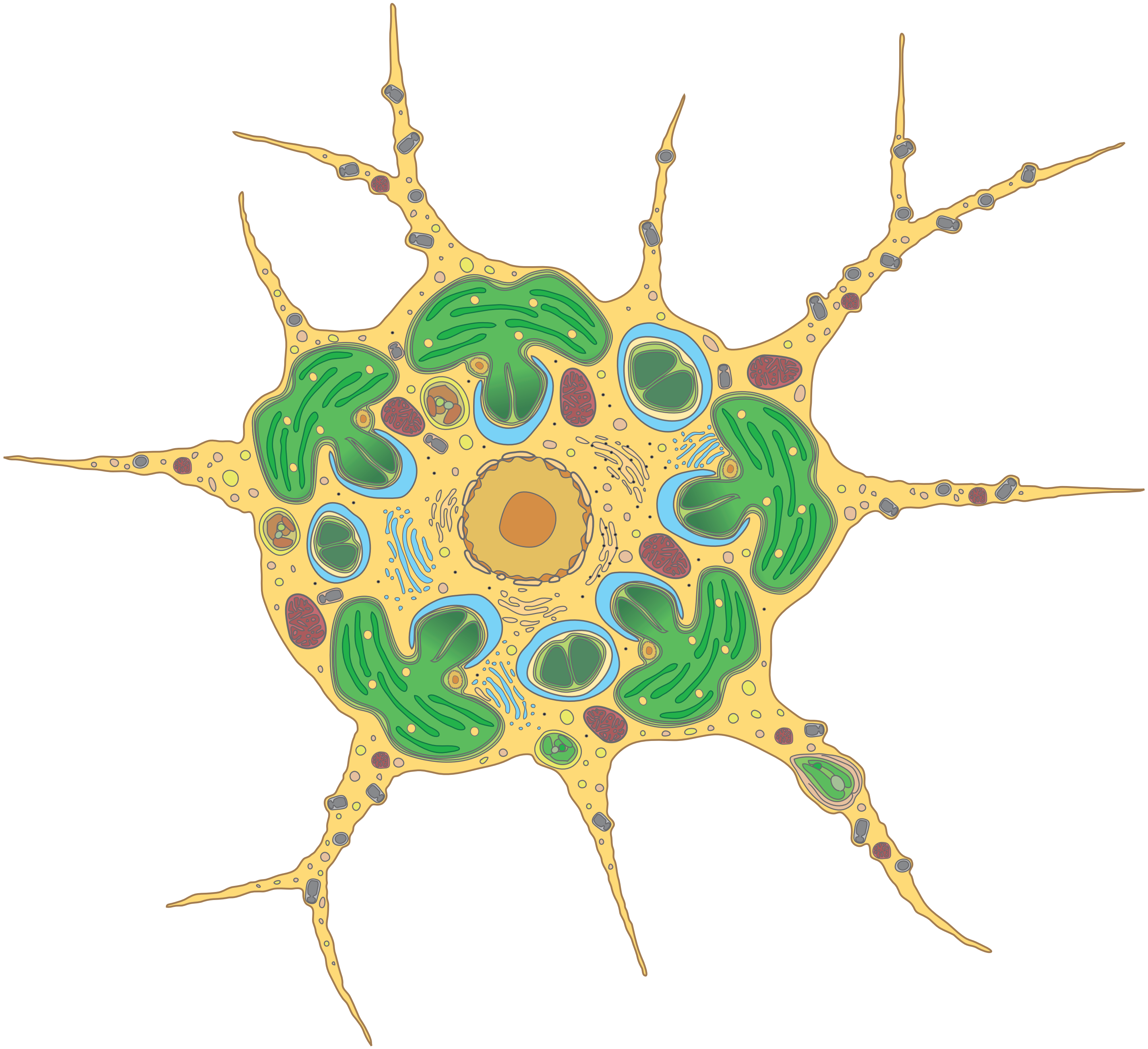

Euglyphid

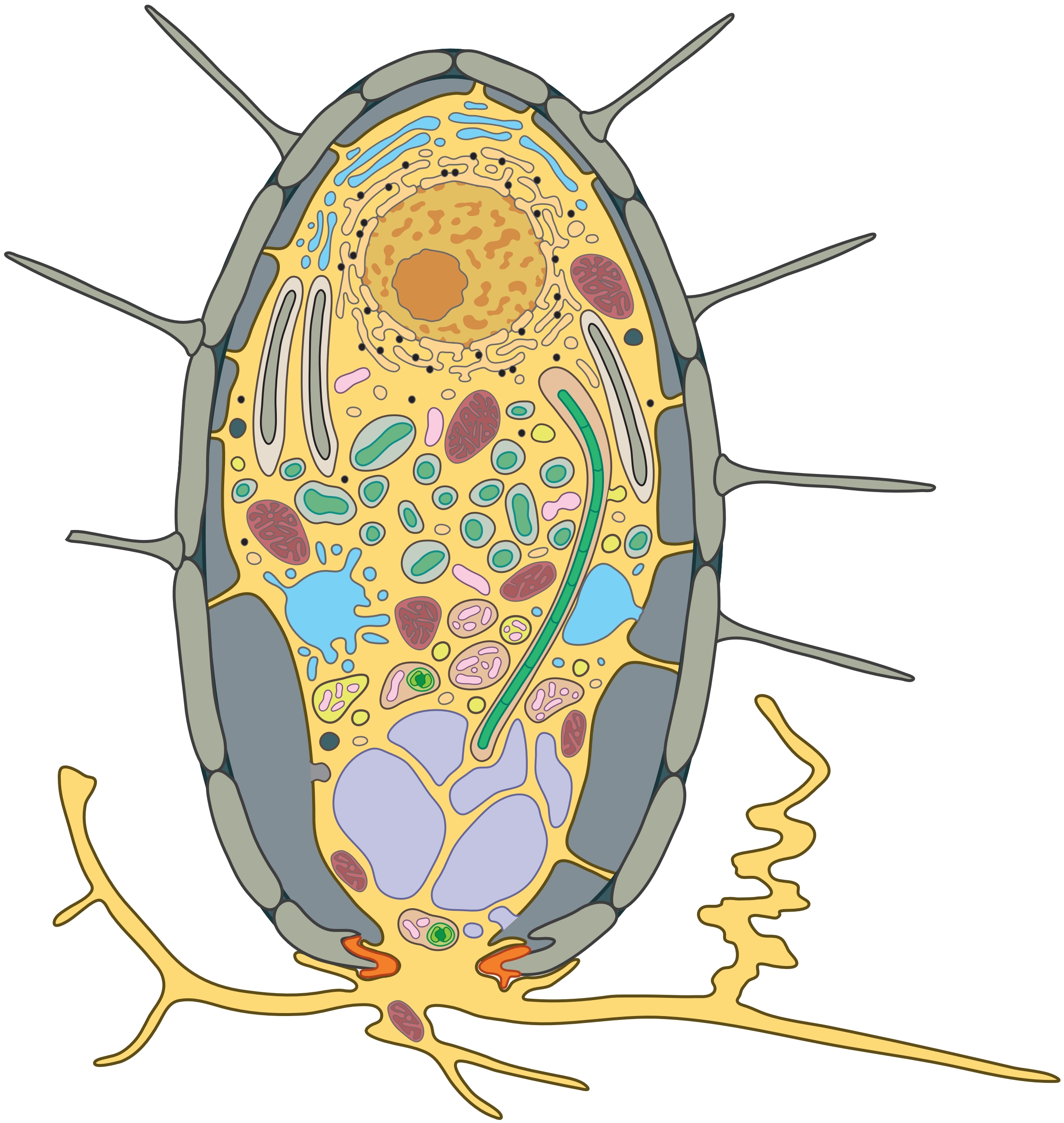

Ciliate

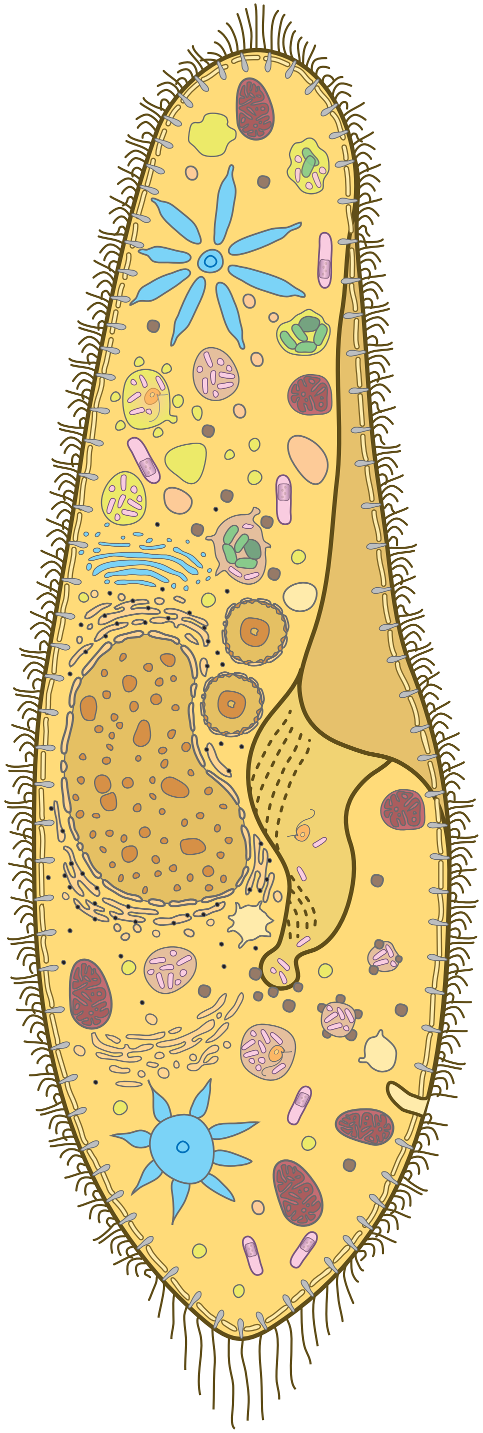

Apicomplexan

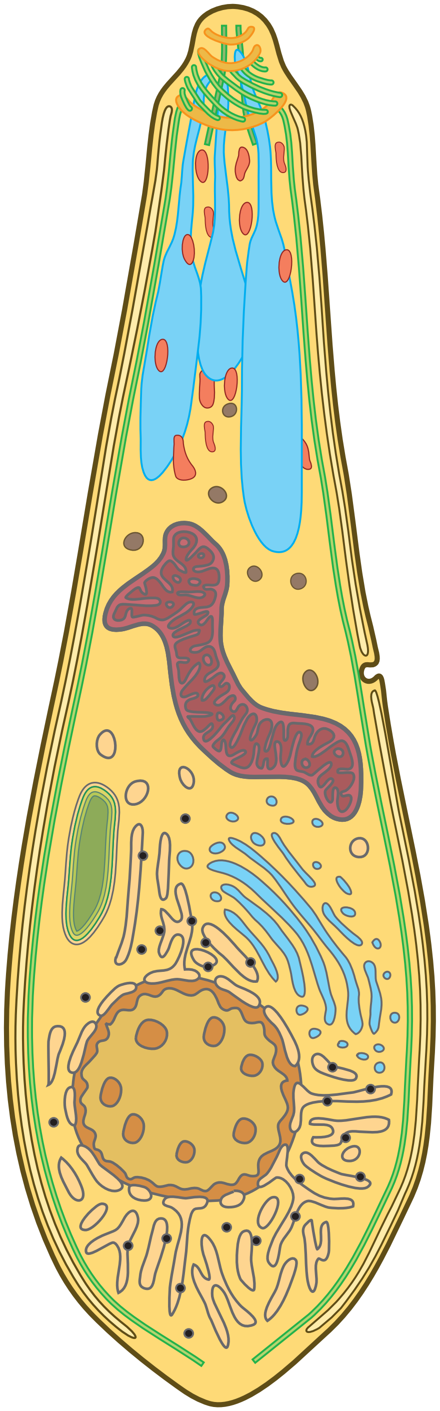

Dinoflagellate

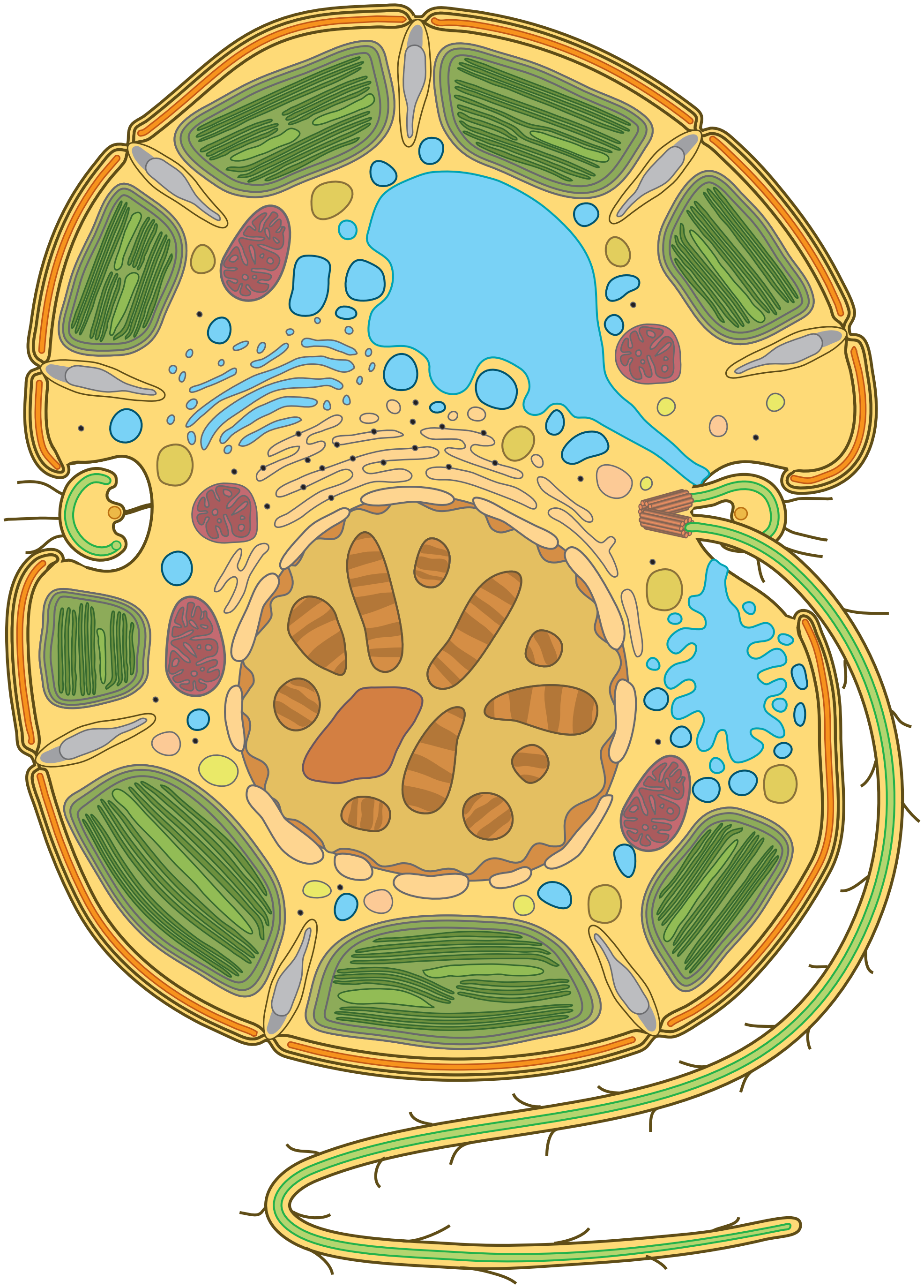

Bicosoecid

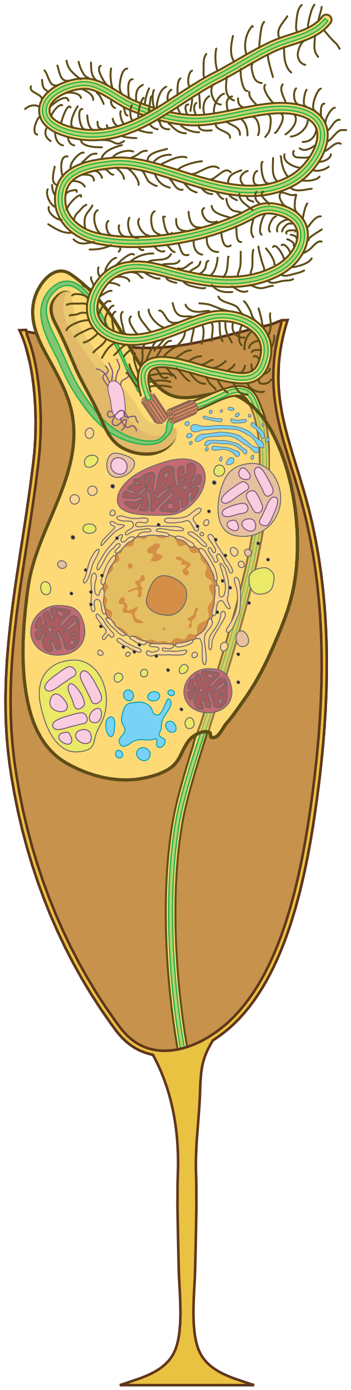

Oomycete

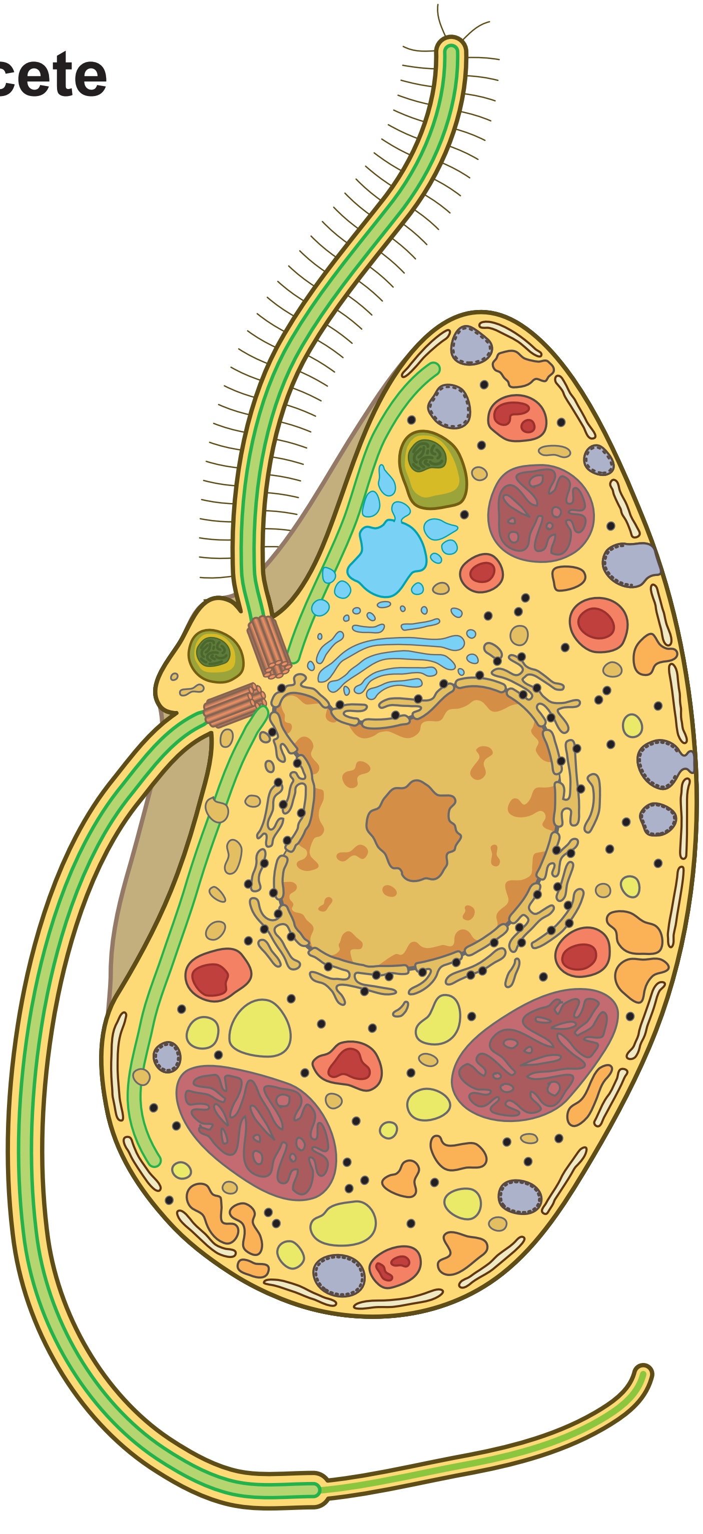

Oomycete invasion

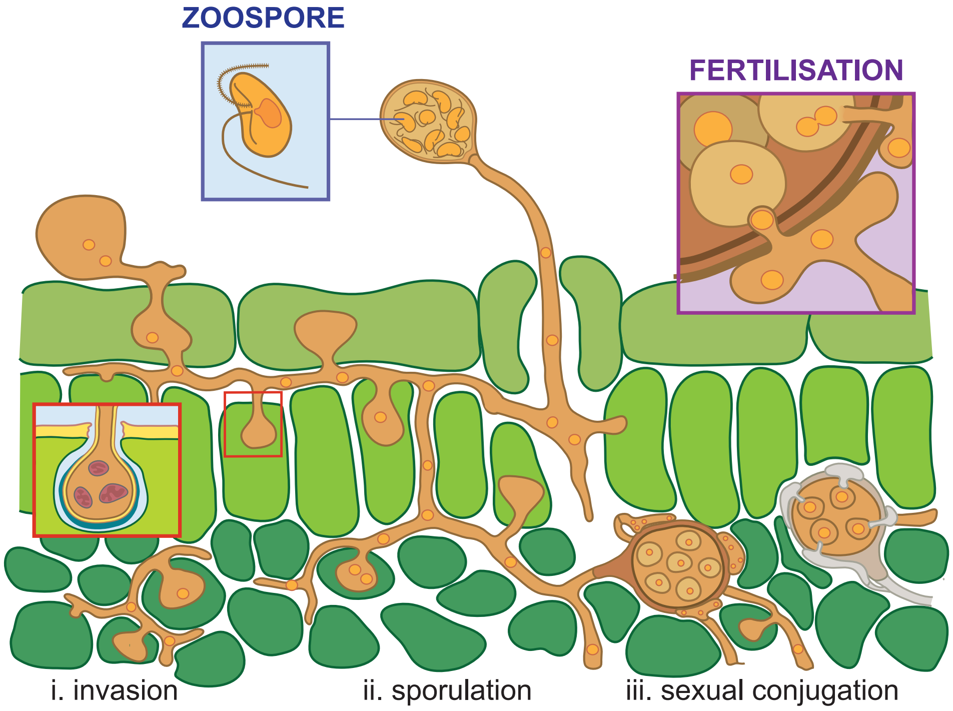

Synurophyte

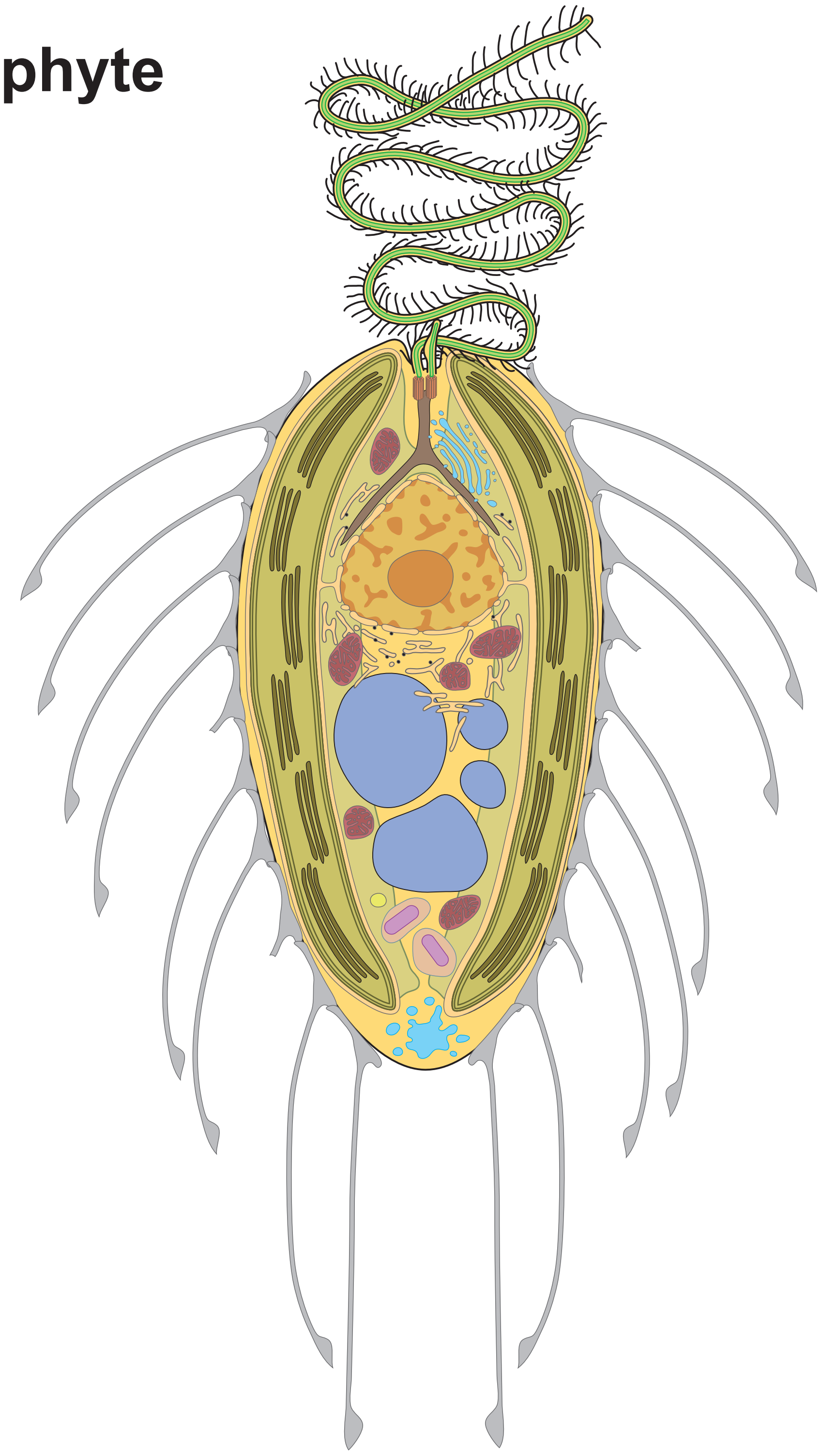

Diatom

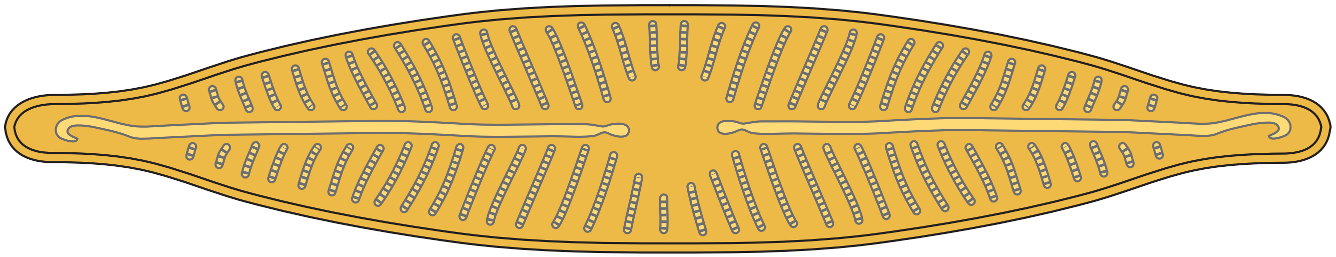

VALVE VIEW

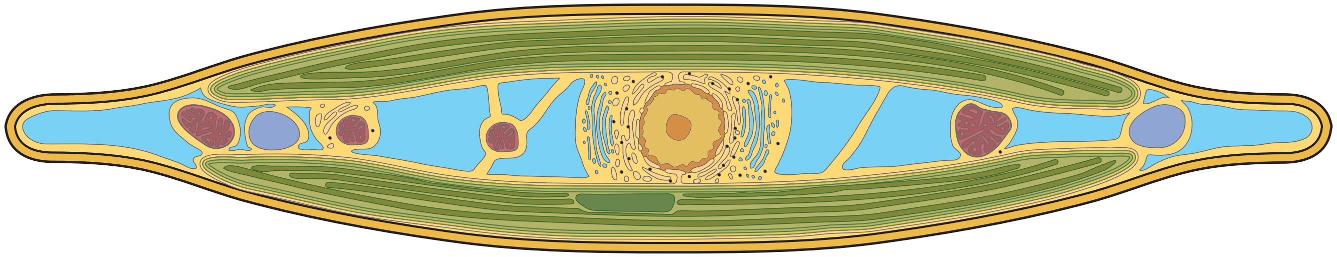

GIRDLE VIEW

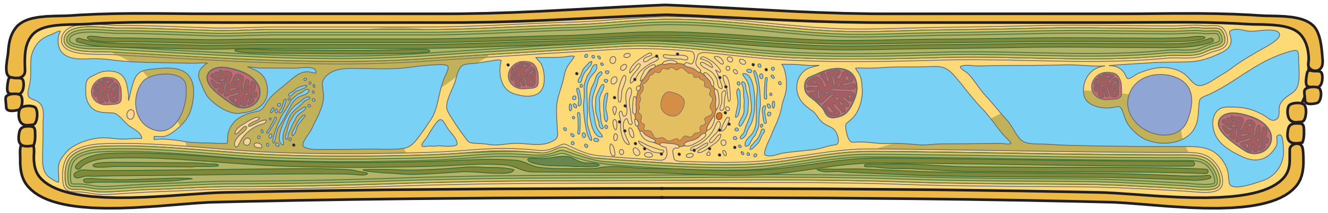

Supplement: S3 File — (PDF) [file pbio.3002395.s003.pdf]
