# Supplementary figures and images for: Openly available illustrations as tools to describe eukaryotic microbial diversity
Source: PLoS Biol. 2023 Nov 21;21(11):e3002395. doi: 10.1371/journal.pbio.3002395 (PMC10662721; doi:10.1371/journal.pbio.3002395)

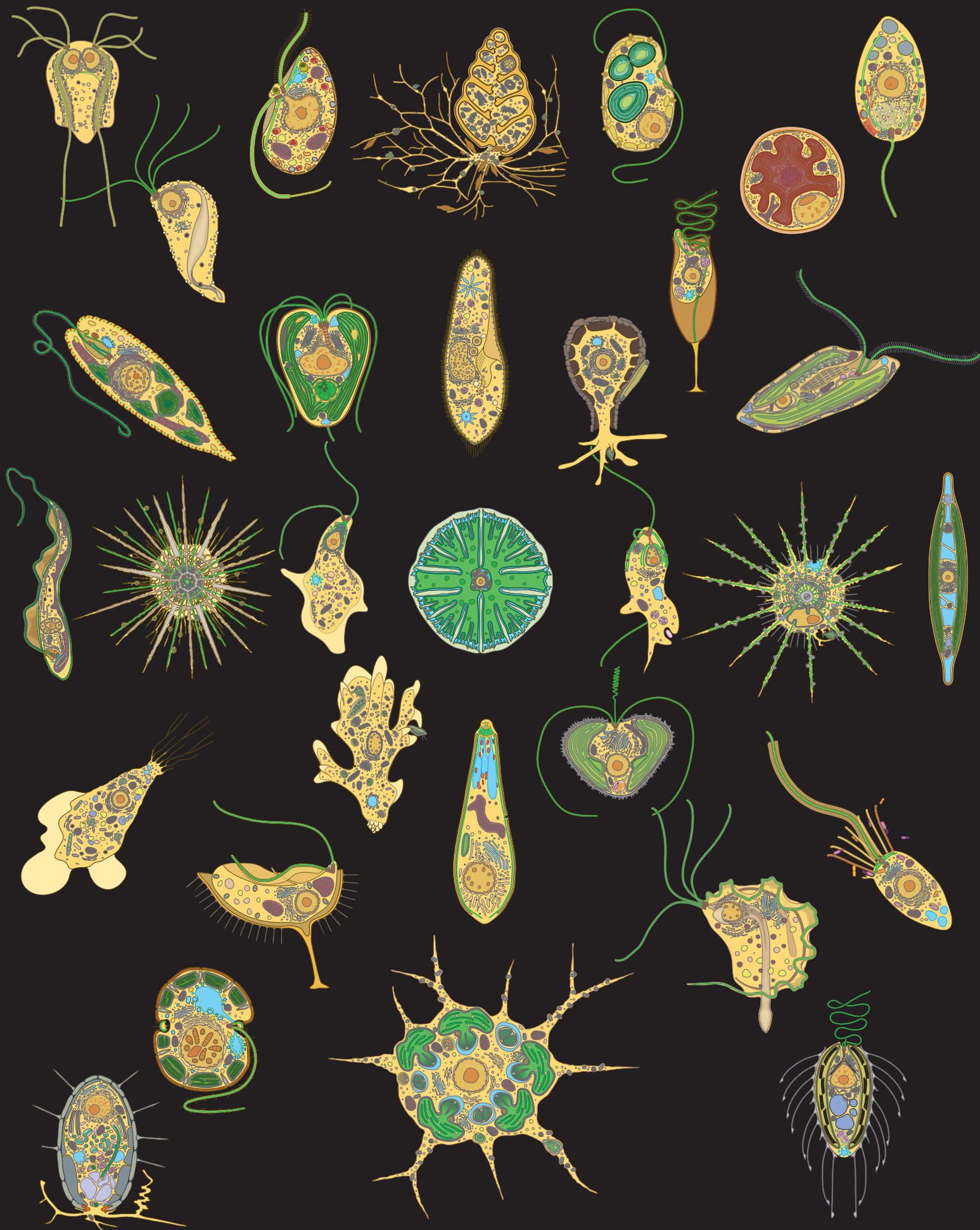

Supplement: S4 File — (PDF) [file pbio.3002395.s004.pdf]

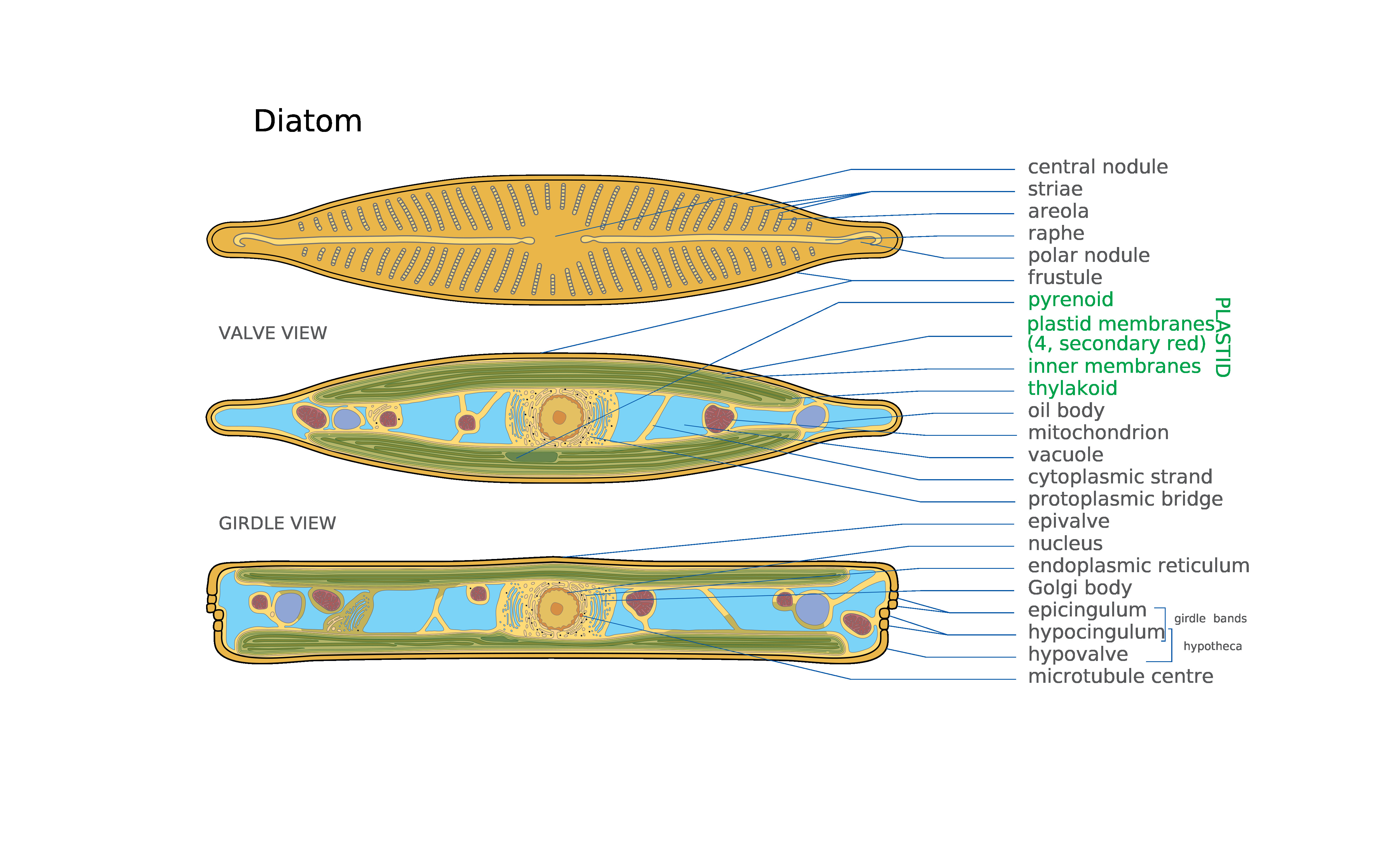

Supplement: S6 File — (ZIP) [file pbio.3002395.s006.zip › 2023 Pictures JPG files/2023 Diatom.jpg]

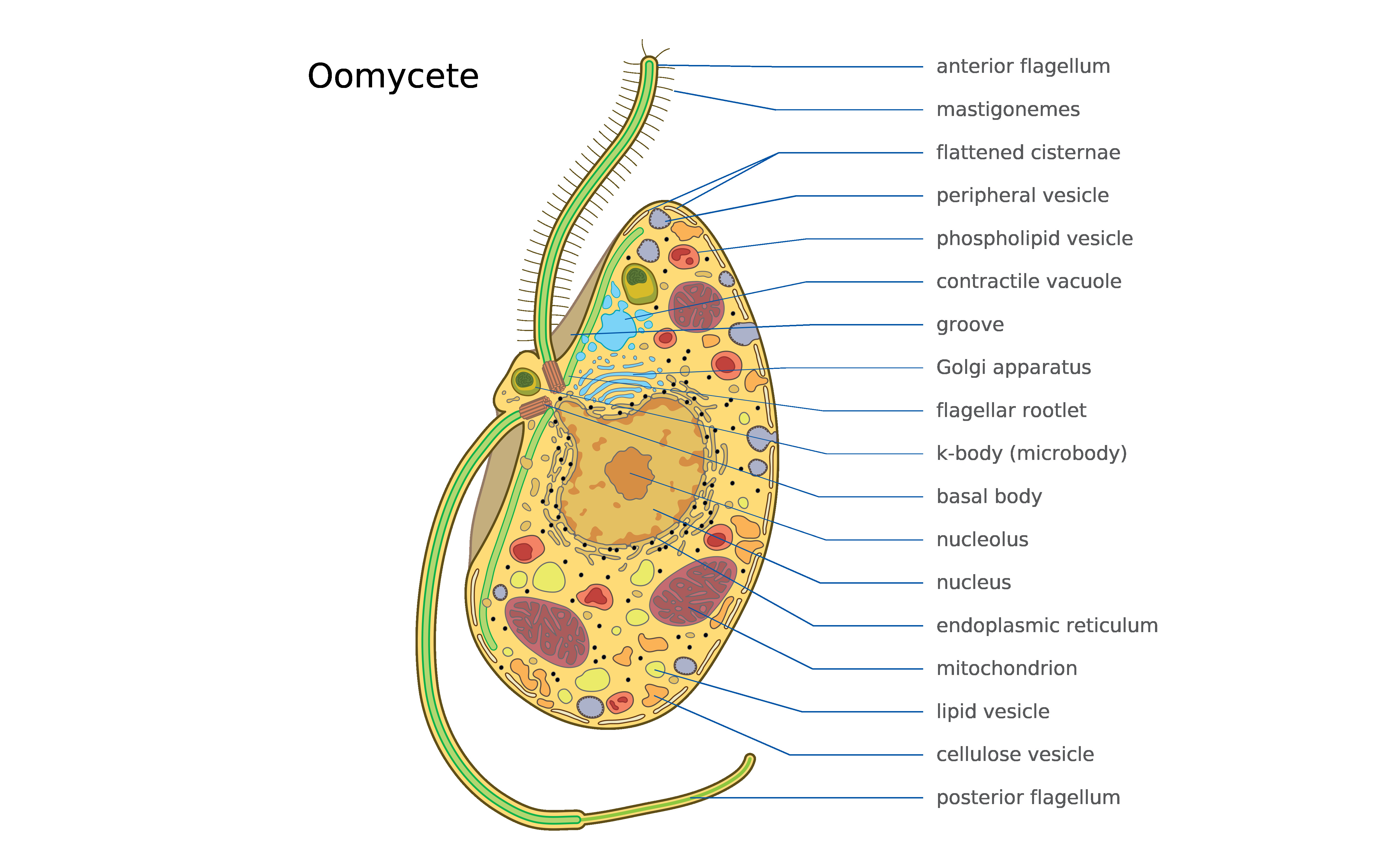

Supplement: S6 File — (ZIP) [file pbio.3002395.s006.zip › 2023 Pictures JPG files/2023 Oomycete.jpg]

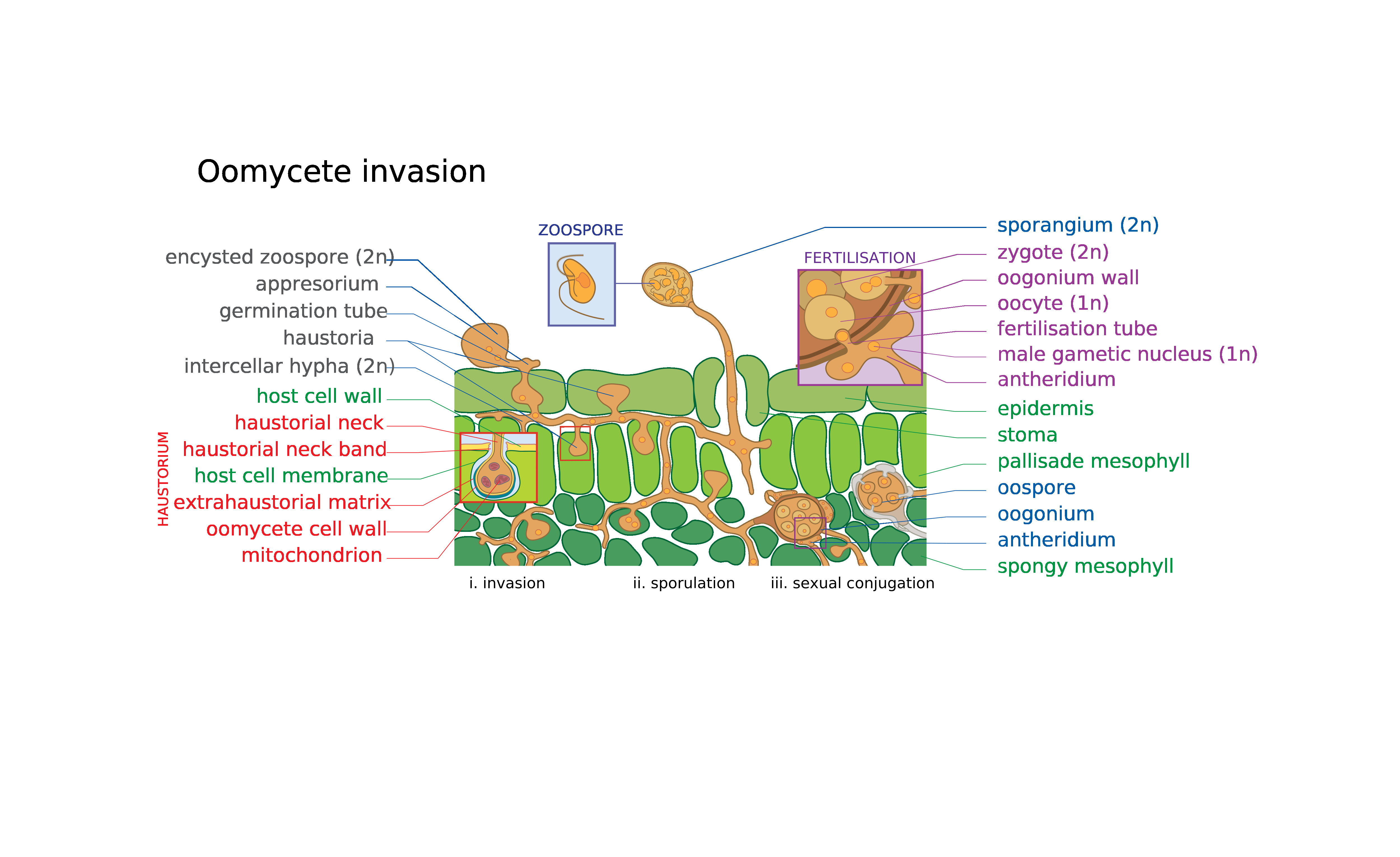

Supplement: S6 File — (ZIP) [file pbio.3002395.s006.zip › 2023 Pictures JPG files/2023 Oomycete Invasion.jpg]

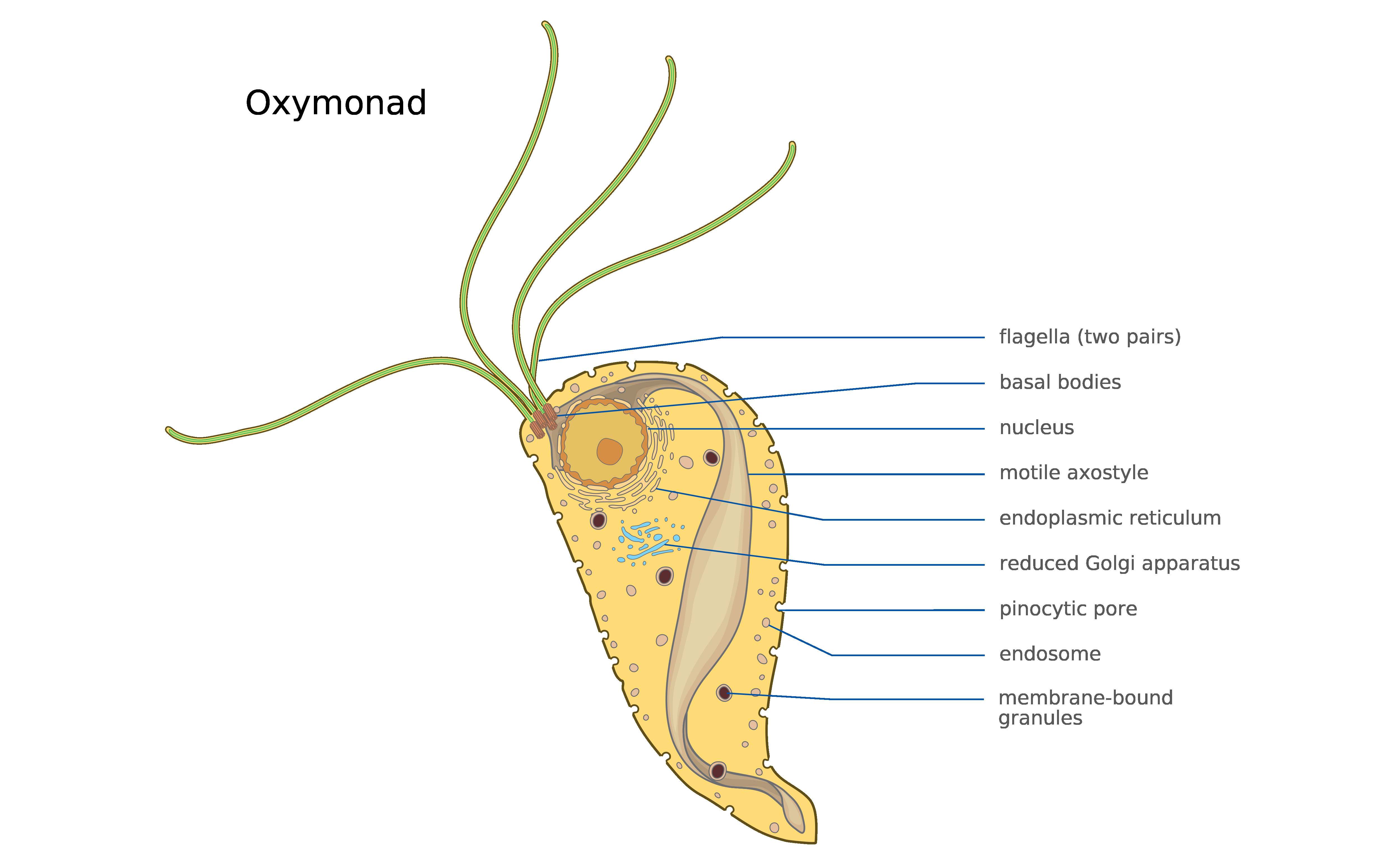

Supplement: S6 File — (ZIP) [file pbio.3002395.s006.zip › 2023 Pictures JPG files/2023 Oxymonad.jpg]

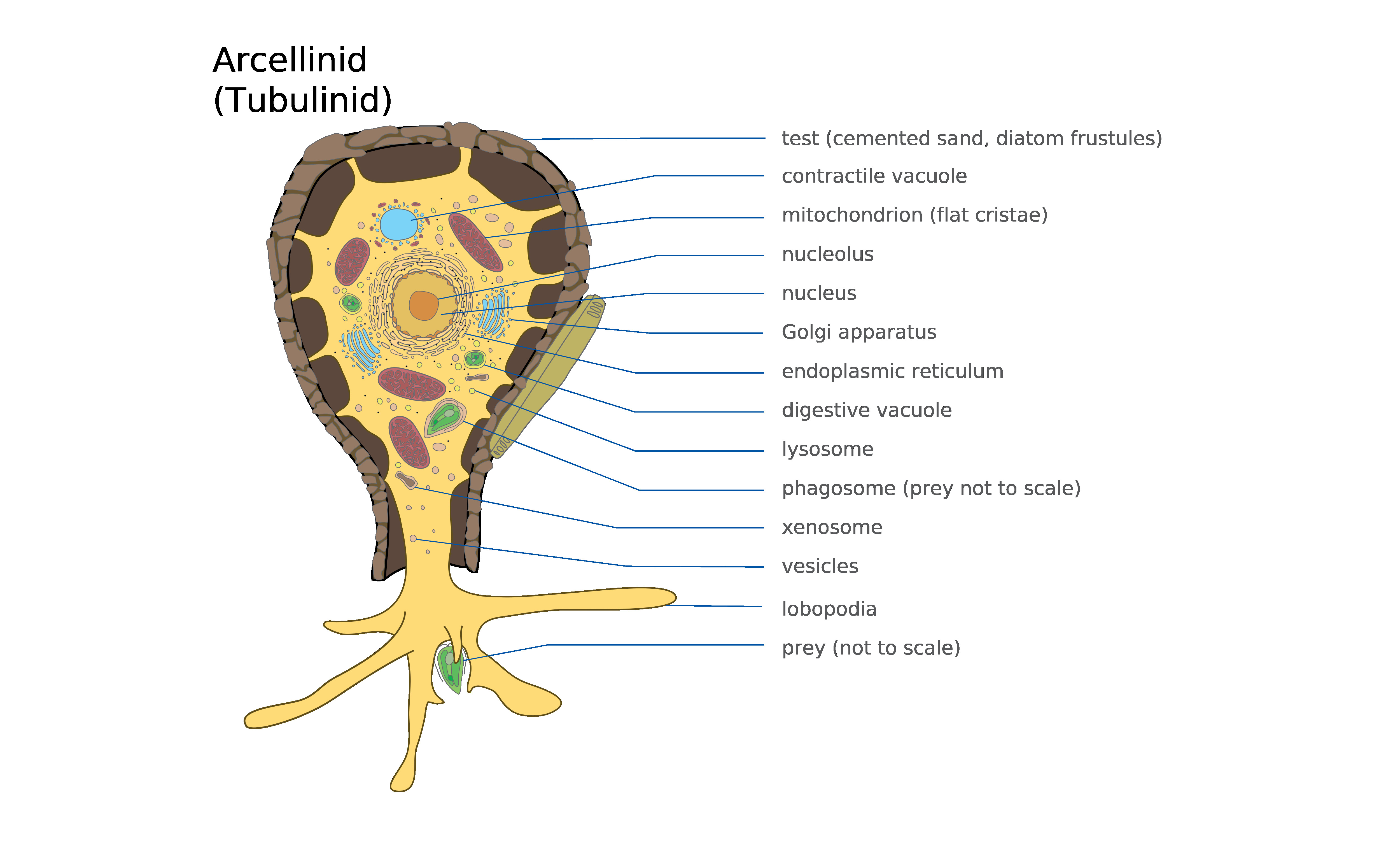

Supplement: S6 File — (ZIP) [file pbio.3002395.s006.zip › 2023 Pictures JPG files/2023 Arcellinid.jpg]

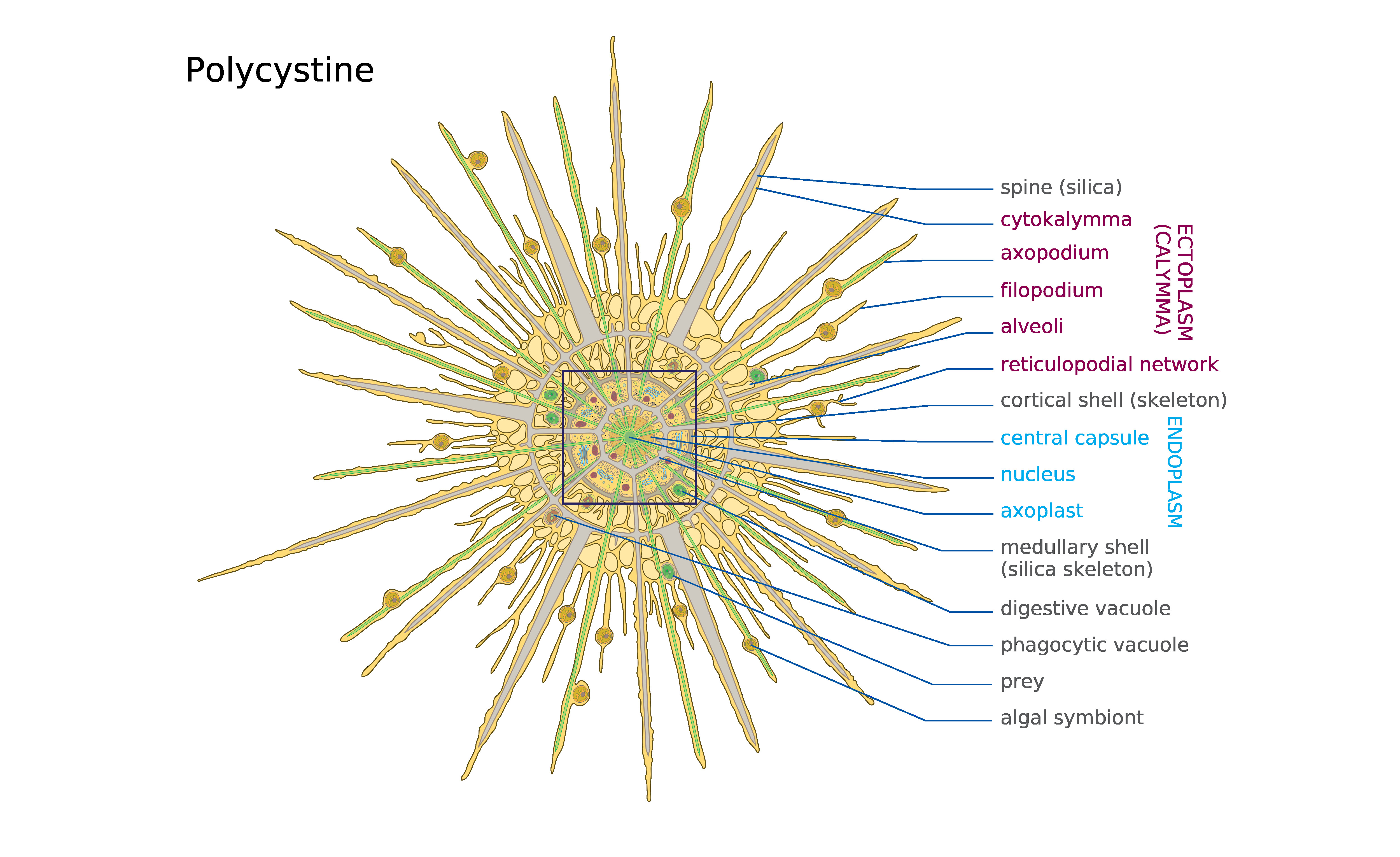

Supplement: S6 File — (ZIP) [file pbio.3002395.s006.zip › 2023 Pictures JPG files/2023 PolycystineWHOLE.jpg]

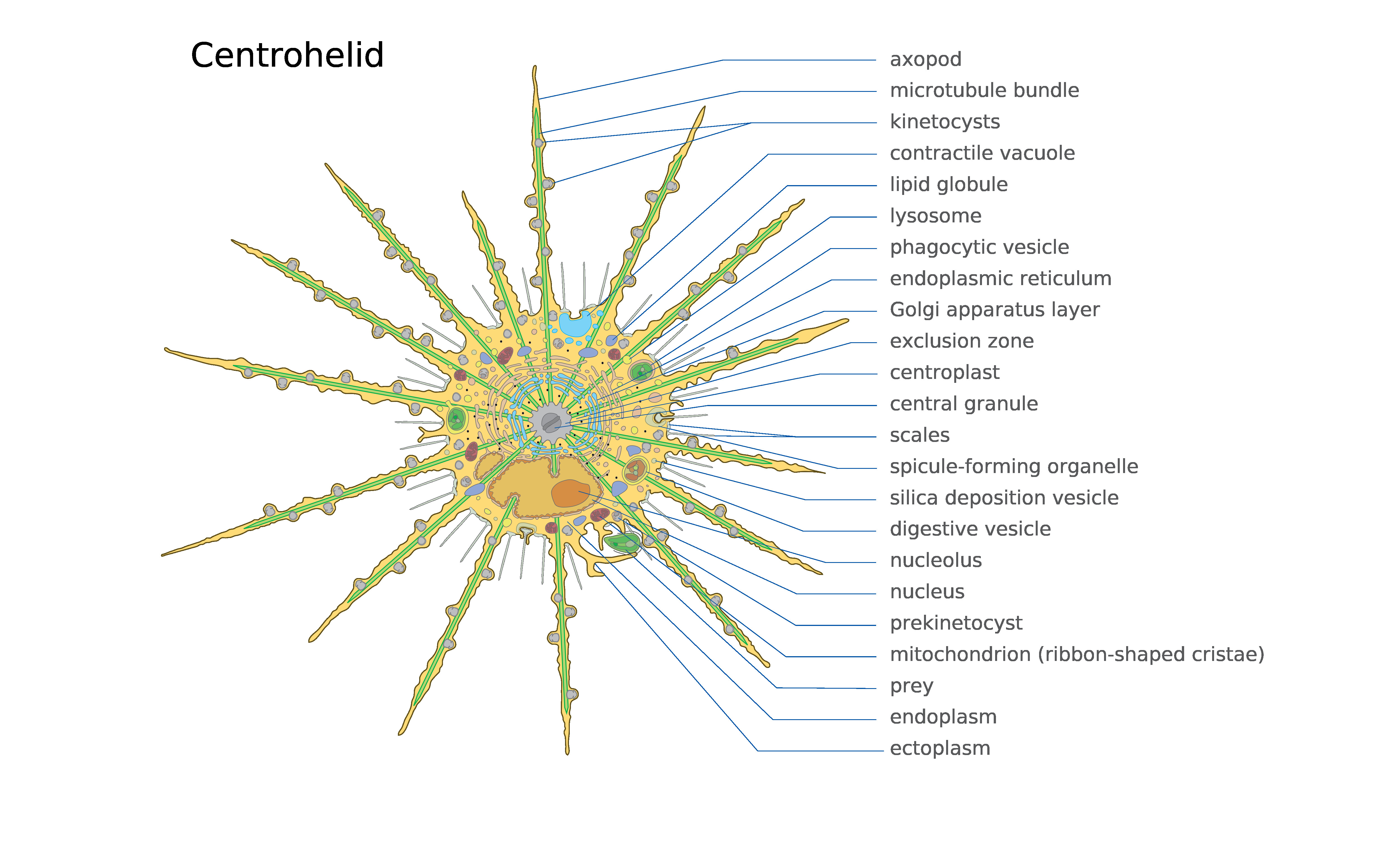

Supplement: S6 File — (ZIP) [file pbio.3002395.s006.zip › 2023 Pictures JPG files/2023 Centrohelid.jpg]

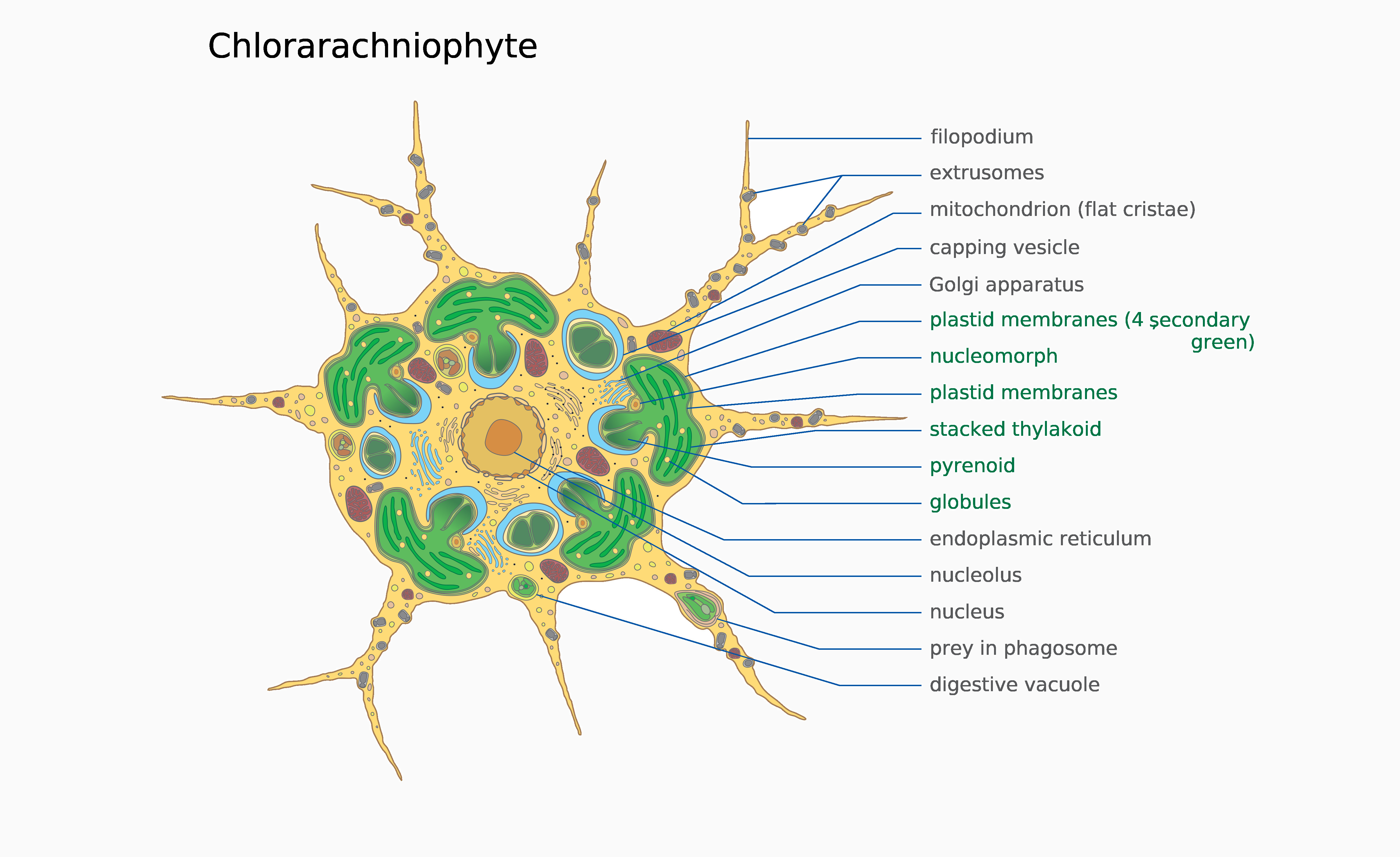

Supplement: S6 File — (ZIP) [file pbio.3002395.s006.zip › 2023 Pictures JPG files/2023 Chlorarachniophyte.jpg]

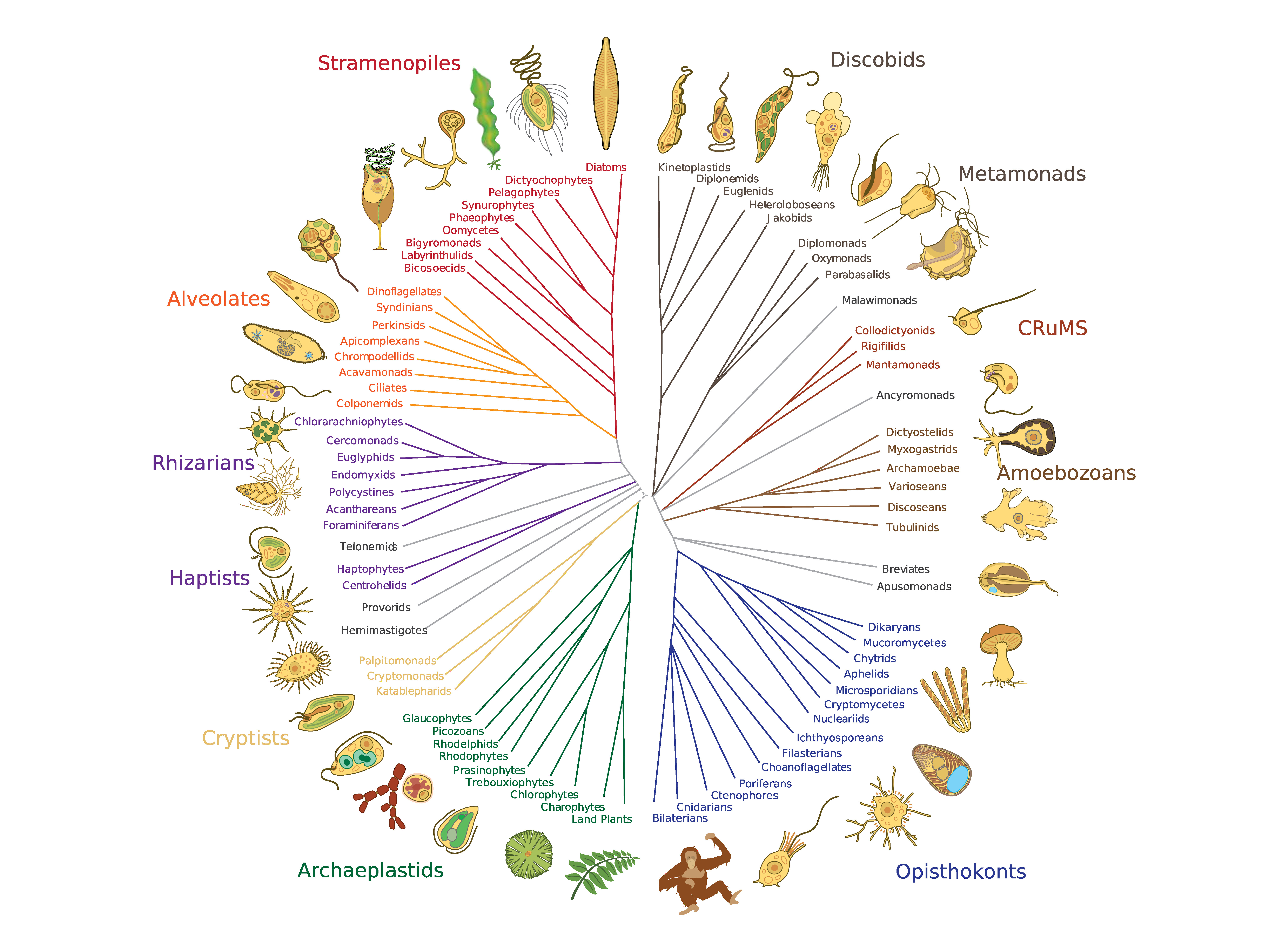

Supplement: S6 File — (ZIP) [file pbio.3002395.s006.zip › 2023 Pictures JPG files/2023 Figure 1 Tree.jpg]

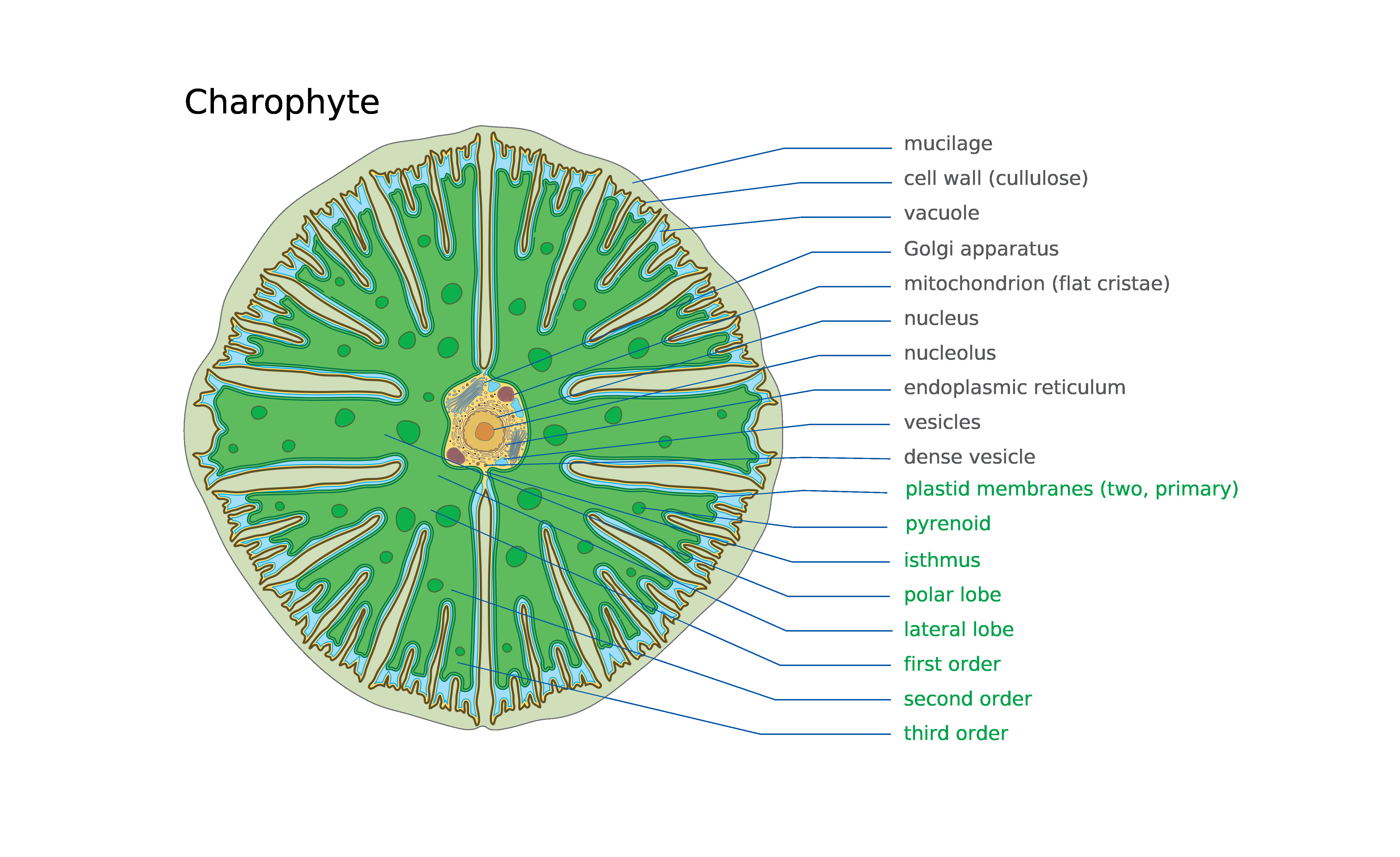

Supplement: S6 File — (ZIP) [file pbio.3002395.s006.zip › 2023 Pictures JPG files/2023 Charophyte.jpg]

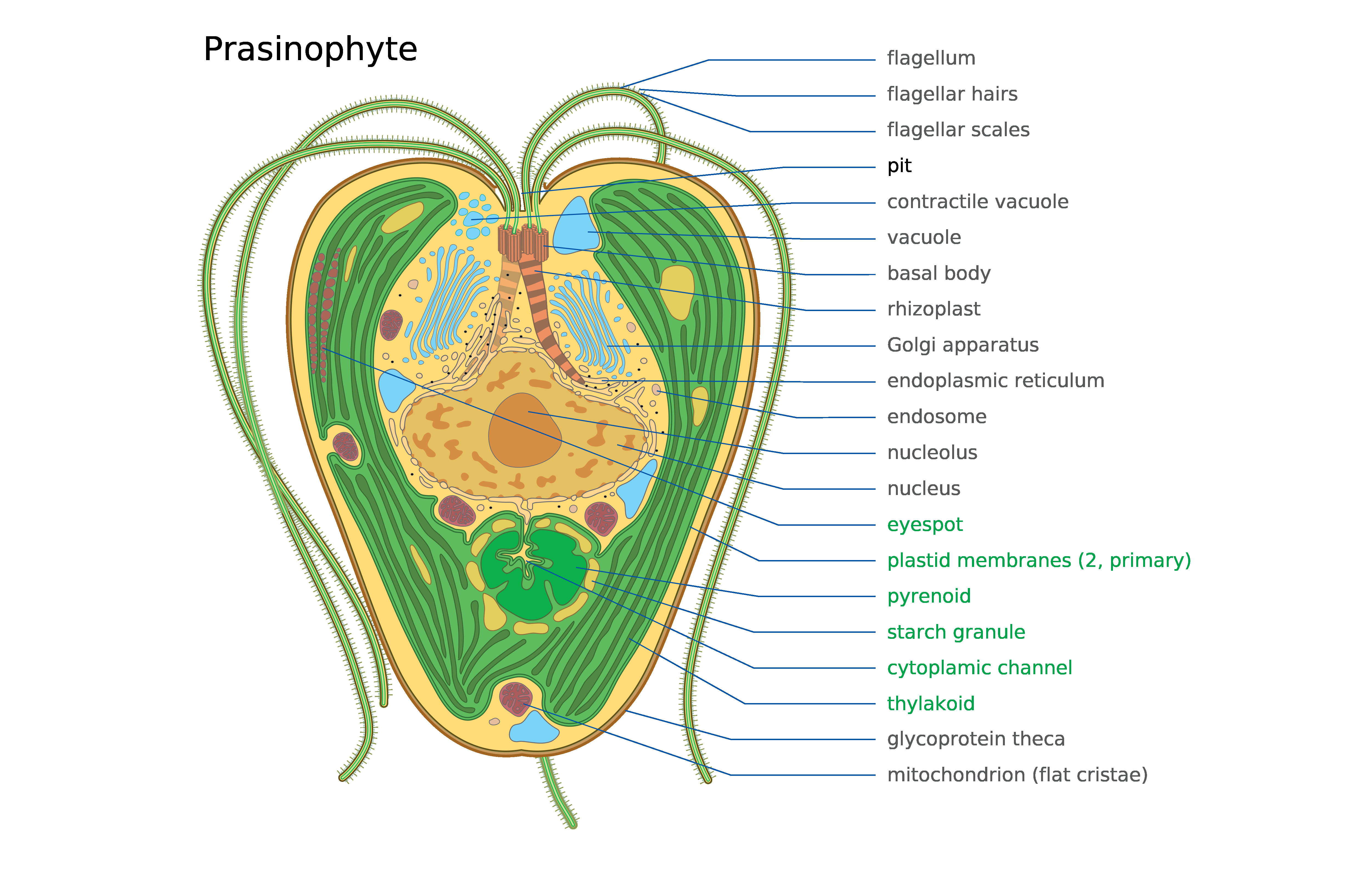

Supplement: S6 File — (ZIP) [file pbio.3002395.s006.zip › 2023 Pictures JPG files/2023 Prasinophyte.jpg]

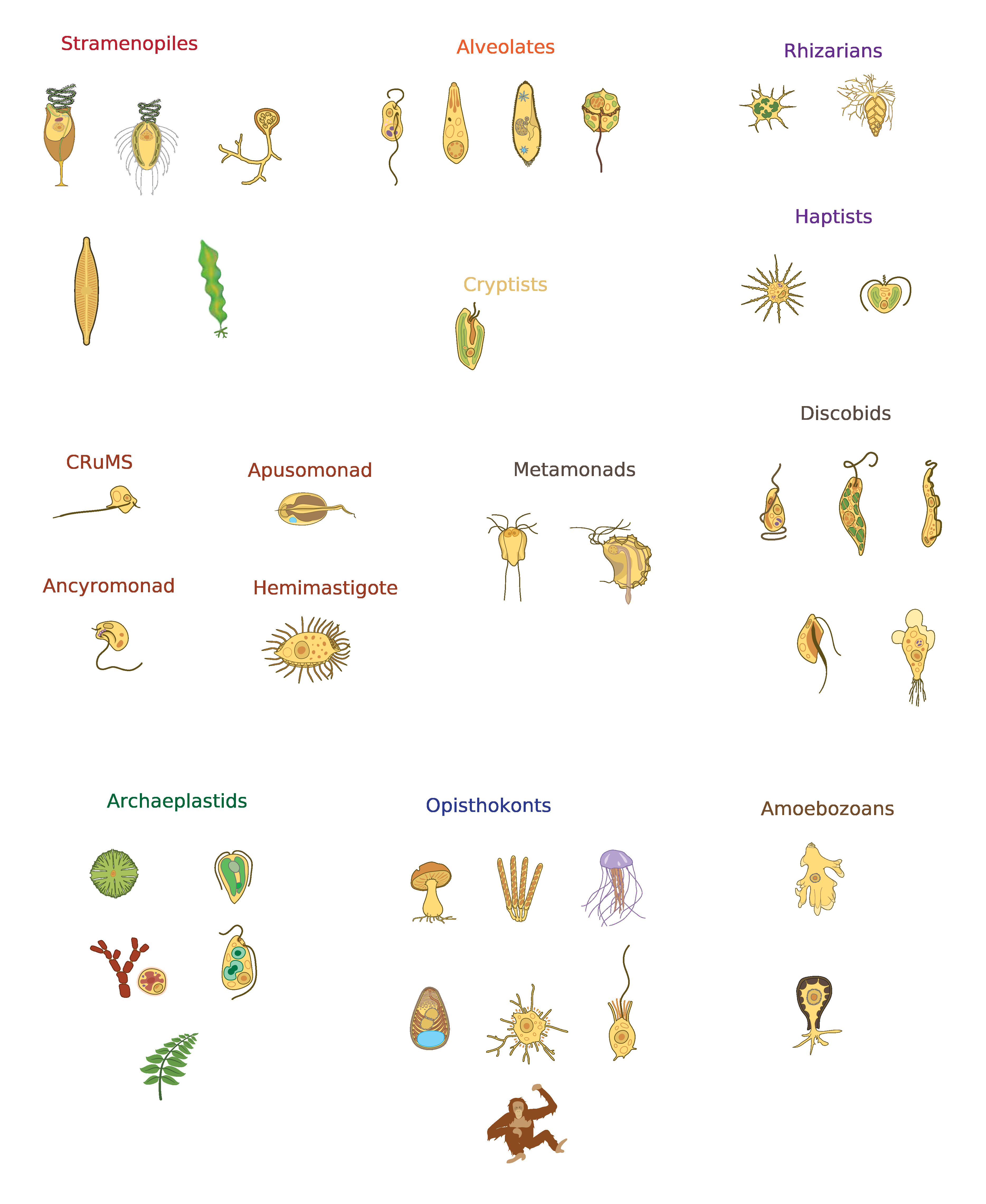

Supplement: S6 File — (ZIP) [file pbio.3002395.s006.zip › 2023 Pictures JPG files/2023 Figure S3 Thumbnails.jpg]

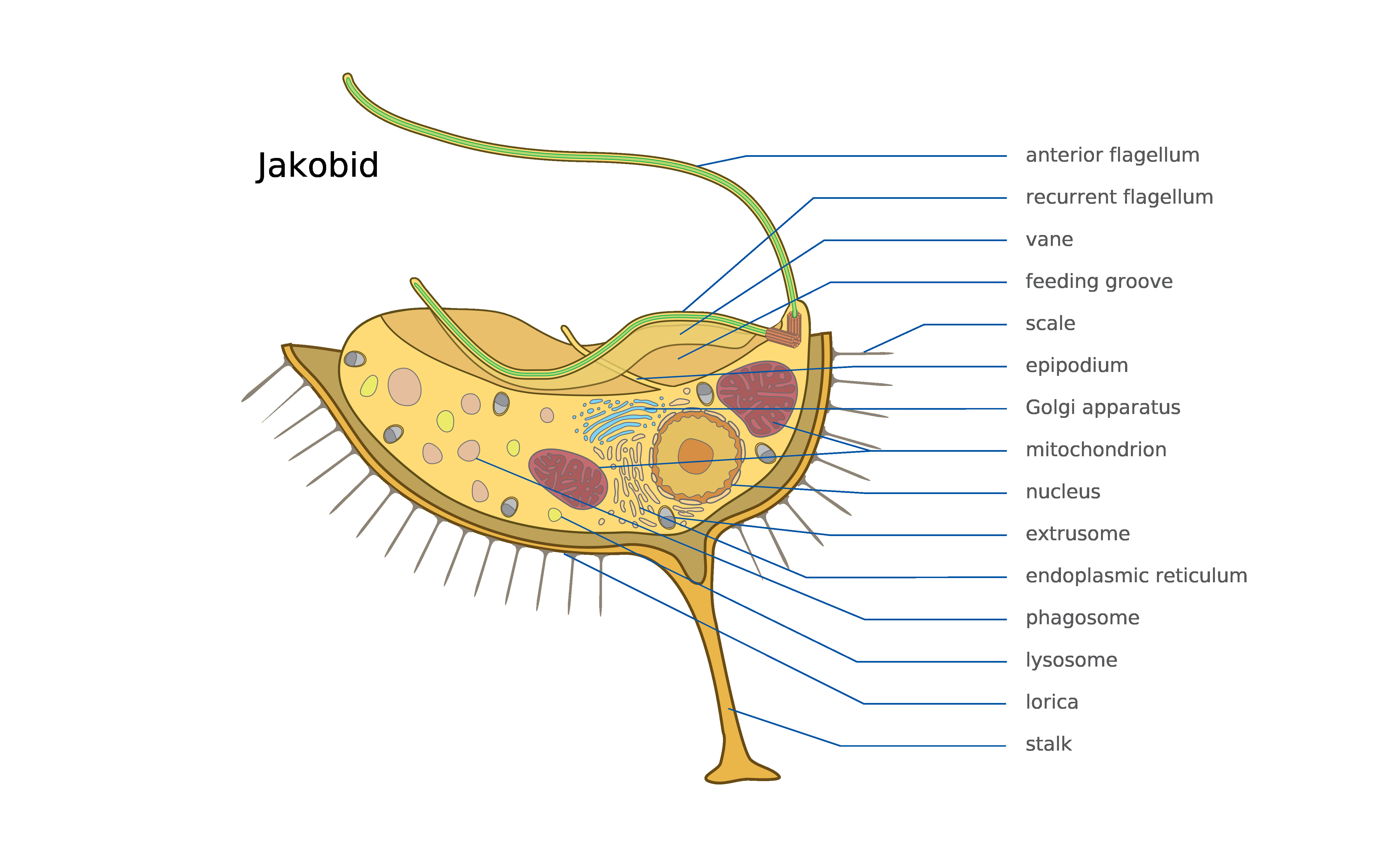

Supplement: S6 File — (ZIP) [file pbio.3002395.s006.zip › 2023 Pictures JPG files/2023 Jakobid.jpg]

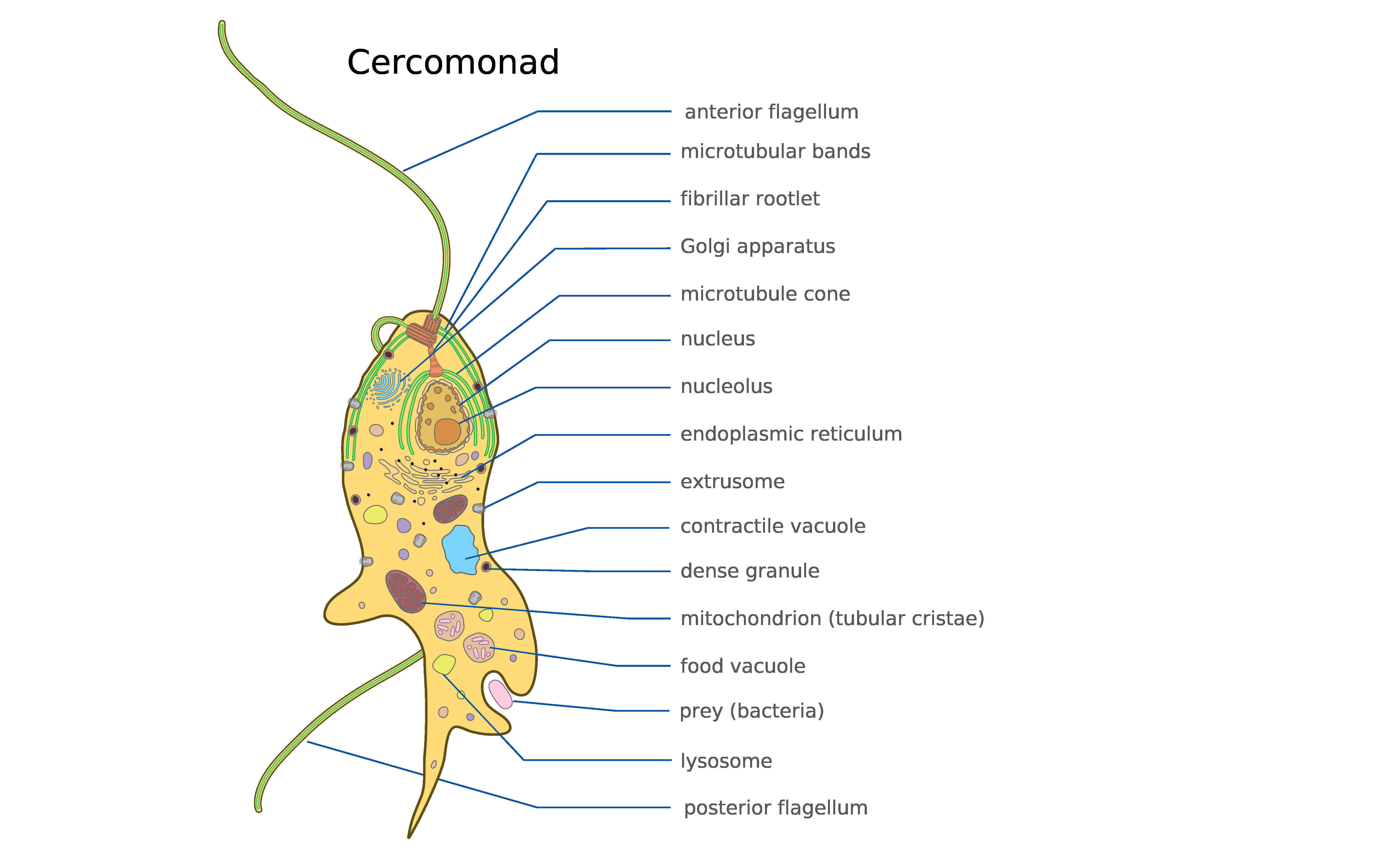

Supplement: S6 File — (ZIP) [file pbio.3002395.s006.zip › 2023 Pictures JPG files/2023 Cercomonad.jpg]

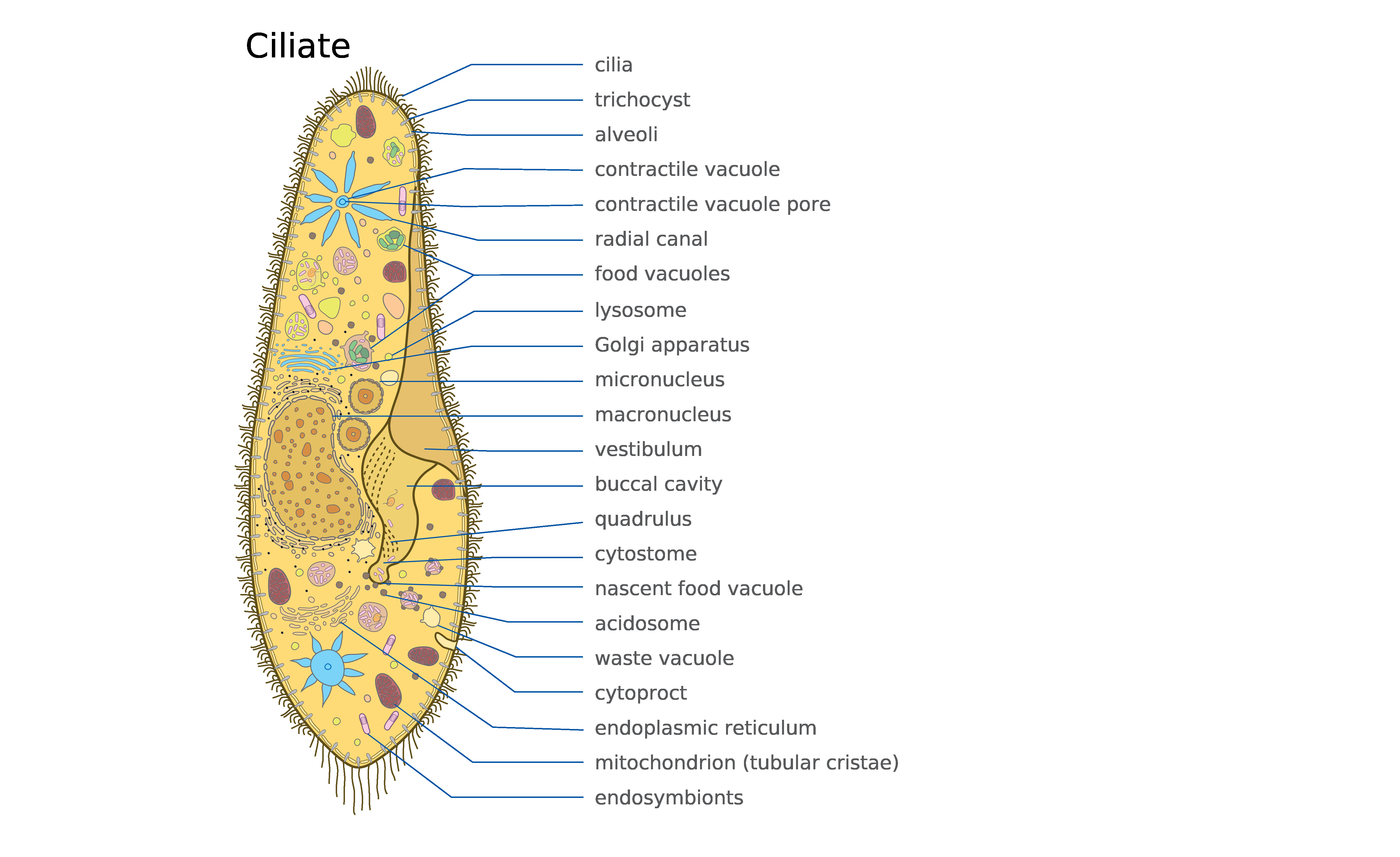

Supplement: S6 File — (ZIP) [file pbio.3002395.s006.zip › 2023 Pictures JPG files/2023 Ciliate.jpg]

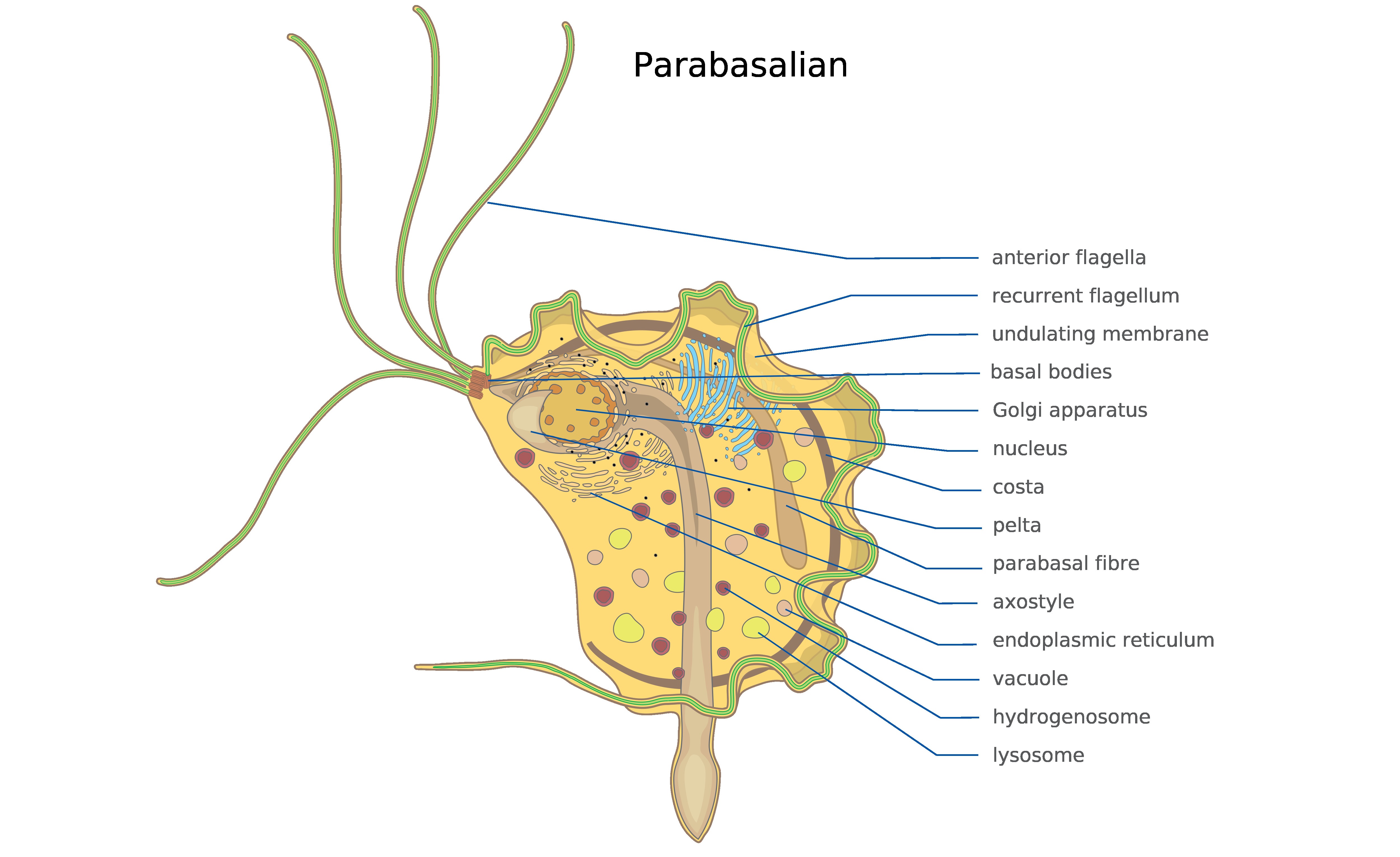

Supplement: S6 File — (ZIP) [file pbio.3002395.s006.zip › 2023 Pictures JPG files/2023 Parabasalian.jpg]

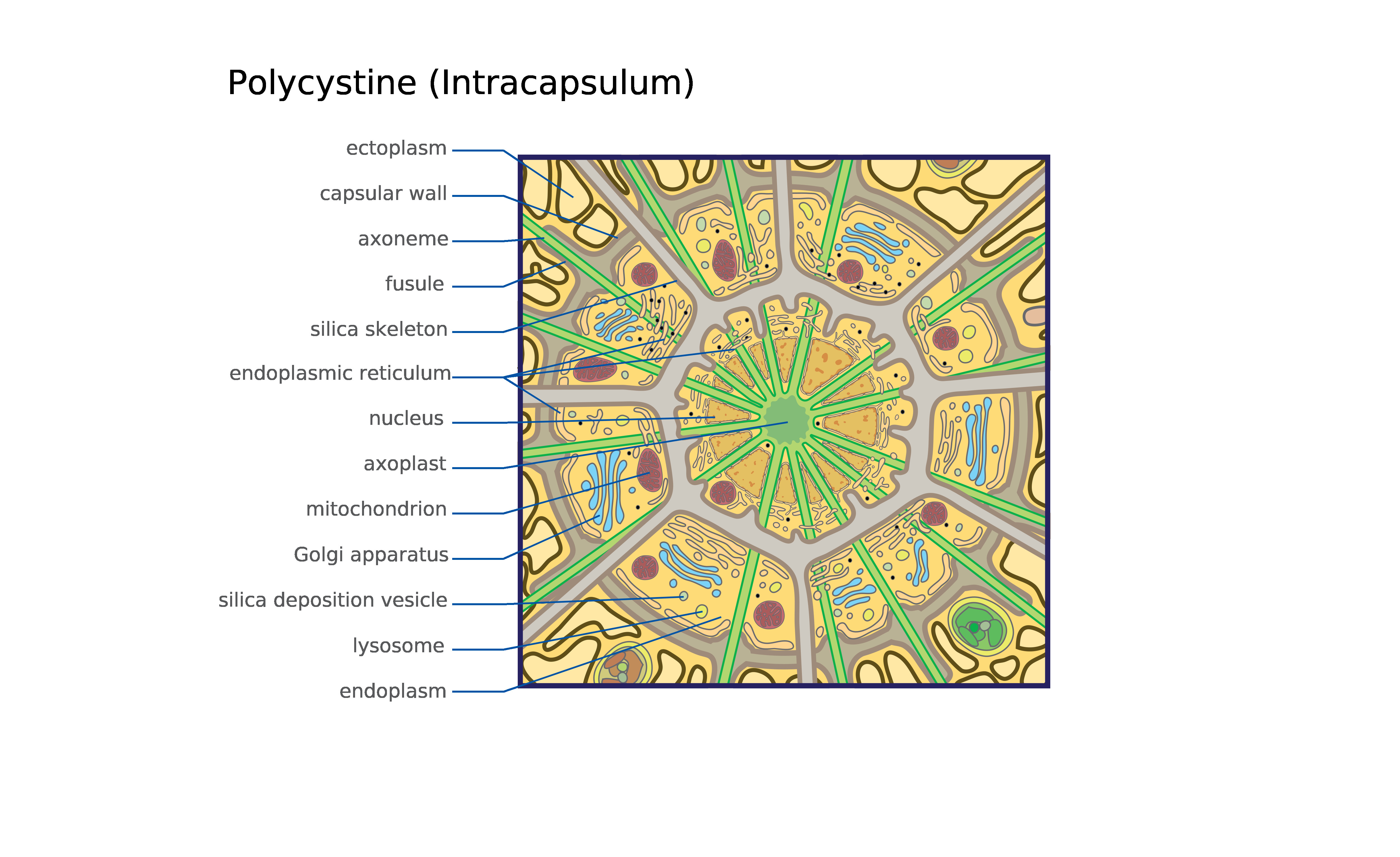

Supplement: S6 File — (ZIP) [file pbio.3002395.s006.zip › 2023 Pictures JPG files/2023 PolycystineINTRA.jpg]

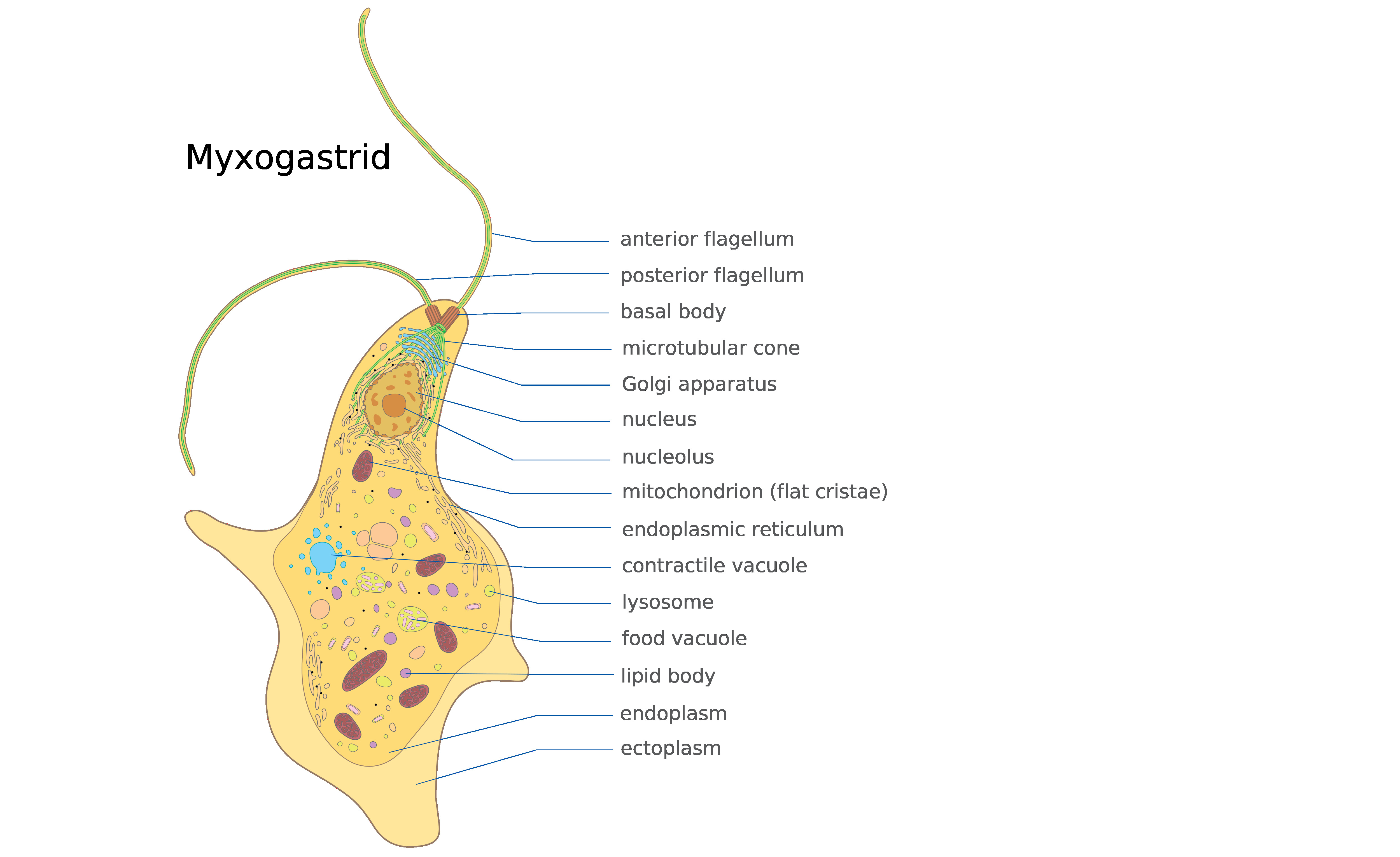

Supplement: S6 File — (ZIP) [file pbio.3002395.s006.zip › 2023 Pictures JPG files/2023 Myxogastrid.jpg]

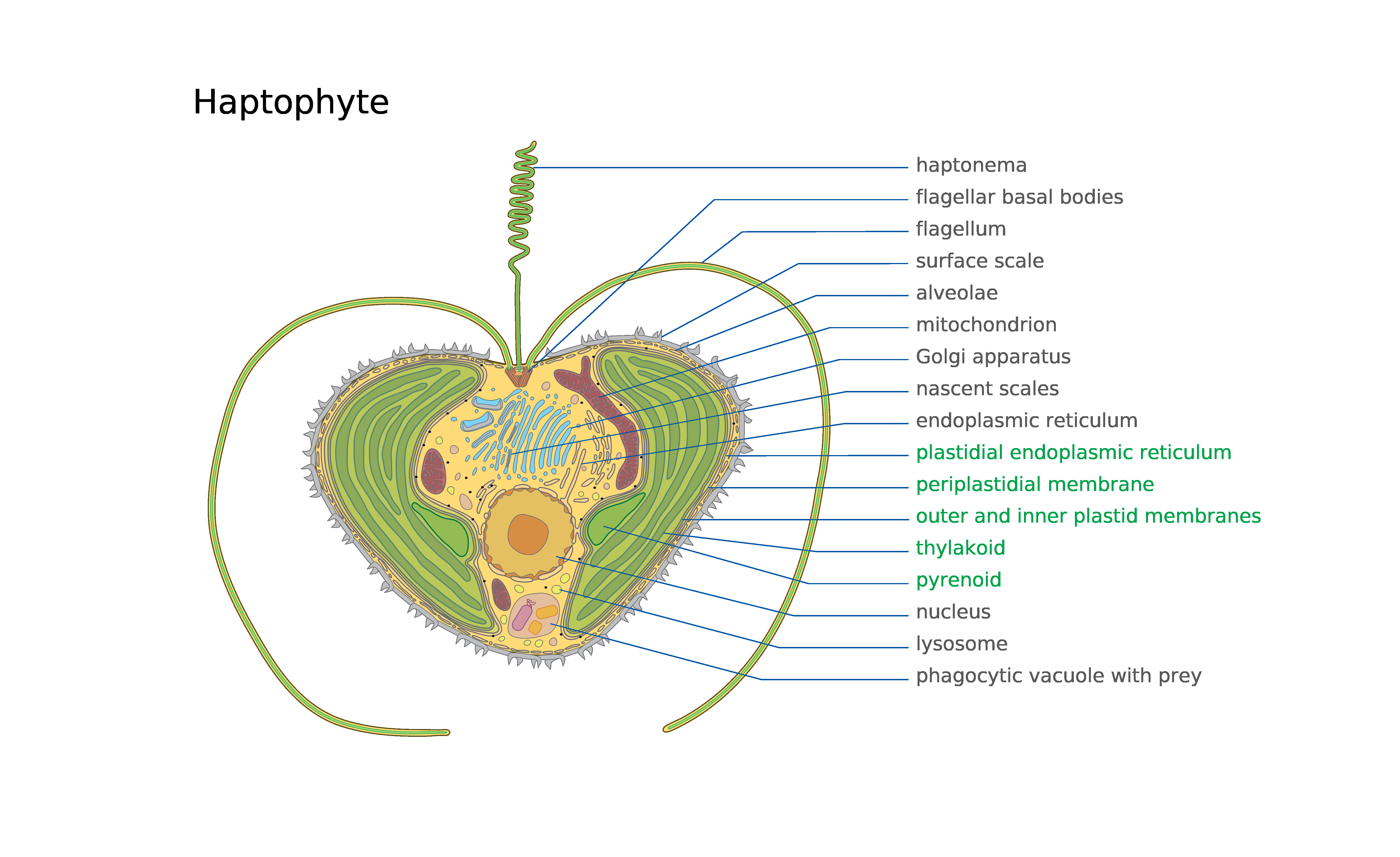

Supplement: S6 File — (ZIP) [file pbio.3002395.s006.zip › 2023 Pictures JPG files/2023 Haptophyte.jpg]

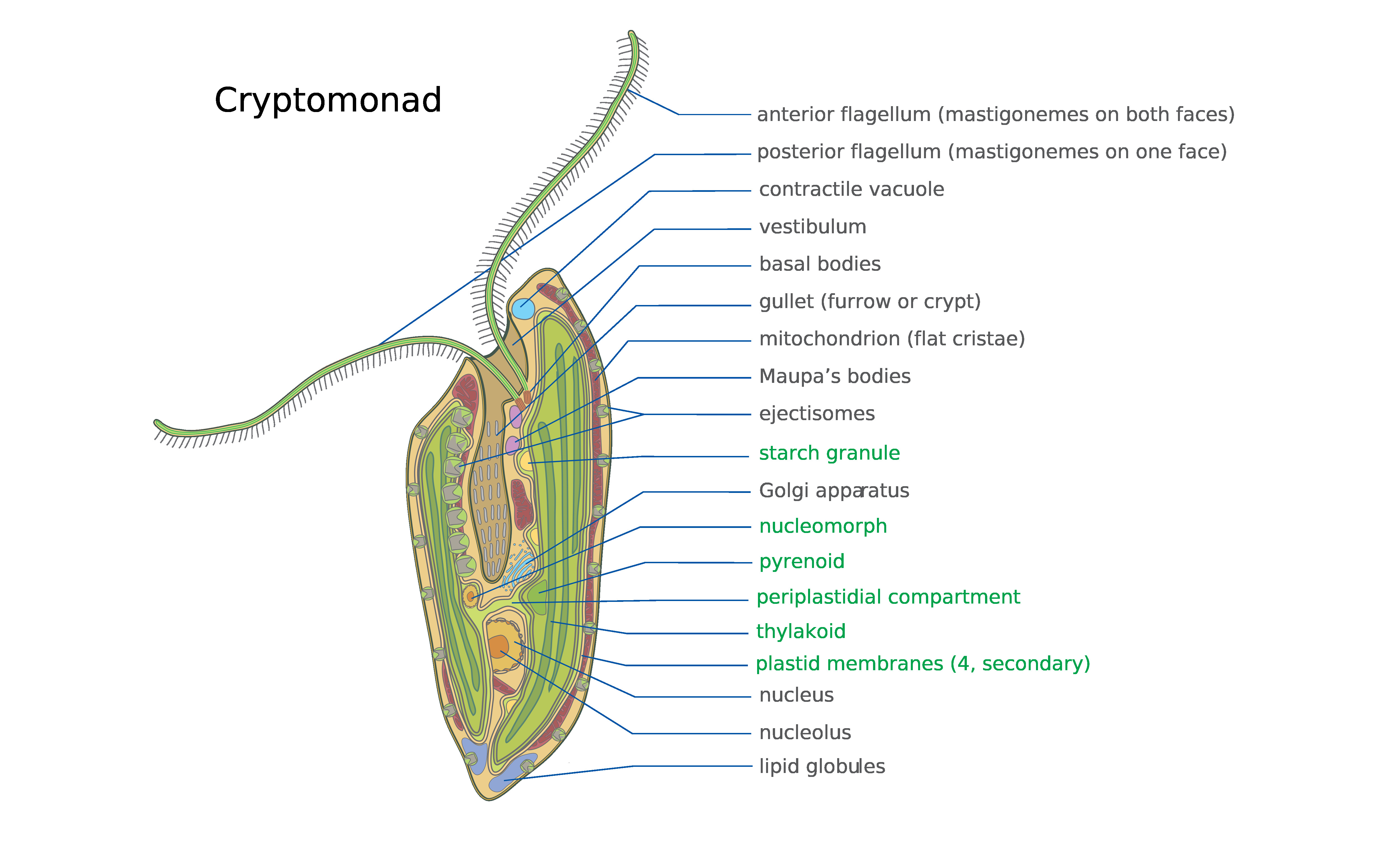

Supplement: S6 File — (ZIP) [file pbio.3002395.s006.zip › 2023 Pictures JPG files/2023 Cryptomonad.jpg]

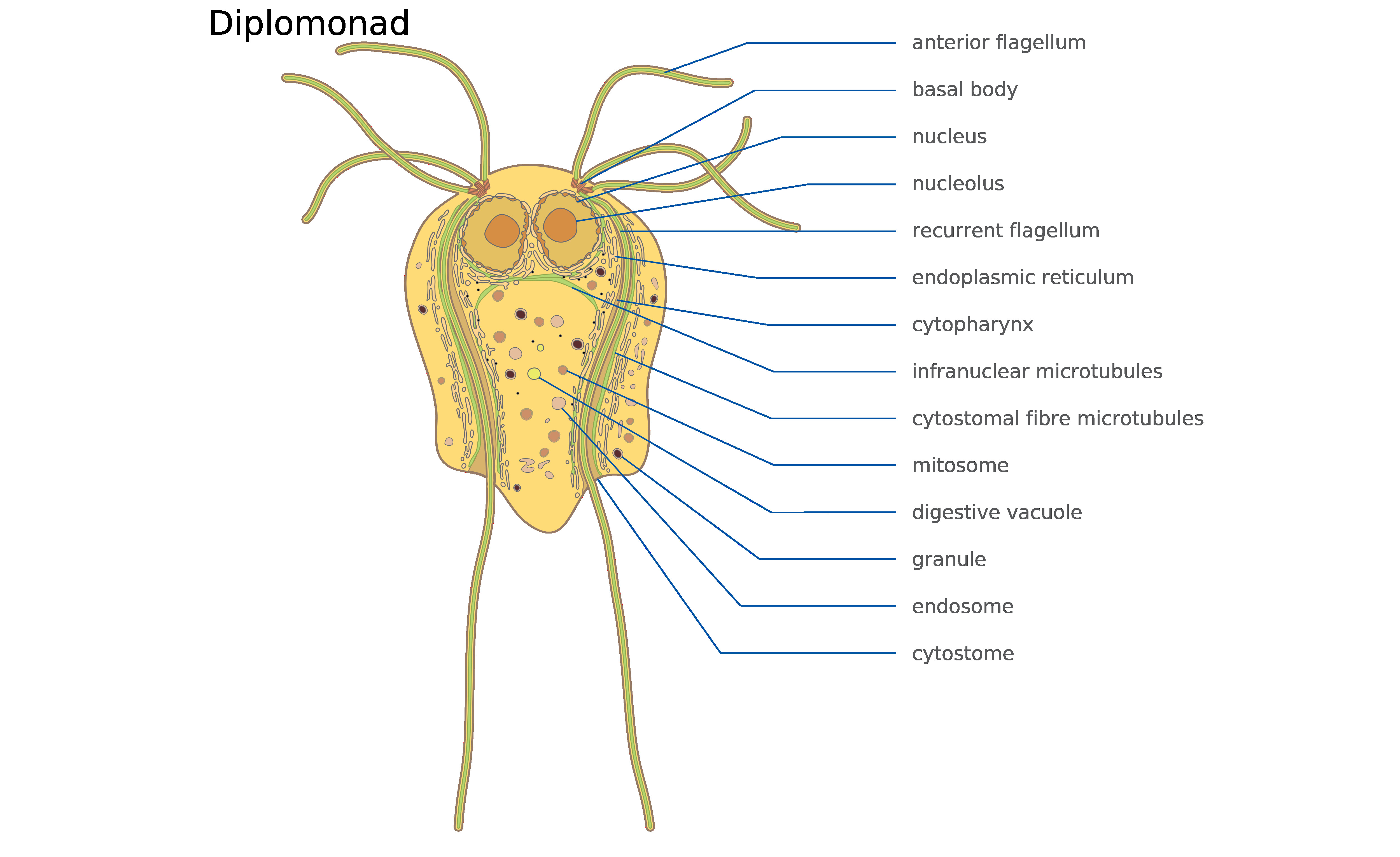

Supplement: S6 File — (ZIP) [file pbio.3002395.s006.zip › 2023 Pictures JPG files/2023 Diplomonad.jpg]

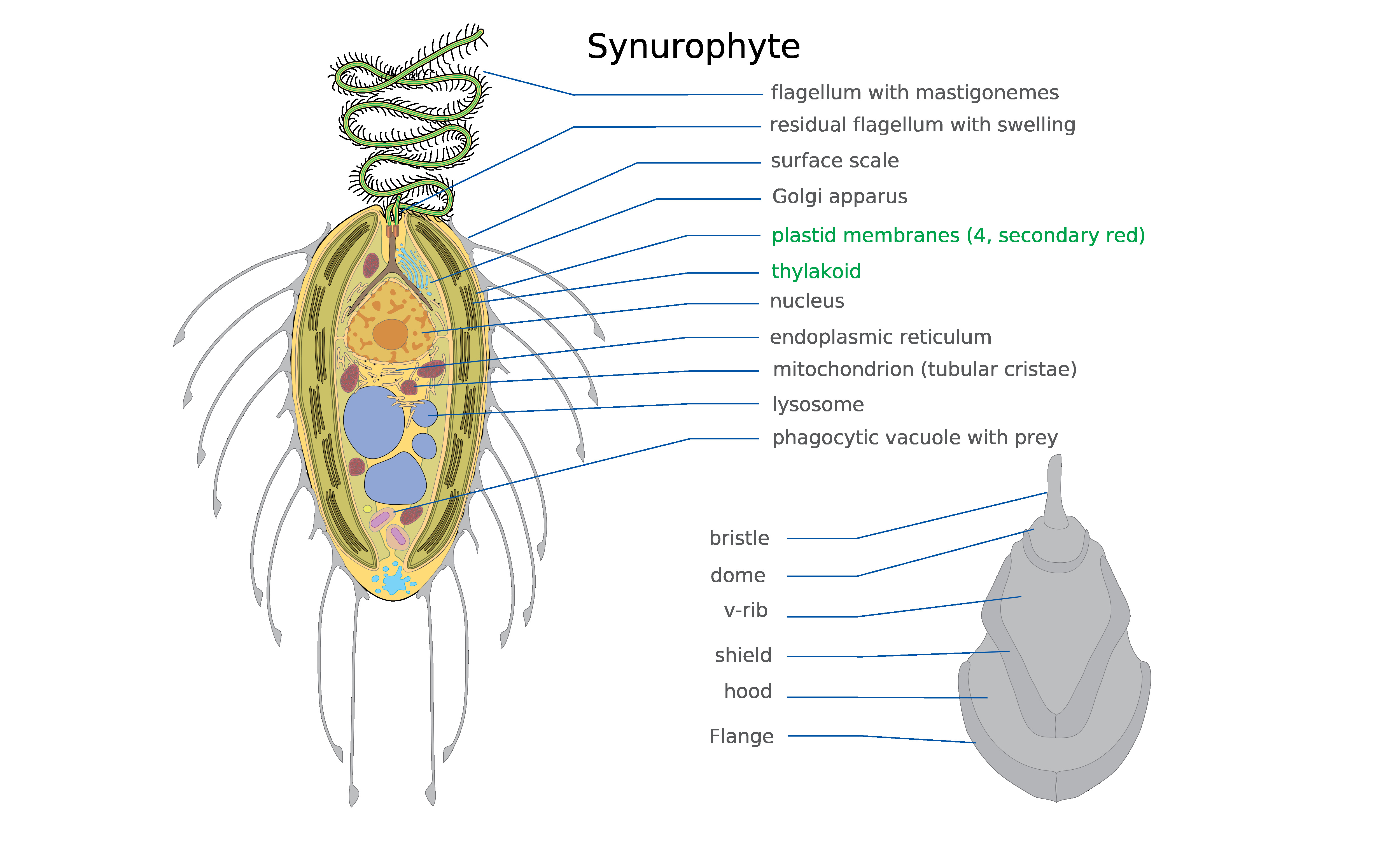

Supplement: S6 File — (ZIP) [file pbio.3002395.s006.zip › 2023 Pictures JPG files/2023 Synurophyte.jpg]

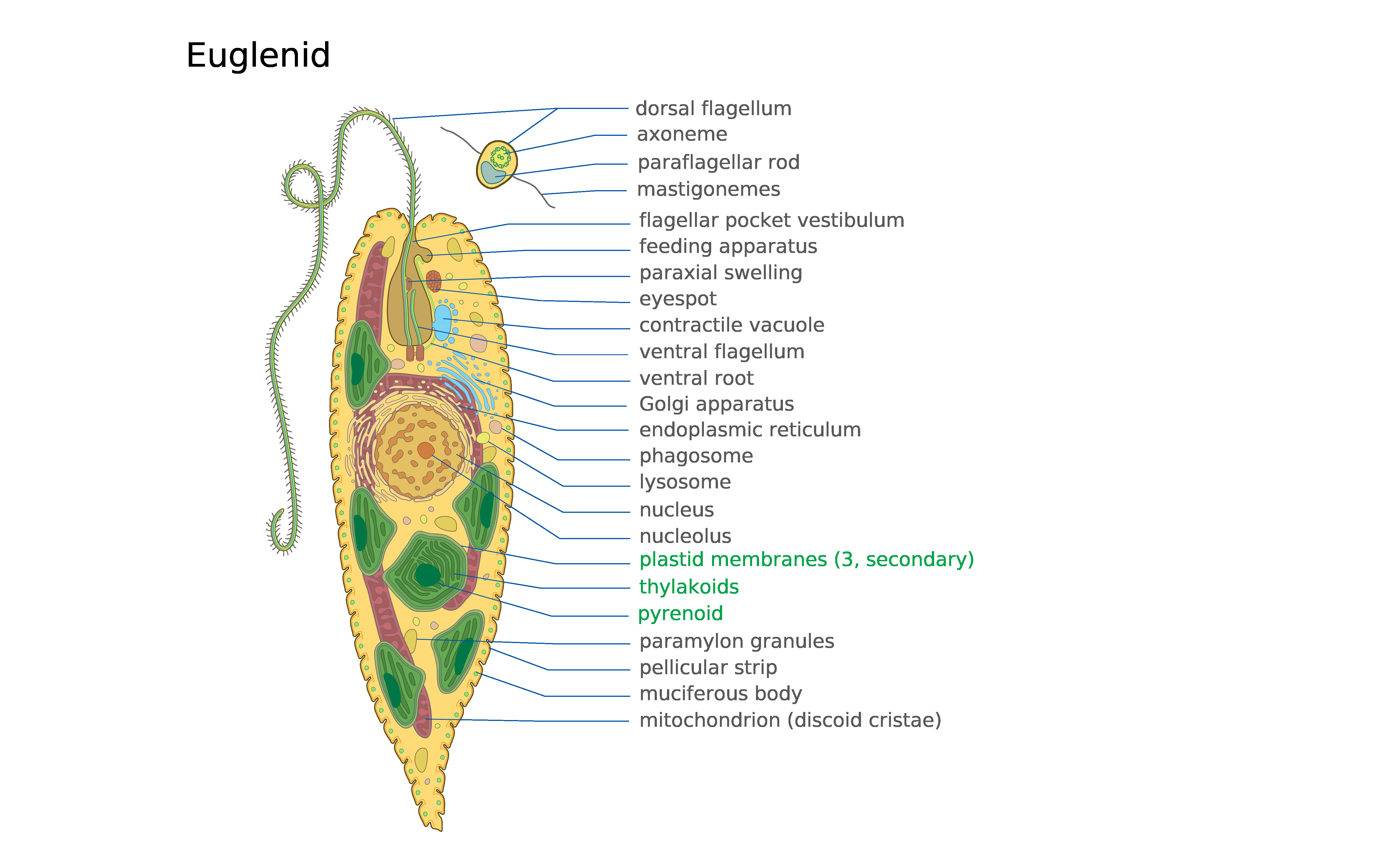

Supplement: S6 File — (ZIP) [file pbio.3002395.s006.zip › 2023 Pictures JPG files/2023 Eugenid.jpg]

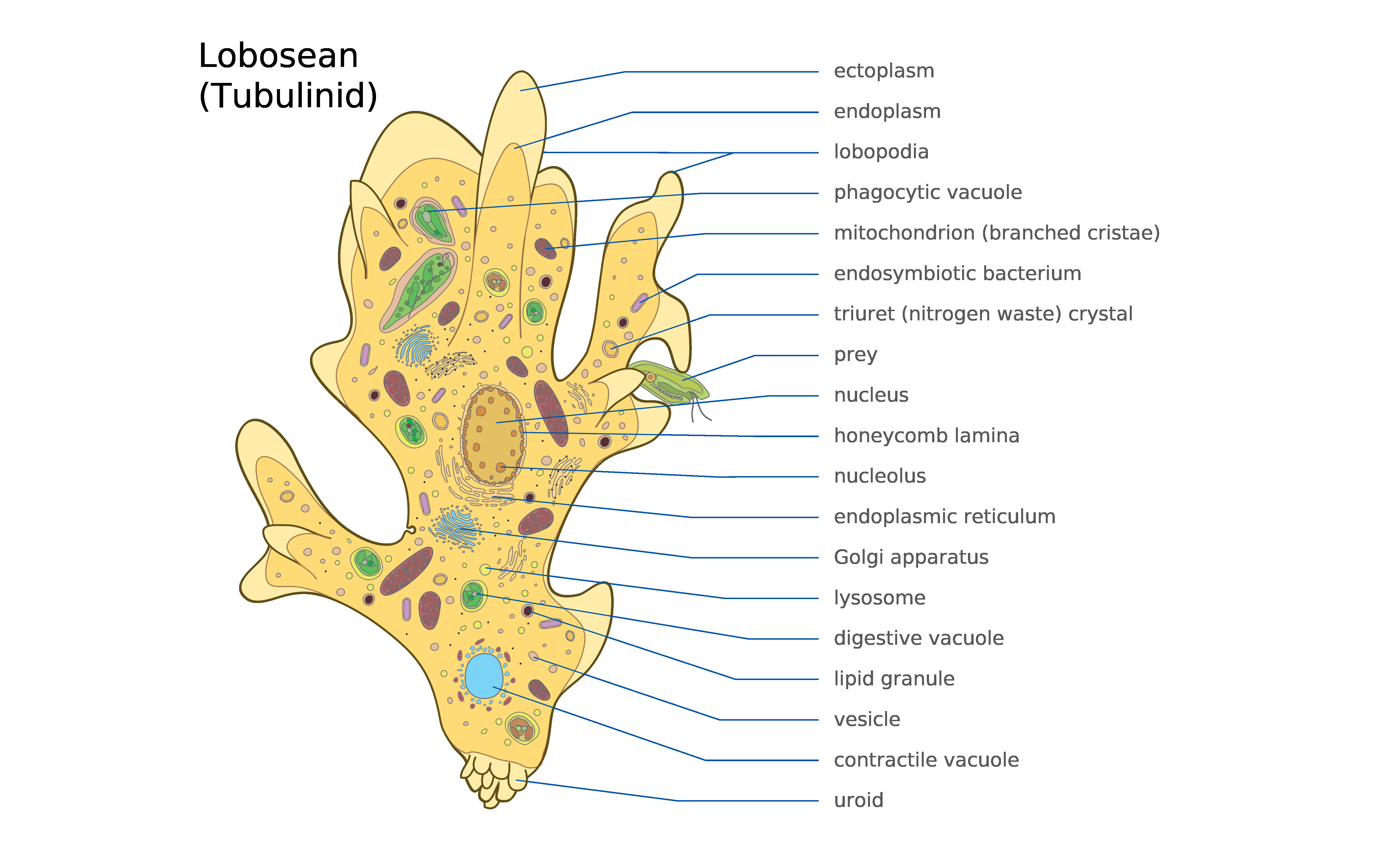

Supplement: S6 File — (ZIP) [file pbio.3002395.s006.zip › 2023 Pictures JPG files/2023 Lobosean.jpg]

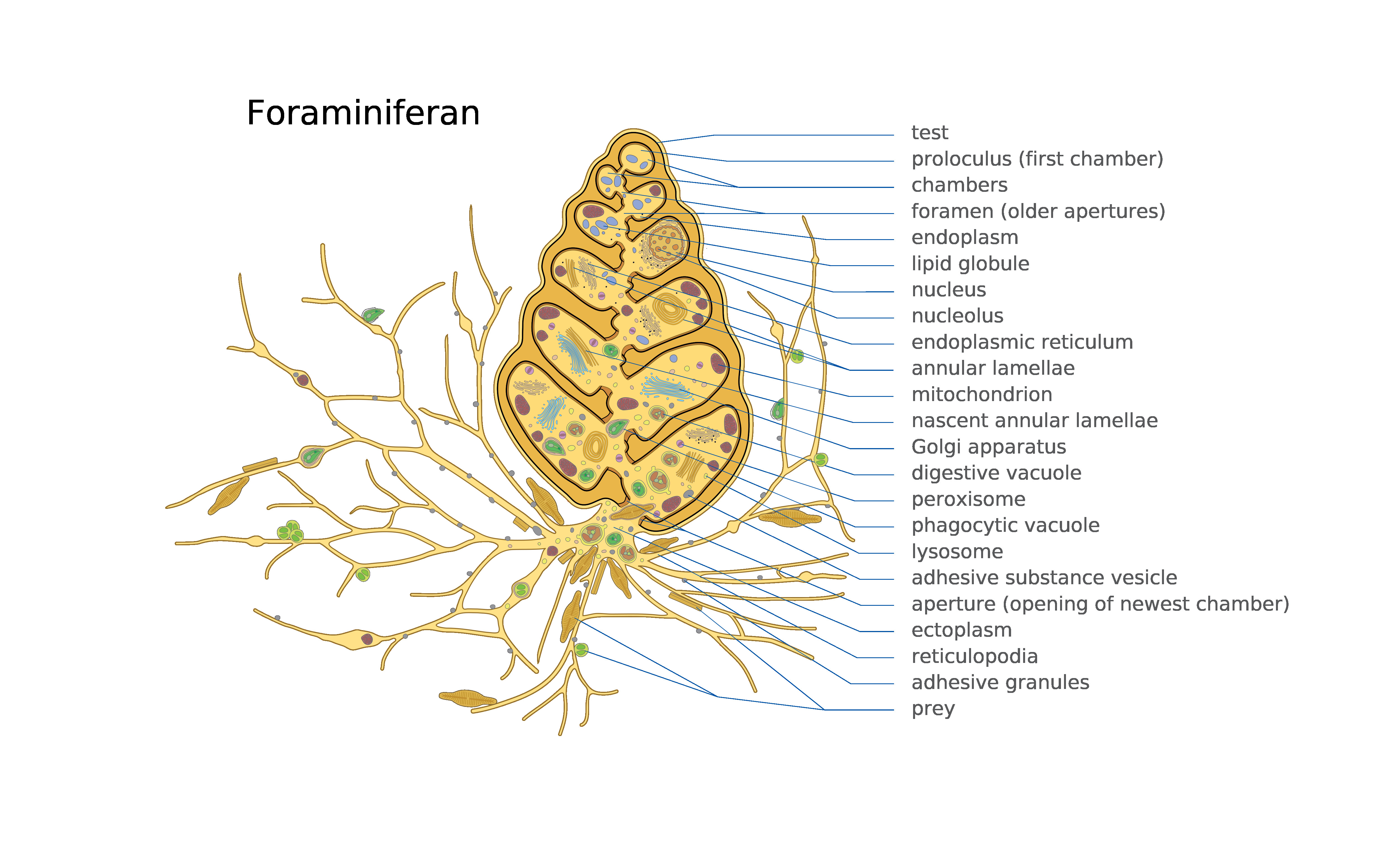

Supplement: S6 File — (ZIP) [file pbio.3002395.s006.zip › 2023 Pictures JPG files/2023 Foram.jpg]

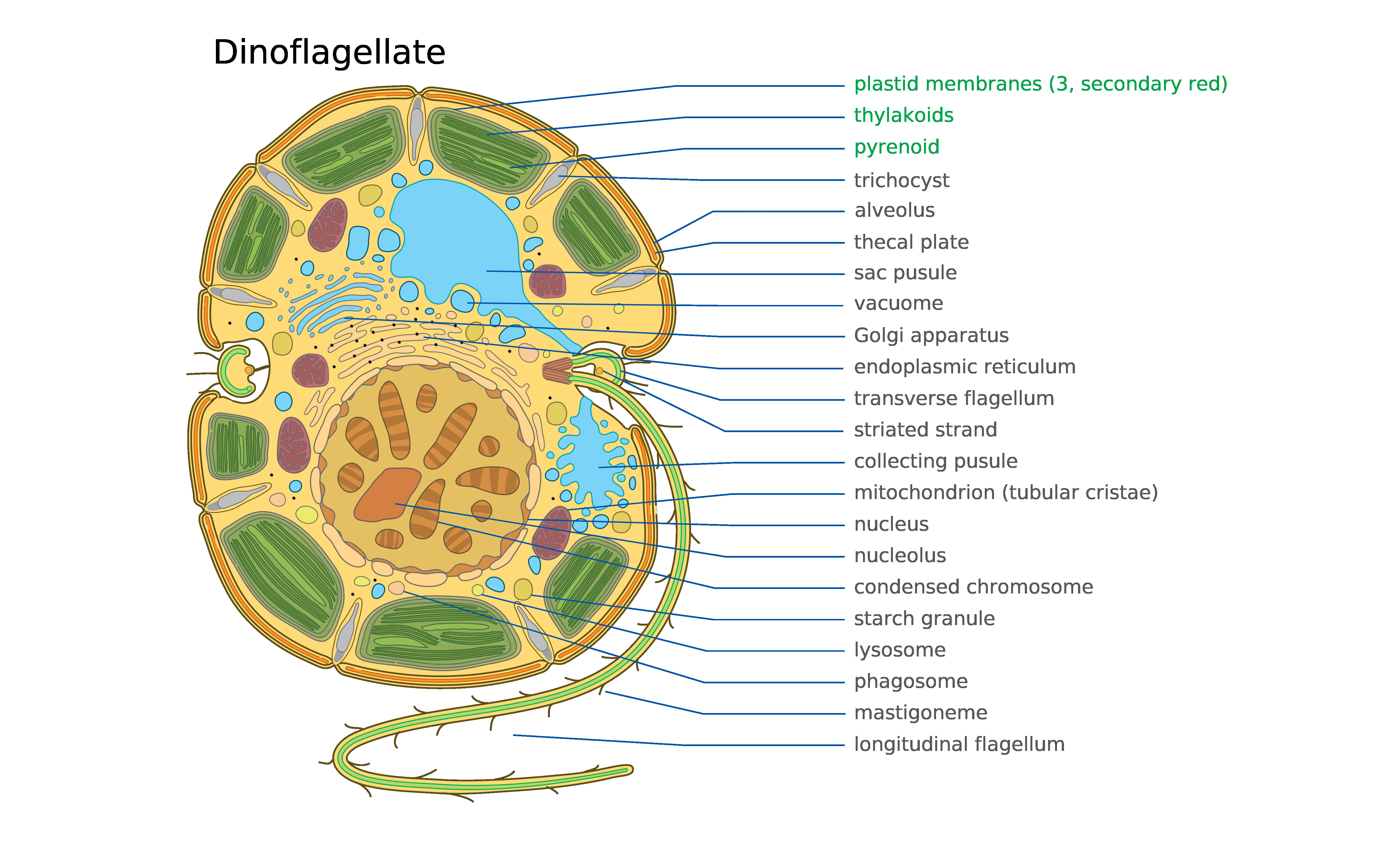

Supplement: S6 File — (ZIP) [file pbio.3002395.s006.zip › 2023 Pictures JPG files/2023 Dinoflagellate.jpg]

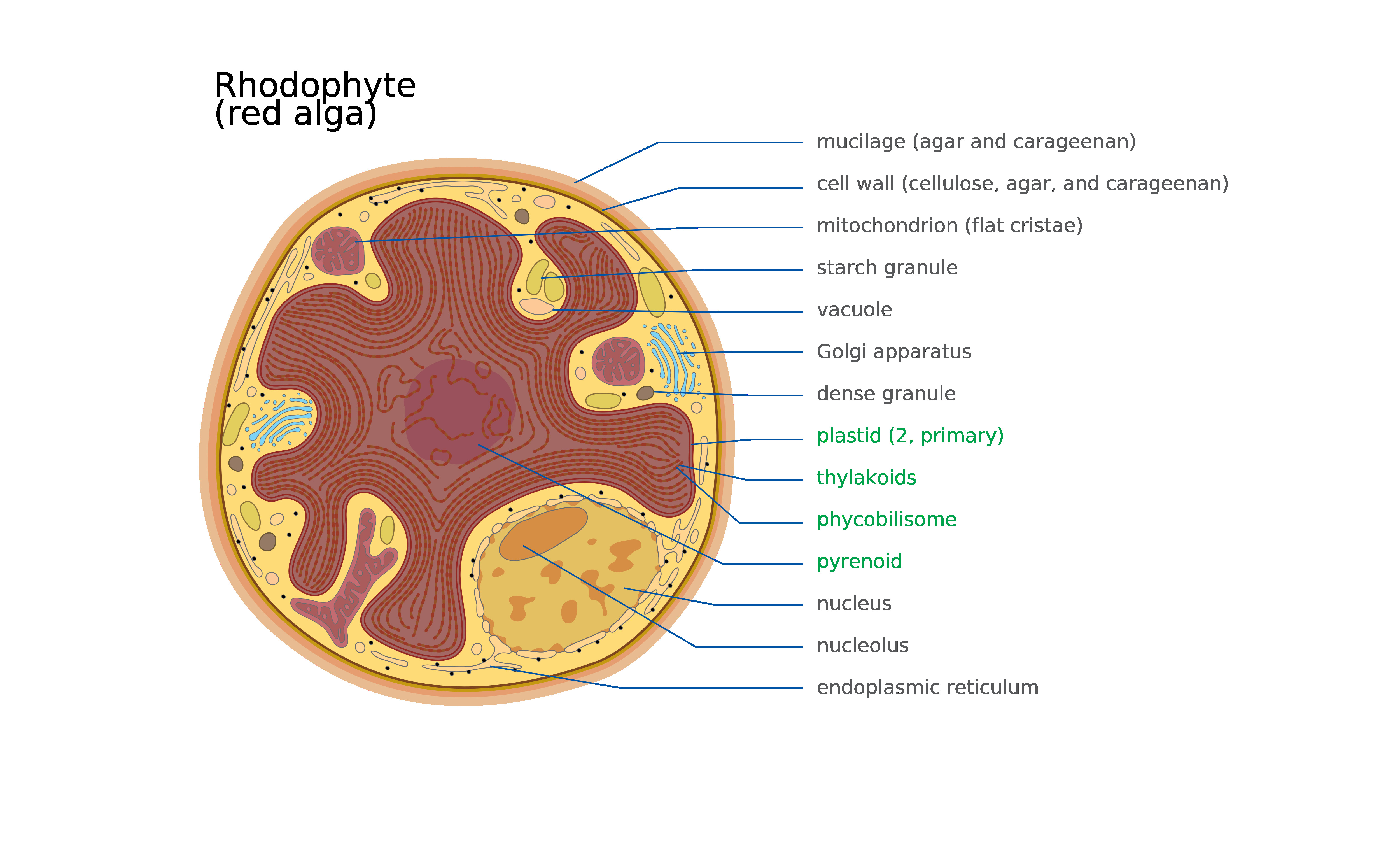

Supplement: S6 File — (ZIP) [file pbio.3002395.s006.zip › 2023 Pictures JPG files/2023 Rhodophyte.jpg]

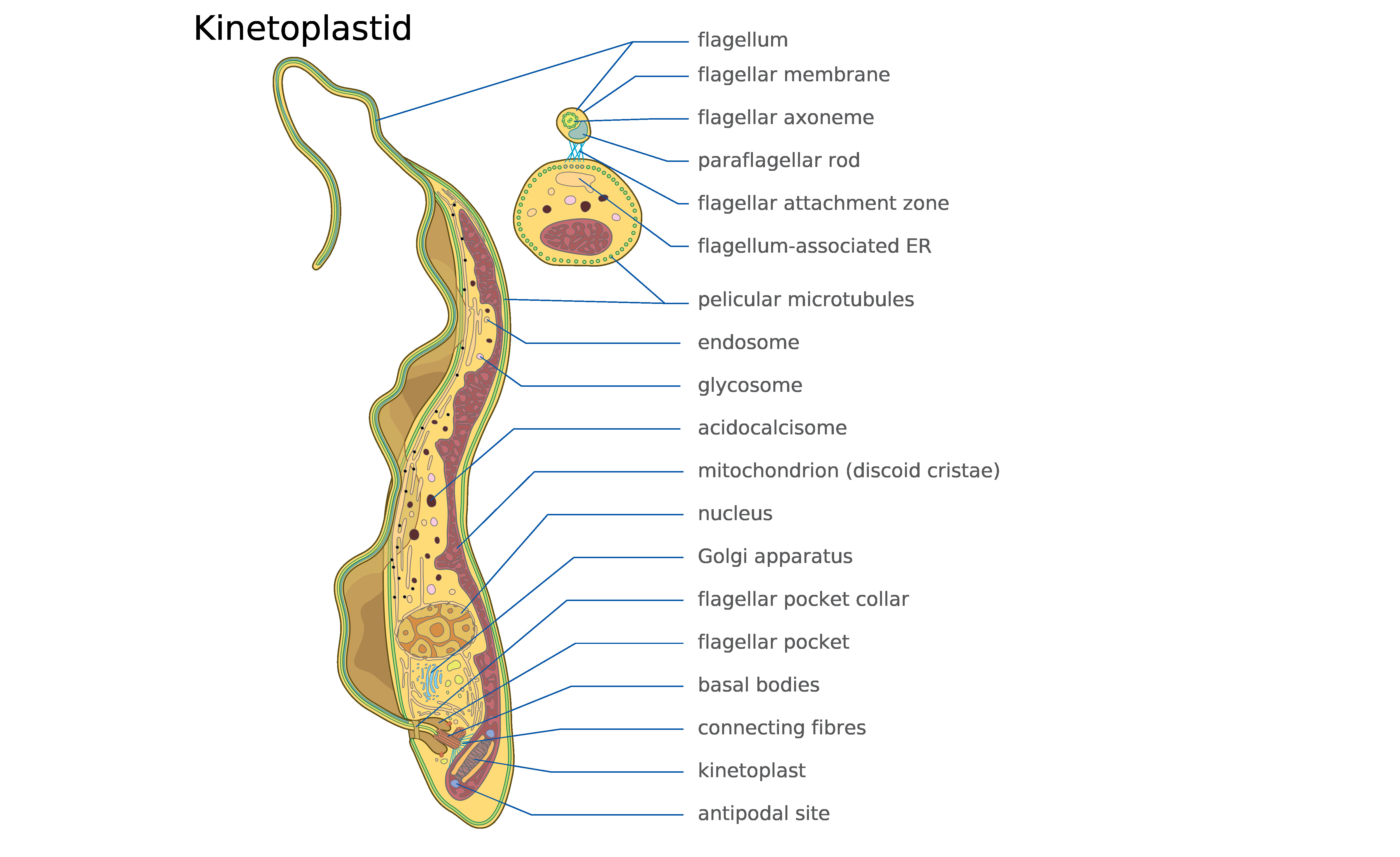

Supplement: S6 File — (ZIP) [file pbio.3002395.s006.zip › 2023 Pictures JPG files/2023 Kinetoplastid.jpg]

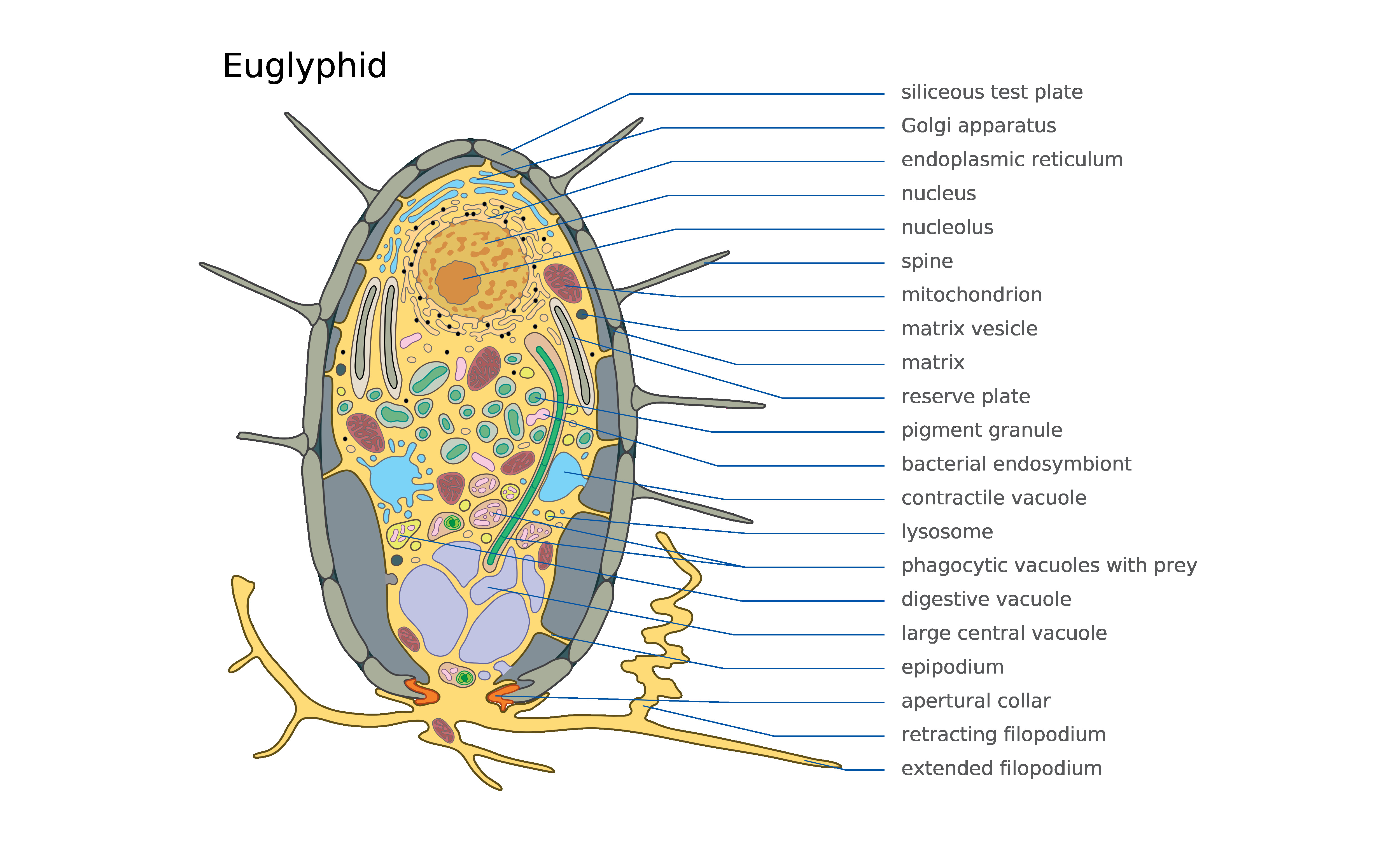

Supplement: S6 File — (ZIP) [file pbio.3002395.s006.zip › 2023 Pictures JPG files/2023 EuglyphidYEpk.jpg]

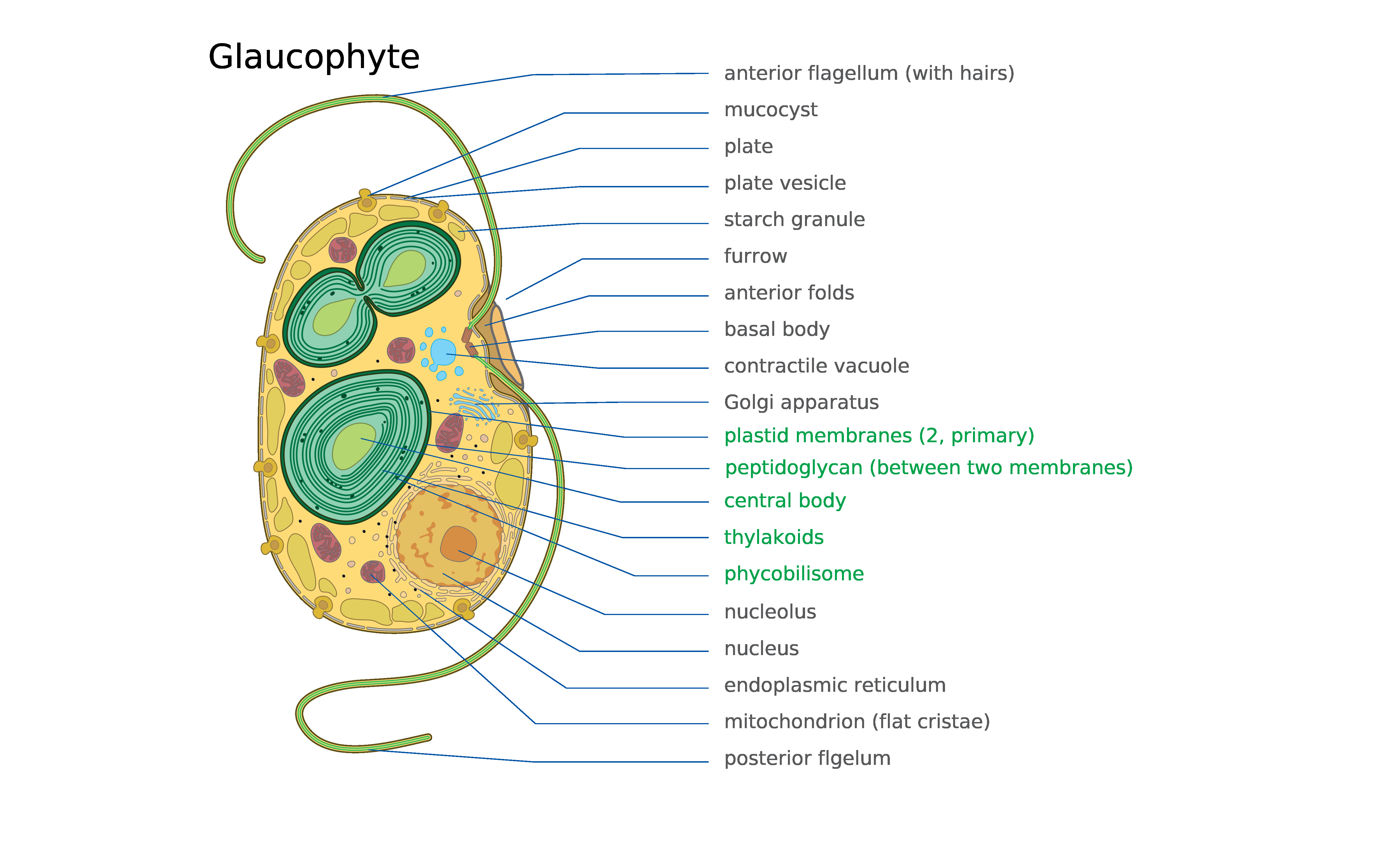

Supplement: S6 File — (ZIP) [file pbio.3002395.s006.zip › 2023 Pictures JPG files/2023 Glaucophyte.jpg]

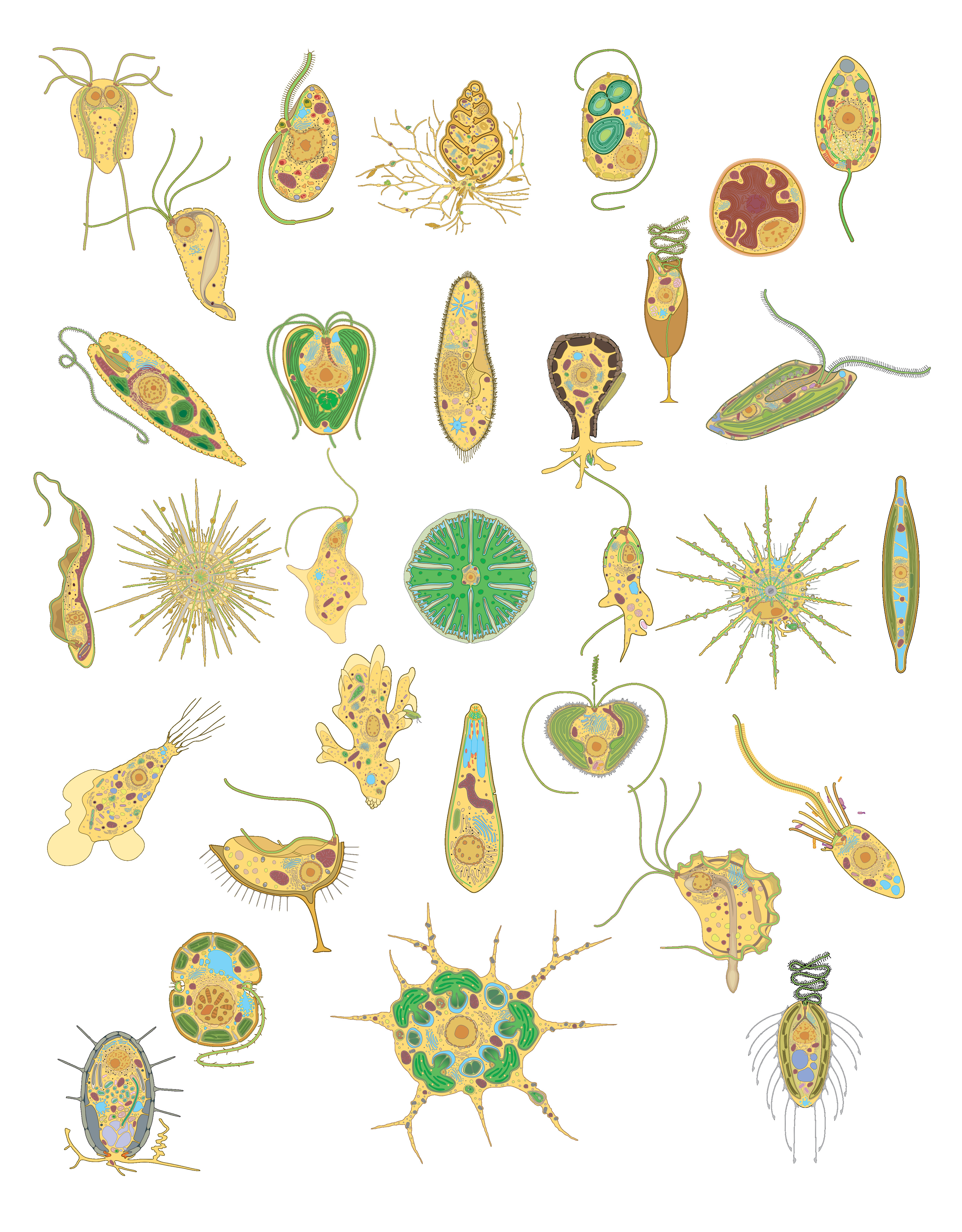

Supplement: S6 File — (ZIP) [file pbio.3002395.s006.zip › 2023 Pictures JPG files/2023 Figure 4 Montage.jpg]

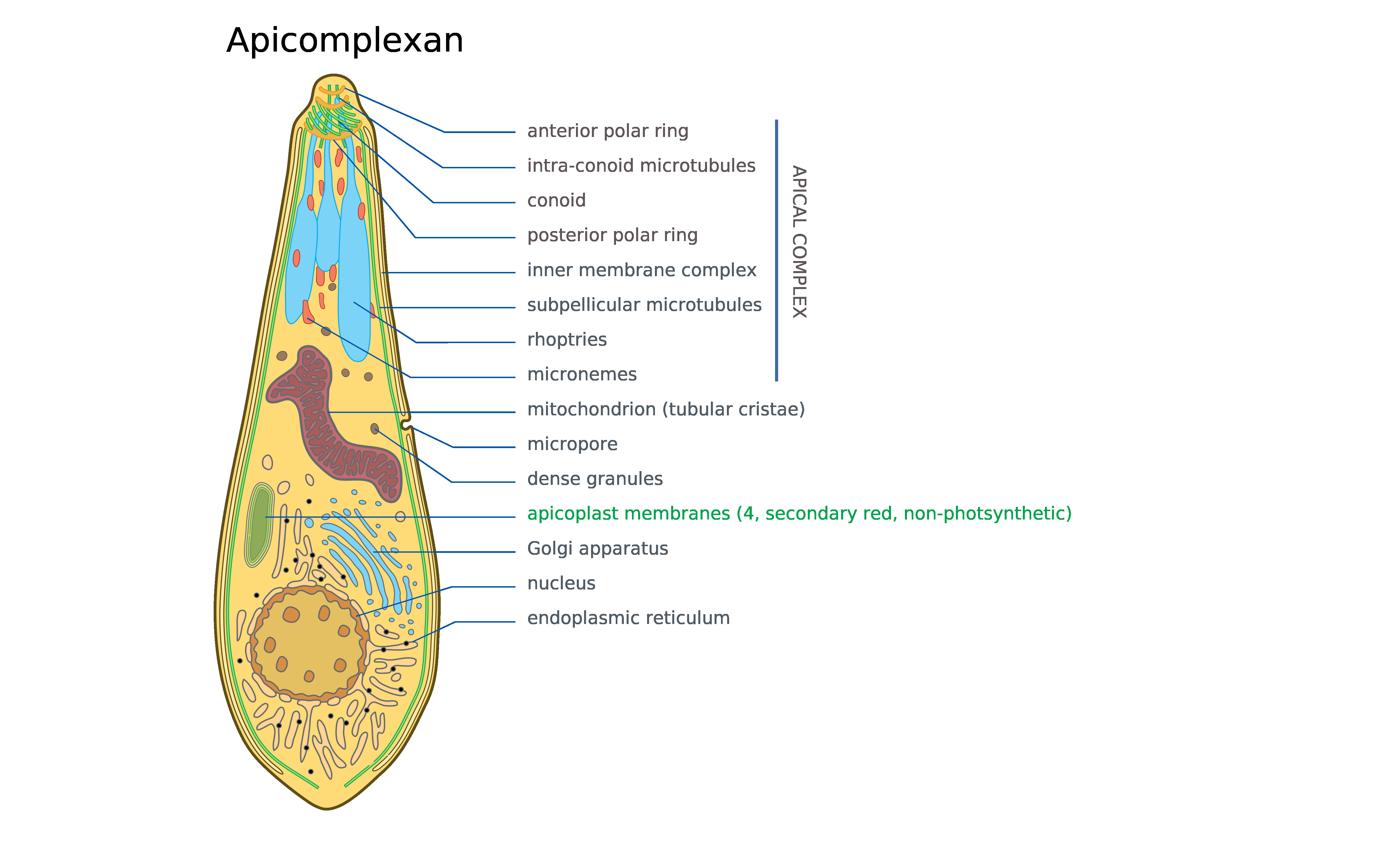

Supplement: S6 File — (ZIP) [file pbio.3002395.s006.zip › 2023 Pictures JPG files/2023 Apicomplexan.jpg]

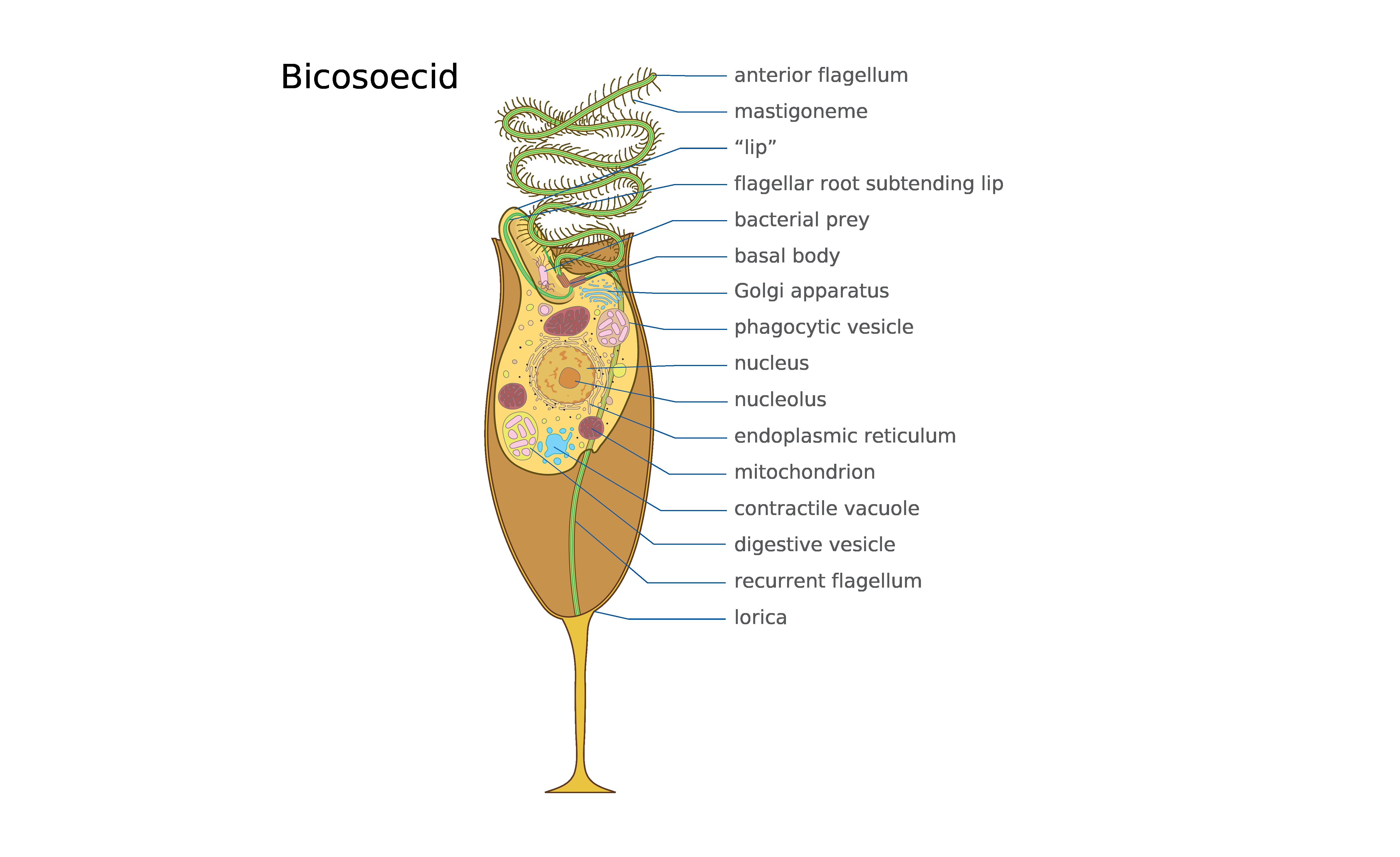

Supplement: S6 File — (ZIP) [file pbio.3002395.s006.zip › 2023 Pictures JPG files/2023 Bicosoecid.jpg]

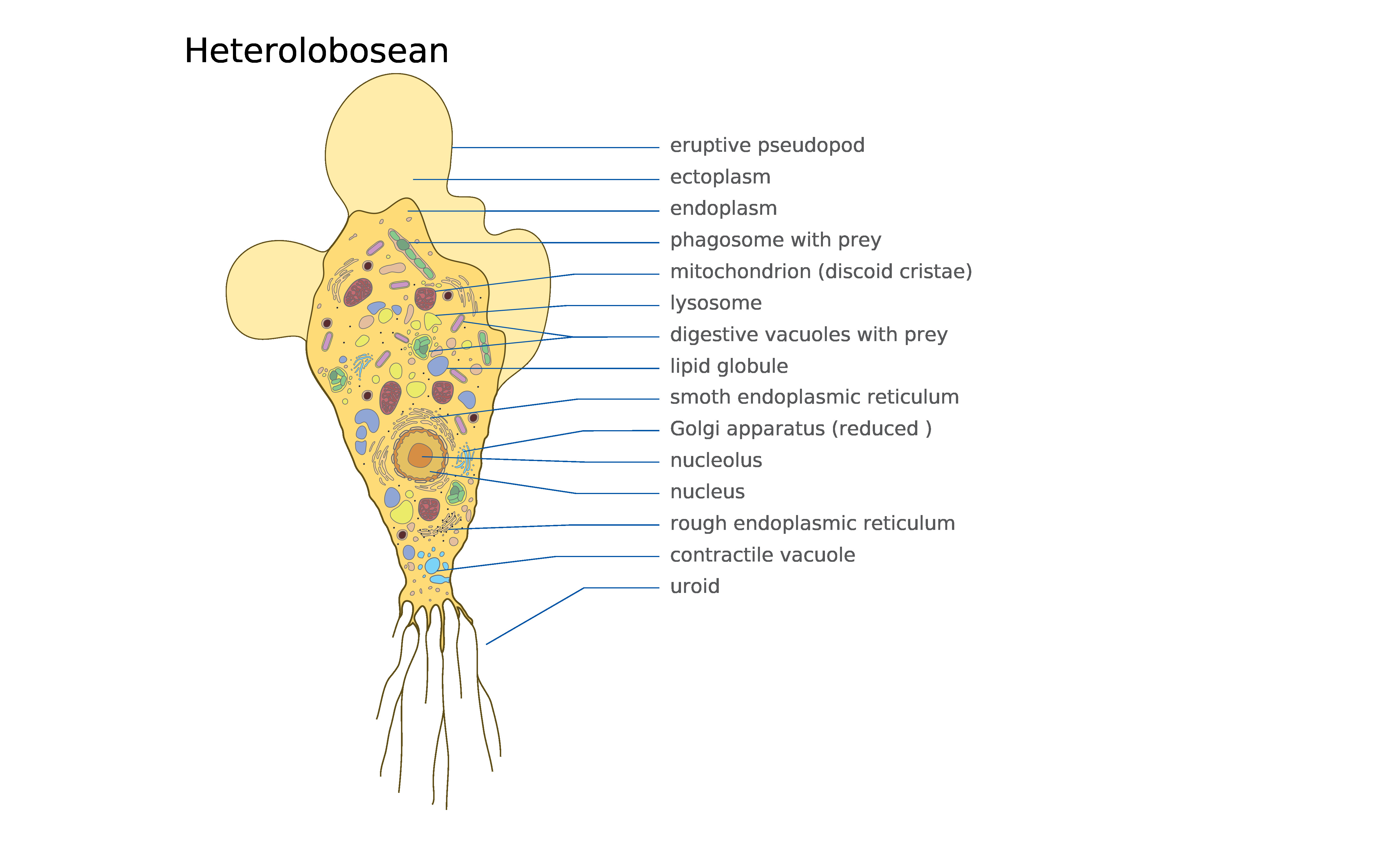

Supplement: S6 File — (ZIP) [file pbio.3002395.s006.zip › 2023 Pictures JPG files/2023 Heterolobosean.jpg]
